# Inverse scattering in biological samples via beam propagation

**DOI:** 10.1126/sciadv.aec8678

**Published:** 2026-08-21

**Authors:** Jeongsoo Kim, Blythe Bolton, Khashayar Moshksayan, Rishika Khanna, Mary E. Swartz, Michał Ziemczonok, Mohini Kamra, Karin Allenspach, Sapun H. Parekh, Małgorzata Kujawińska, Johann K. Eberhart, Elif Sarinay Cenik, Adela Ben-Yakar, Shwetadwip Chowdhury

**Affiliations:** ^1^ Chandra Department of Electrical and Computer Engineering, University of Texas at Austin, Austin, TX, USA.; ^2^ Department of Molecular Biosciences, University of Texas at Austin, Austin, TX, USA.; ^3^ Walker Department of Mechanical Engineering, University of Texas at Austin, Austin, TX, USA.; ^4^ Waggoner Center for Alcohol and Addiction Research, University of Texas at Austin, Austin, TX, USA.; ^5^ Warsaw University of Technology, Institute of Micromechanics and Photonics, Warsaw, Poland.; ^6^ Department of Biomedical Engineering, University of Texas at Austin, Austin, TX, USA.; ^7^ Precision One Health Initiative, University of Georgia, Athens, GA, USA.

## Abstract

Multiple scattering limits optical imaging in thick biological samples by scrambling sample-specific information. Physics-based inverse-scattering methods aim to computationally unscramble this information often by using nonconvex optimization solvers. However, their inherent nonconvexity often leads to highly sample-dependent performance and inaccurate reconstructions, particularly in strongly scattering specimens. Here, we introduce a novel inverse-scattering framework based on multislice beam propagation (MSBP) that robustly achieves high-quality scatter correction and label-free volumetric imaging across a diverse range of scattering biological samples. We rigorously benchmarked imaging performance across multiple MSBP solver implementations using both scattering calibration phantoms and biological specimens. We found that an amplitude-only cost function in the inverse solver, combined with angular and defocus diversity in the scattering measurements, enabled volumetric, label-free imaging with high-quality and subcellular-level scatter correction. Together, these results establish a foundation for the reliable application of inverse scattering to achieve biologically interpretable three-dimensional imaging in increasingly thick, multicellular samples, thus introducing a new paradigm for deep-tissue computational imaging.

## INTRODUCTION

Optical microscopy is a major research tool in the biosciences and enables both morphological and molecular imaging at subcellular spatial resolutions and high speeds. However, a major challenge is optical scattering, which scrambles sample-specific information and degrades imaging quality starting at depths of even just a few tens of micrometers into biological tissue. Thus, classical microscope techniques are typically used to image only outer superficial tissue layers, or specific components isolated from within tissue and prepared in vitro.

In response to this challenge, deep-imaging techniques (e.g., multiphoton microscopy, optical coherence tomography, etc.) have emerged, which extend imaging depths to the order of multiple hundreds of micrometers, made possible by selectively exciting or detecting with only unscattered (i.e., ballistic) light. These deep-tissue imaging techniques have found enormous success in the biosciences in enabling up to millimeter-scale imaging depths in fields such as neuroscience (*1*, *2*), developmental biology (*3*, *4*), and biomedicine (*5*, *6*). However, the ballistic component of the total signal decreases exponentially with depth—as a result, these deep-imaging techniques are still fundamentally constrained by scattering.

Although scattered light appears random, it deterministically encodes sample-specific information. Furthermore, light can scatter through several millimeters in tissue before being absorbed. Thus, if one could fully unscramble the effects of scattering, one could in theory gain access to an imaging volume with more than an order-of-magnitude–longer imaging depth than that of current state-of-the-art deep-imaging techniques. Although this has yet to be achieved, it remains a powerful motivation that drives substantial excitement and ongoing efforts to develop methods for correcting optical scattering.

Recent works in computational optics have explored strategies to computationally correct for scattering effects, with the goal of reconstructing scatter-corrected and fully volumetric images. These frameworks often use physics-based scattering models to describe optical paths that light takes within scattering media. These models need to be invertible so that volumetric images of the sample can be reconstructed from an input set of raw scattering measurements via either analytic inversion or iterative optimization. This process is broadly referred to as solving the inverse-scattering problem and holds special importance even in fields outside optical imaging (*7*–*9*). Compared to adaptive optics technologies (*10*–*12*), which also aim to mitigate scattering effects but do so by characterizing accumulated scattering from a limited sample region, inverse-scattering methods seek to characterize the sample’s intrinsic scattering properties fully in three dimensions (3D). This is popularly accomplished by mapping out the sample’s 3D refractive index (RI), which fully characterizes the sample’s scattering behavior due to its mathematical equivalence to scattering potential. Notably, RI is also a powerful source of endogenous morphological contrast; thus, mapping out a sample’s 3D RI naturally provides scatter-corrected, label-free, and quantitative tomography of the sample’s 3D morphology. Because this method does not require guide stars, it is particularly promising for noninvasive imaging applications.

Various inverse-scattering methods have been developed to reconstruct a sample’s 3D RI from scattering measurements. In the simplest case when a sample is weakly scattering, scattering measurements can be linearly related to the sample’s 3D RI. This enables 3D RI to be directly reconstructed from measurement data via simple linear inversion. This strategy found early and substantial success in imaging applications involving thin biological samples (e.g., monolayer cell distributions, small multicellular samples, etc.) where imaging depths were typically a few tens of micrometers. Multiple groups have introduced variations of this strategy to reconstruct 3D RI under a variety of imaging configurations, such as interferometric (*13*, *14*) versus noninterferometric detection (*15*, *16*), monochromatic (*17*, *18*) versus broadband illumination (*19*, *20*), illumination-angle (*21*, *22*) versus axial-defocus scanning (*23*, *24*), planar (*25*, *26*) versus patterned illumination (*27*, *28*), etc. Many of these methods have matured to the point where they are robust enough for studying sensitive biological processes, which often demand long-term stable imaging with minimal phototoxicity.

However, the major limitation of the inverse-scattering methods described above is their reliance on the assumption that the sample is weak scattering, which is true for samples such as monolayer cell distributions. For thicker and larger-scale multicellular samples, which contain substantial multiple scattering, recent studies have introduced partially coherent RI tomography techniques that use coherence gating to selectively detect only the weak-scattered component of the total scattered light (*28*–*30*). This enables the continued use of weak-scattering assumptions even when reconstructing 3D RI in multicellular samples. However, as sample thickness increases, the weakly scattered signal may be rapidly overwhelmed by multiple scattering, eventually rendering it nearly undetectable.

More sophisticated models that mathematically account for varying degrees of multiple scattering have been introduced. For example, beam propagation methods—originally developed decades ago for modeling light transmission through atmospheric turbulence (*31*), graded-index fibers (*32*), microlenses (*33*), etc.—have recently been adapted into optical microscopy for 3D inverse scattering and RI reconstruction of multicellular organisms such as *Caenorhabditis elegans* (*C. elegans*) worms (*34*, *35*), mouse embryos (*36*), or zebrafish embryos (*37*), where optical thicknesses can range to >60 μm. Independent work by Osnabrugge *et al.* (*38*) introduced a separate inverse-scattering framework based on a modified Born series, which was later demonstrated for bioimaging by Lee *et al.* (*39*) and Li *et al.* (*40*), who show RI reconstructions in tissue sections, microalgae, and *C. elegans* worms. A series of works by Yasuhiko *et al.* (*41*–*43*) introduced another separate inverse-scattering strategy that computationally suppressed scattering effects via either coherent or incoherent accumulation, which could then be related to the sample’s 3D RI via deconvolution. Biological demonstrations were conducted with organoid samples, where imaging results included multicellular human hepatoma organoids and human brain glioblastoma spheroids with thicknesses greater than 130 μm. Other studies, including those by Liu *et al.* (*44*), Soubies *et al.* (*45*), and Pham *et al.* (*46*), have proposed inverse-scattering models that aimed to directly solve the scalar Helmholtz equation to capture fully volumetric scattering phenomena.

Notably, despite promising demonstrations, these types of inverse multiple-scattering methods have yet to gain widespread adoption in the basic-science community. A major reason is likely that their performance has not yet been characterized across diverse, real-world biological samples that exhibit varying degrees of multiple scattering. Moreover, the fundamental limits of these methods remain largely unexplored. For example, none have yet demonstrated fully volumetric millimeter-scale imaging depths despite such depths being theoretically within the multiple-scattering limit of most biological tissues. Unfortunately, robustly characterizing these inverse-scattering methods is challenging, largely because multiple scattering prevents an explicit analytical relationship between the scattering measurements and the sample’s 3D RI. As a result, these methods are often formulated as computational inverse problems, solved via nonconvex optimization. In such cases, 3D RI is often reconstructed by iteratively adjusting RI values of each voxel within the sample volume, with the goal of minimizing the difference between scattering measurements and predictions made by the scattering model. There is no mathematical guarantee that this strategy yields a converged solution that is physiologically true or even realistic. It is well-known in other fields that the converged solution to a nonconvex inverse problem is highly sensitive to factors such as the quality and quantity of input data, the choice of optimization algorithm, the conditions under which convergence is performed, the incorporation of appropriate computational priors, or even the complexity of the true solution itself (*47*–*49*). To the best of our knowledge, this issue has not yet been explored in the context of inverse scattering for optical imaging.

In this work, we investigate for the first time, to our knowledge, how various computational factors influence the converged 3D RI reconstruction using the multislice beam propagation (MSBP) method. MSBP is a relatively simple yet effective inverse-scattering method (*21*, *34*, *50*–*52*). However, as we will show here, MSBP suffers from local minima like most other nonconvex inverse problems. Specifically, we will show that the quality of image reconstruction is critically, and sometimes counterintuitively, affected by seemingly arbitrary choices of whether to use a complex-valued field versus real-valued amplitude cost function, computational zero padding, initial guess based on weak-scattering approximation, or optical defocusing. While prior studies have examined some of these factors individually (*21*, *23*, *34*, *50*, *53*, *54*), we present here, to our knowledge, the first unified and systematic evaluation of all of them together, across the broadest and most diverse set of multiple-scattering biological samples considered to date. We further identify and describe the reconstruction methodology empirically found to be most effective for consistently achieving high-quality scatter-correction and label-free imaging. Last, the data and code used to generate the results in this work have been made open source so that others can reproduce our findings as well as benchmark other inverse-scattering methods. Thus, this work represents an important advancement toward achieving robust scatter-correction and deep-tissue volumetric imaging in thick multicellular samples via computational inverse scattering.

## RESULTS

### Basic principles of computational optics

We begin by highlighting three computational optics techniques that form the foundation of our approach to robustly conduct inverse scattering in various multiple-scattering samples.

#### Computational propagation of fields

Propagation of an optical field through homogenous media can be efficiently performed computationally using principles from Fourier optics, which treat the field as a superposition of plane waves with different spatial frequencies (*55*). The process begins by taking a 2D spatial Fourier transform of the complex-valued field distribution at an initial plane, which decomposes it into its component plane waves (i.e., angular spectrum). Each plane wave component corresponds to a specific spatial frequency and propagation direction.

To propagate the field over a distance Δz, each plane wave is multiplied by a complex phase factor known as the transfer function of free space, which accounts for the phase shift accumulated by that corresponding wave during propagation. This transfer function depends on the spatial frequencies and the wavelength, typically expressed as

$$H_{\Delta z}\left(\vec{v}\right)=\text{exp}\left(j\;2\pi \cdot n_{0}\cdot \Delta z\;\sqrt{\frac{1}{\lambda^{2}}-{|\vec{v}|}^{2}}\right)$$ (1)

where $\vec{v}$ is the 2D transverse $\left(v_{x},v_{y}\right)$ spatial frequency of the component plane wave, λ is the wavelength, and $n_{0}$ is the RI of the homogenous media that the wave is propagating through. After applying this phase factor to each component plane wave, an inverse Fourier transform is performed to reconstruct the field after being propagated by distance Δz.

#### Inverse scattering based on weak-scattering approximation and angular scattering field measurements

For weakly scattering samples, the Fourier diffraction theorem provides a crucial relationship between the measured scattered fields and the sample’s 3D RI distribution in spatial-frequency space (k-space) (*56*, *57*). Specifically, under plane wave illumination from a given direction, the measured scattered field (after phase-unwrapping) maps to a spherical shell in the sample’s k-space, with the shell’s position determined by the incident and scattered wave vectors. Thus, each illumination angle maps sample-specific information to unique spherical shells in k-space.

By systematically varying the illumination angle and recording the resulting fields, different regions of k-space are sampled. As more illumination angles are used, these spherical shells accumulate, gradually filling k-space and enabling reconstruction of the full 3D RI distribution via inverse Fourier transform. This multiangle acquisition strategy is essential for accurate, high-resolution 3D RI imaging, as it allows the volumetric reconstruction of the object’s spatial frequency distribution from a series of 2D field measurements.

#### Inverse scattering with MSBP

At the core of our investigation is the MSBP method, which has recently shown strong potential for reconstructing 3D RI distributions in multiple-scattering regimes. MSBP models light-sample interactions by decomposing the 3D volume into a series of infinitesimally thin 2D layers, each acting as a phase-modulating mask. The sample’s bulk scattering characteristics are modeled by accumulating diffraction effects as light propagates layer-by-layer through the sample.

This light propagation process through the sample consists of two main steps: (i) wave-propagation through homogenous medium between adjacent 2D layers and (ii) phase modulation by the layer’s complex transmittance function. These steps are mathematically expressed as shown below.

Wave propagation

$$E_{m}^{\prime }\left(\vec{r}\right)=\left(h_{\Delta z}⊗E_{m-1}\right)\left(\vec{r}\right)$$ (2)

Phase modulation

$$E_{m}\left(\vec{r}\right)=T_{m}\left(\vec{r}\right)\cdot E_{m}^{\prime }\left(\vec{r}\right)$$ (3)

Here, $\vec{r}$ is the 2D traverse (x,y) spatial coordinates variable. $h_{\Delta z}$ denotes the optical propagation kernel by the distance Δz separating the layers and is the inverse Fourier transform of Eq. 1. The operator ⊗ represents mathematical convolution. $E_{m}$ and $E_{m}^{\prime }$ denote the outgoing and incoming 2D electric fields at the mth layer of the 3D sample, respectively. This mth sample layer is modeled as a phase-mask with the 2D complex transmittance function $T_{m}\left(\vec{r}\right)=\text{exp}\left[j\;\Delta z\;k_{0}\;\Delta n_{m}\left(\vec{r}\right)\right]$, where $k_{0}=2\pi n_{0}/\lambda$ denotes wave number, and $\Delta n_{m}\left(\vec{r}\right)$ denotes the RI difference between the *m*th layer of the sample and the surrounding media with homogenous RI, i.e., $\Delta n_{m}\left(\vec{r}\right)=n_{m}\left(\vec{r}\right)-n_{0}$.

Equation 2 describes that the field incoming to the sample’s mth layer is simply the outgoing field from the previous (m−1)th layer, propagated by the distance Δz between layers. Equation 3 computes the outgoing field from the mth layer by simply multiplying the incoming field with the layer’s transmittance function. Thus, by repeatedly implementing Eqs. 2 and 3 an illumination wave incident to the sample can be fully propagated through all sample layers before being measured. This constitutes the MSBP forward model, which takes as input a 3D RI distribution as well as an incident wave, and computes the scattered field after the incident wave propagates all the way through. This sequence defines the MSBP forward model, which predicts the scattering measurement from a given 3D RI distribution and incident illumination. The goal of inverse scattering is to “invert” this forward model and reconstruct the sample’s RI from collected measurements. This is often implemented as a least squares problem that iteratively searches for a solution to the sample’s 3D RI distribution that minimizes the difference between experimental measurements and model predictions

$$\hat{n}\left({\vec{r}}_{3D}\right)=\underset{n\left({\vec{r}}_{3D}\right)}{\text{argmin}}\left[\frac{1}{2L}\sum_{\ell =1}^{L}{\left\|y^{\left(\ell \right)}\left(\vec{r}\right)-G^{\left(\ell \right)}\left\{n\left({\vec{r}}_{3D}\right)\right\}\right\|}_{L2}^{2}+\tau \cdot R\left\{n\left({\vec{r}}_{3D}\right)\right\}\right]$$ (4)

**Fig. 1.**
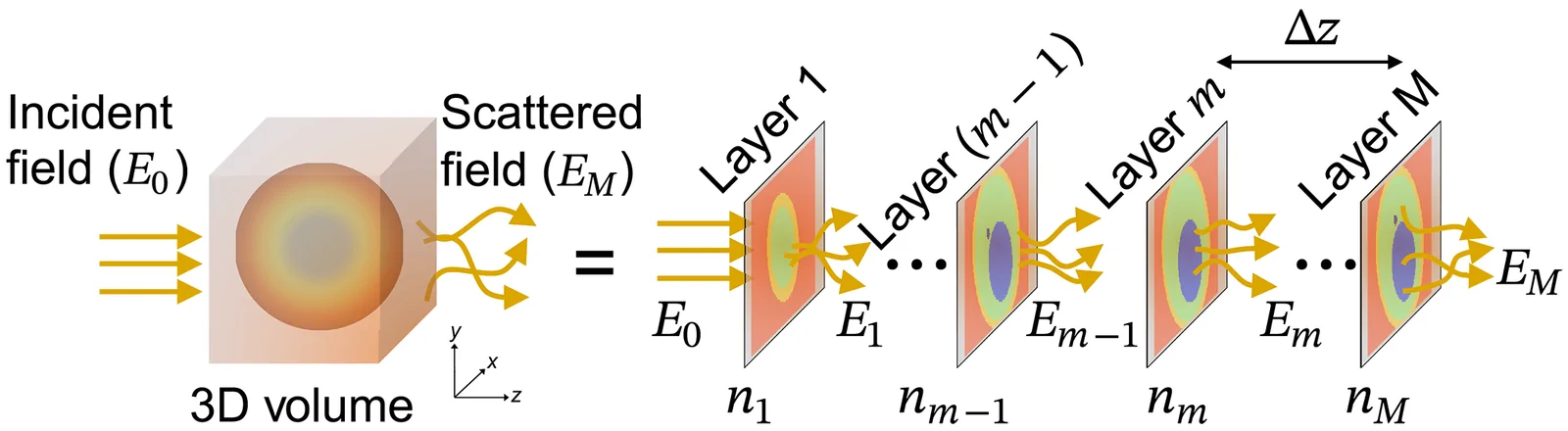
Schematic of MSBP forward model. MSBP forward model treats 3D object as multiple thin 2D transmission layers and scattered field is modeled via sequential layer-to-layer electric-field propagation through the sample.

Here, ${\vec{r}}_{3D}$ denotes the 3D spatial coordinate (x,y,m), where m indexes the axial layers of the sample, as illustrated in Fig. 1, i.e., $n\left({\vec{r}}_{3D}\right)=n_{m}\left(\vec{r}\right)$ for m=1,2,…M for a M-layer sample. The MSBP forward model is represented by the operator G, which encapsulates the repeated application of Eqs. 2 and 3, based on some estimate of $n\left({\vec{r}}_{3D}\right)$. The raw scattering measurement is denoted by $y\left(\vec{r}\right)$, with the parameter ℓ indexing the measurement (described below). Last, ${\left\|\ldots \right\|}_{L2}$ denotes the L2-norm, and R{.} represents a regularization operator to stabilize convergence. While various regularization strategies are available, in this work, we use total variation. The parameter τ controls the strength of regularization and is empirically tuned. We solve the minimization-based inverse problem in Eq. 4 using gradient descent, which also requires an empirically selected step size. To identify an effective combination of step size and regularization strength, we performed a series of MSBP-based inverse-scattering RI reconstructions on calibration microspheres with known RIs. We selected the parameter pair that yielded quantitatively accurate RI reconstructions across all reference spheres (see fig. S1).

Reconstructing the sample’s 3D RI distribution is the primary objective of inverse scattering. If successfully reconstructed, this RI distribution can be directly interpreted as a volumetric image of the sample that provides label-free and quantitative contrast of its endogenous morphology.

### Strategies for MSBP-based inverse scattering

Because of the inherent complexity of light scattering, solving for 3D RI from 2D measurements using Eq. 4 is an ill-posed problem. To improve its conditioning and enable more reliable reconstruction, it is essential to introduce diversity in the measured data. Below, we detail our data acquisition and preprocessing strategies designed to enhance measurement diversity, along with empirical observations into which approaches most effectively stabilized convergence.

1) Field versus amplitude-only measurements: The scattering measurement $y^{\left(\ell \right)}\left(\vec{r}\right)$ can be acquired either as complex-valued field or real-valued amplitude (i.e., square root of intensity) measurements. The forward model must be adapted accordingly. Let $E_{M}^{\left(\ell \right)}\left(\vec{r}\right)$ denote the field exiting the final (Mth) layer of an M-layer sample. In the case when directly using complex-valued field measurements (neglecting defocusing and resolution filtering) in the Eq. 4 inverse solver, the forward model corresponds directly to this field: $G^{\left(\ell \right)}\left\{n\left({\vec{r}}_{3D}\right)\right\}=E_{M}^{\left(\ell \right)}\left(\vec{r}\right)$. In contrast, when using amplitude-only measurements in Eq. 4, the forward model returns the magnitude of the field, i.e., $G^{\left(\ell \right)}\left\{n\left({\vec{r}}_{3D}\right)\right\}=|E_{M}^{\left(\ell \right)}\left(\vec{r}\right)|$.

2) Measurements with angled illumination and sample defocus: Diversity in the scattering measurements can be achieved by illuminating the sample at different angles and/or axially defocusing the sample. In the case of angular plane-wave illumination, the field before the sample’s first layer can be expressed as $E_{0}^{\left(\ell \right)}\left(\vec{r}\right)=\text{exp}\left[j\;{\vec{k}}_{0}^{\left(\ell \right)}\cdot \vec{r}\right]$, where ${\vec{k}}_{0}^{\left(\ell \right)}$ denotes the lateral wave vector of the plane-wave corresponding to the ℓth illumination angle. The forward model naturally accounts for this by using Eqs. 2 and 3, to propagate $E_{0}^{\left(\ell \right)}\left(\vec{r}\right)$ through the sample. To model sample defocus, an additional propagation step (i.e., Eq. 1) can be applied to the field exiting the sample, effectively shifting the sample to the desired imaging plane.

3) Initialization with weak-scattering approximation: Reconstructing the sample’s RI from Eq. 4 involves iteratively updating voxel-wise RI values within the sample’s volume until convergence. This process requires an initial RI estimate. Because of the nonconvex nature of the scattering problem, the final solution may depend on this initialization. Prior works often initialize MSBP with a uniform RI equal to the background medium (*34*). Here, we compare that approach to one using a weak-scattering estimate derived via the Fourier diffraction theorem and Rytov approximation (*56*–*58*). Notably, this strategy phase-unwraps the measured fields before using them to reconstruct the weak-scattering initialization. MSBP then refines this initialization.

**Fig. 2.**
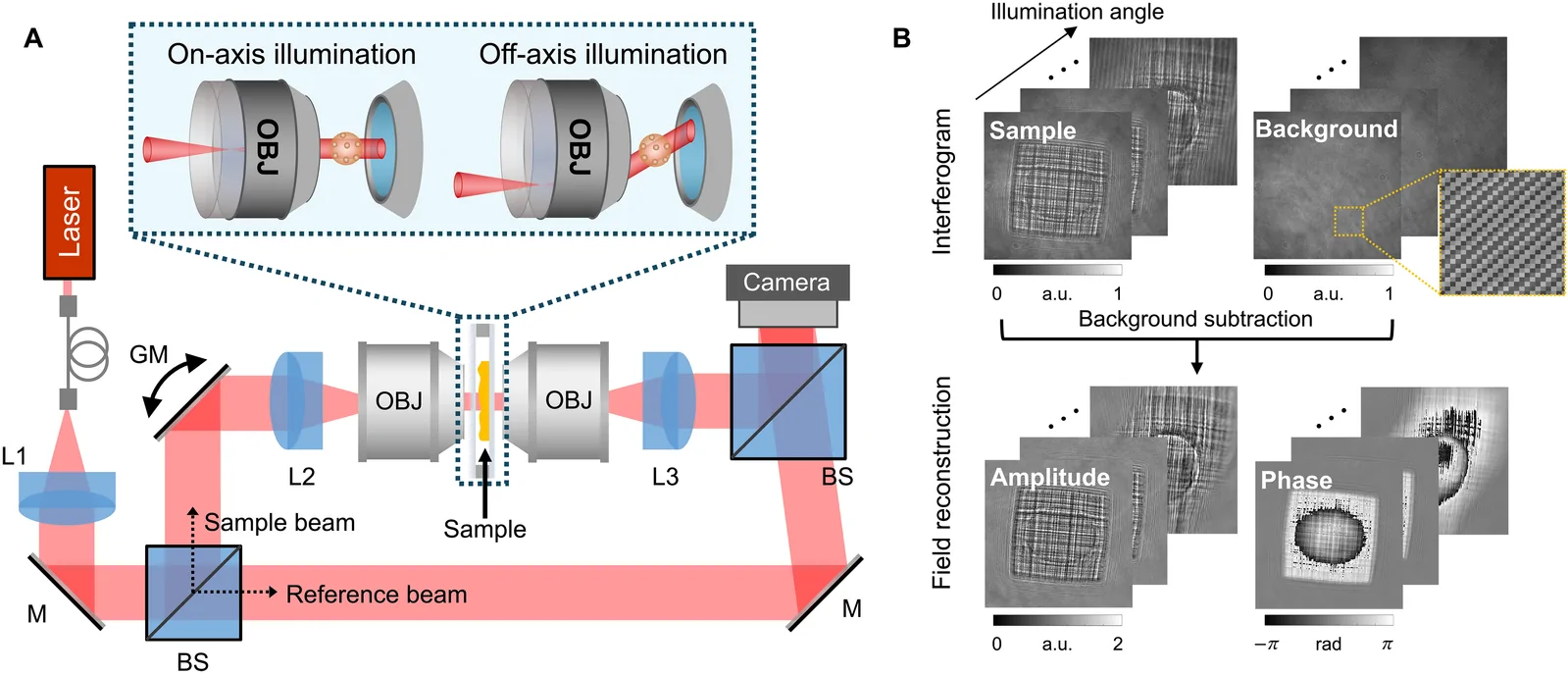
Schematic of the experimental setup and data acquisition framework. (**A**) Mach-Zehnder off-axis interferometer system with angularly scanned illumination. (L, lens; M, mirror; BS, beam splitter; GM, galvo mirror; OBJ, objective lens). (**B**) Example of data acquisition using a microphantom. Interferograms of the sample and background are sequentially captured at multiple illumination angles, followed by reconstruction of 2D amplitude and phase maps of the sample for each angle. a.u., arbitrary unit.

To acquire diverse scattering measurements, we use an interferometric system with angle-scanning capabilities that allow precise tilting of the illumination onto the sample (see Fig. 2). The scattered light is interfered with an off-axis reference beam, and standard digital filtering isolates the complex-valued scattered field (amplitude and phase) in k-space (*59*). This process is repeated with the sample translated out of view to collect background field measurements. Sample field measurements can then be normalized by complex-dividing with the background fields. Amplitude-only “measurements” can be synthetically generated by simply taking the amplitude component of the complex-valued field measurement.

The normalized angular scattering field measurements can be used with the Fourier diffraction theorem to estimate the sample’s 3D RI under the weak-scattering approximation. To bypass this approximation, MSBP can be used to reconstruct RI using either complex-valued field data or amplitude-only measurements. In addition, digital defocus can be introduced by computationally propagating the measured field, effectively allowing RI to be reconstructed from measurements synthetically collected at various focal planes. As a result, field-based, amplitude-only, and defocus-varied reconstructions can all be performed from a single-measurement dataset without having to physically reacquire images. This provides a fair and consistent basis for comparing the different reconstruction strategies that we explore below.

### Inverse scattering with complex-valued field versus amplitude-only measurements

We begin by comparing the reconstruction performance of MSBP-based inverse scattering using either complex-valued field data or amplitude-only measurements. Note that this choice results in slight modifications to the cost function as described previously, thereby altering the inverse problem in Eq. 4. We will show that this slight modification yields dramatic changes in 3D RI reconstruction.

We note that previous studies have performed MSBP-based 3D RI reconstruction using either complex-valued field data (*50*, *60*) or amplitude-only measurements (*34*, *35*). However, these two approaches have not been directly compared on a multiple-scattering sample under identical experimental conditions. As a result, it has remained unclear under which circumstances one strategy may outperform the other. In this section, we present a side-by-side comparison of both methods across real-world samples with varying scattering properties. This analysis allows us to establish a practical framework for achieving robust RI reconstruction across a range of scattering samples.

#### Comparison of MSBP-based inverse-scattering strategies with 3D-scattering microphantoms

**Fig. 3.**
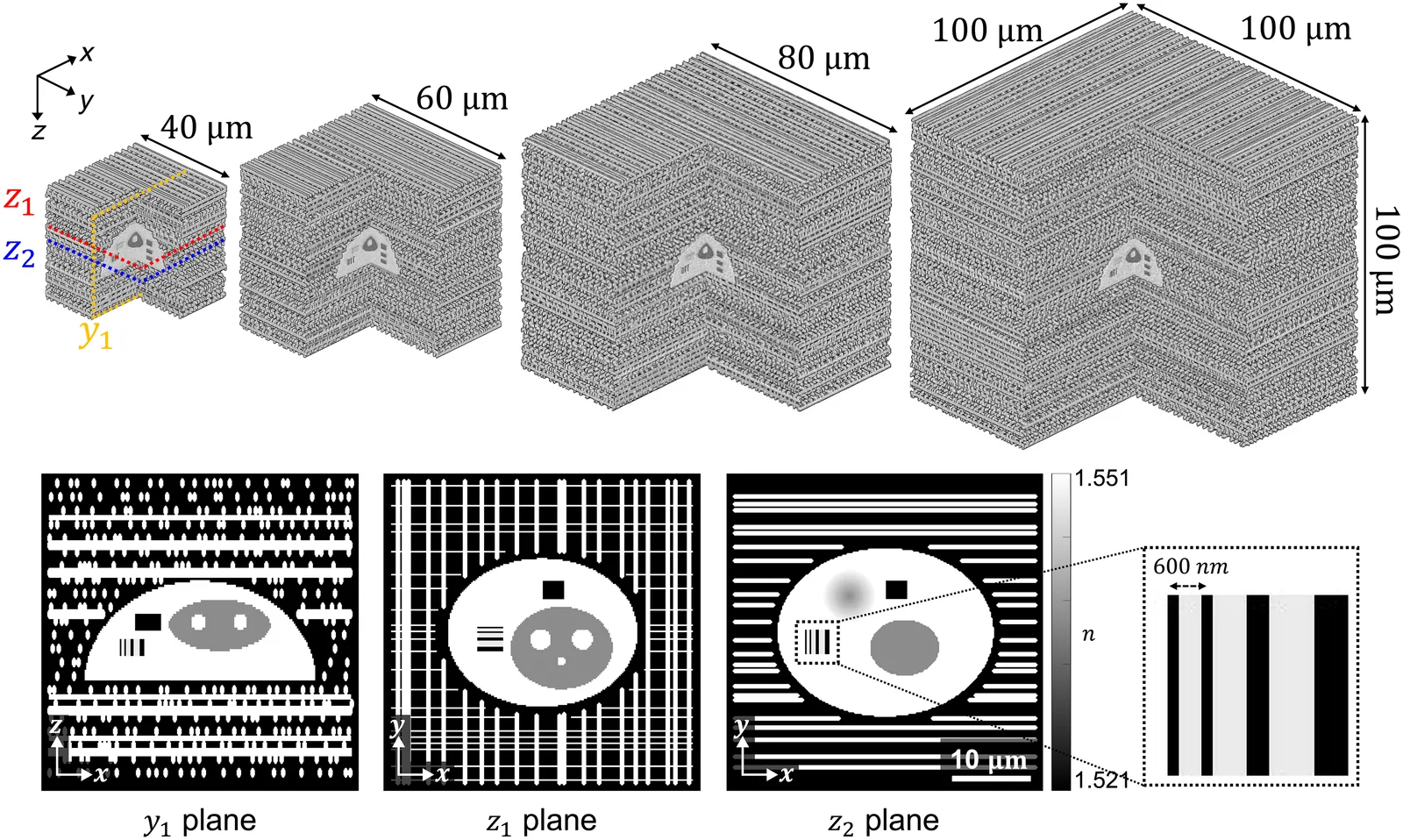
Design of a 3D scattering phantom for controlled multiple scattering. The phantom features an internal test structure embedded within a pseudo-random array of rods engineered to scatter light and obfuscate the imaging target. The extent of multiple scattering is tunable by varying the dimensions of the surrounding scattering volume.

*Phantom*. We first conduct these comparison experiments using 3D-scattering phantoms consisting of a 3D cell-imaging target embedded into scattering cubes of increasing size. Phantoms were 3D-printed (further described in Materials and Methods), and, thus, internal microstructures have known geometry and 3D RI distribution (*61*). Figure 3 shows the cell-imaging target, which contains internal test structures of known RI distributed in 3D, with the smallest feature size measuring 300 nm. The surrounding scattering cubes are composed of pseudo-randomly distributed rods designed to induce multiple scattering. By varying the cube size (40, 60, 80, and 100 μm), the scattering strength of the phantoms can be systematically controlled; i.e., the larger the cube, the greater the degree of multiple scattering. This makes the phantoms a robust platform for characterizing and identifying artifacts in 3D RI reconstructions under varying levels of multiple scattering. As described previously, complex-valued field measurements of the phantoms were acquired interferometrically under varying illumination angles. Corresponding amplitude-only measurements were synthetically derived by extracting the magnitude component from the complex field data.

**Fig. 4.**
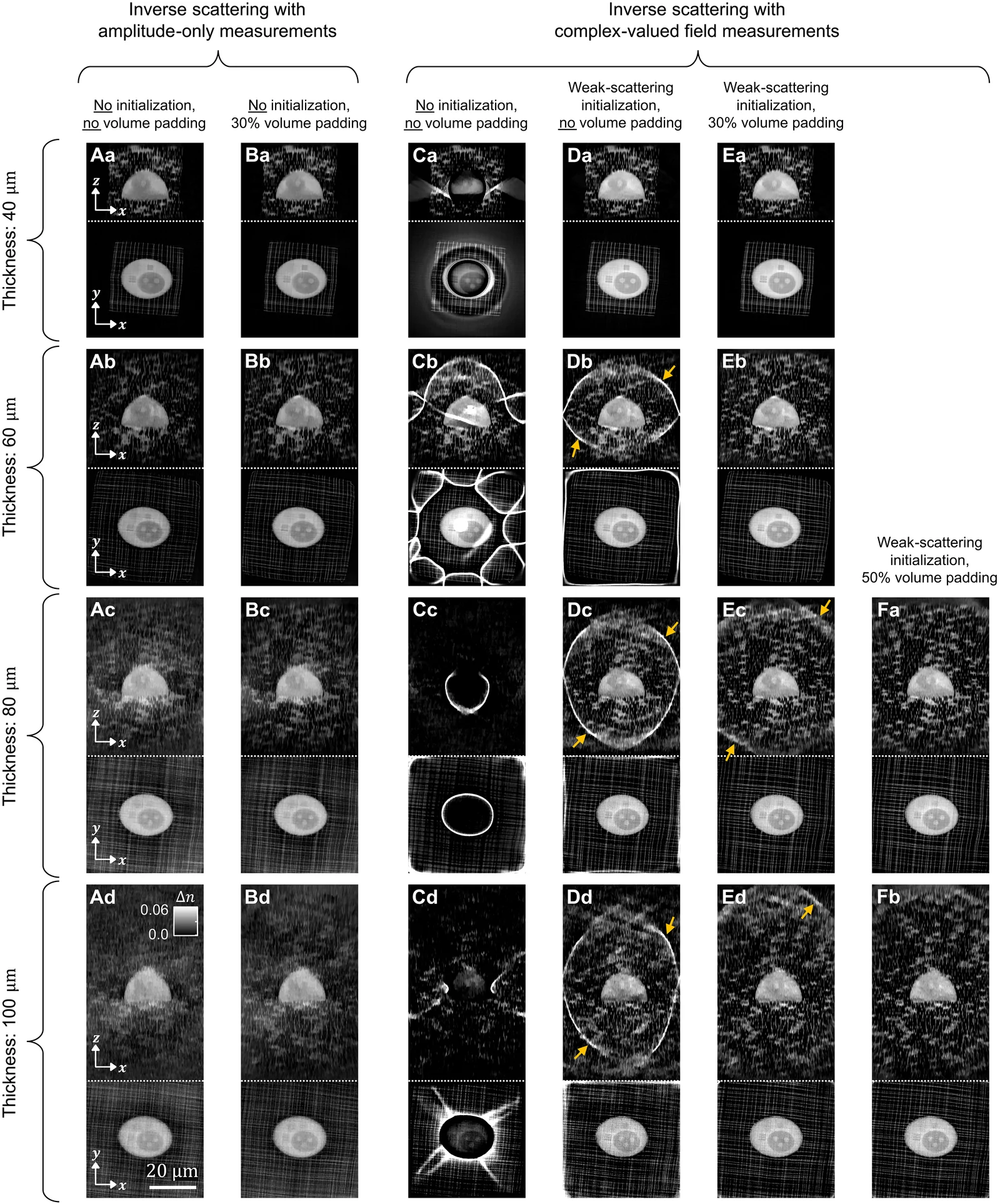
Comparison of 3D RI reconstruction results for 40-, 60-, 80-, and 100-μm scattering cube phantoms, using amplitude-only data versus complex-valued field measurements within the MSBP inverse solver framework. For amplitude-only data, the solver was initialized with a uniform RI distribution (Δn=0). 3D RI reconstruction results are shown (**Aa** to **Ad**) with and (**Ba** to **Bd**) without 30% volume padding. With complex-valued field measurements, 3D reconstruction results are shown with (**Ca** to **Cd**) Δn=0 RI initialization without padding, (**Da** to **Dd**) RI initialization with weak-scattering approximation without padding, (**Ea** to **Ed**) weak-scattering initialization +30% volume padding, and (**Fa** and **Fb**) weak-scattering initialization +50% volume padding. Yellow arrows in (Db) to (Dd) and (Ec) to (Ed) correspond to reconstruction artifacts from limited FOV.

*Imaging results*. Figure 4 (Aa to Ad) presents the 3D RI reconstruction results for the 40-, 60-, 80-, and 100-μm scattering phantoms using MSBP-based inverse scattering with amplitude-only data. To output these reconstructions, the inverse solver was initiated with a constant estimate of the sample’s 3D RI equal to that of the background medium (i.e., Δn=0). As the phantom size increased, resolving fine features became more difficult; however, the overall structures were recovered without prominent artifacts.

In contrast, Fig. 4 (Ca to Cd) shows the corresponding 3D RI reconstructions using complex-valued field measurements, where the inverse-solver was also initialized with a Δn=0 RI distribution. In this case, reconstruction performance deteriorates significantly, even for the smallest phantom. For example, ringing artifacts surrounding the imaging target appear in the 40-, 80-, and 100-μm cases, and additional spurious artifacts are observed spread across the reconstruction volume, particularly for the 60- and 100-μm phantoms. Visual inspection of the phase maps in the measurements (see fig. S2) reveals a strong spatial correlation between phase-wrapping artifacts and the ringing artifacts observed in the RI reconstructions. This relationship implies that phase wrapping introduces ambiguities in the measured phase, which can mislead the inverse solver and drive the reconstruction toward incorrect solutions. Similar effects are also seen in simulation, where MSBP reconstruction using complex-valued fields produces artifacts when the measurements exhibit phase wraps (fig. S3). Phase unwrapping does not resolve this issue because the MSBP model’s phase-modulation step in Eq. 3 inherently rewraps the phase at each sample layer by incorporating the RI term into the complex exponential.

To guide the reconstruction toward a more accurate solution, we next initialized the inverse solver with an estimate of the sample derived from the weak-scattering approximation. Specifically, we used the field measurements to compute a 3D RI estimate via the Fourier diffraction theorem, which then served as the initial guess for the iteration-based MSBP reconstruction. The resulting RI reconstructions are shown in Fig. 4 (Da to Dd). As expected, this warm initialization led to substantial improvements. In the 40-μm scattering phantom, the reconstruction is virtually artifact free. While artifacts persist in the larger phantoms, they are markedly reduced compared to those in Fig. 4 (Ca to Cd) and are no longer confined to regions near the imaging target, suggesting that a well-chosen initialization effectively mitigates ambiguities introduced by phase wrapping.

Notably, the remaining artifacts in Fig. 4 (Da to Dd), as indicated by yellow arrows, appear predominantly at the edges of the imaging field of view (FOV). This suggests that they are not intrinsic to the sample itself but rather a consequence of FOV limitations. This is consistent with the behavior of high-angle illumination, which can shift spatial information from sufficiently large samples to be partially outside the detectable FOV, thus resulting in missing information. This likely explains why the 40-μm phantom in Fig. 4Da shows no edge artifacts, while the larger phantoms do. To test this hypothesis, we zero-padded the reconstruction volume to mitigate edge-related artifacts while still keeping the weak-scattering initialization. Figure 4 (Ea to Ed) shows the results with 30% additional padding—the 40- and 60-μm phantoms are virtually artifact free, while the edge artifacts in the 80- and 100-μm reconstructions (indicated by yellow arrows) show substantial improvement. Increasing the padding further to 50% (Fig. 4, Fa and Fb) effectively eliminates the remaining edge artifacts, yielding visually artifact-free images for all phantom samples. As compared between Fig. 4 (Aa to Ad) and Fig. 4 (Ba to Bd), MSBP-based inverse scattering using amplitude-only measurements did not exhibit any noticeable edge artifacts and was not significantly affected by padding.

In summary, we empirically found that image clarity was slightly higher when solving the MSBP inverse problem using complex-valued field data compared to amplitude-only measurements. However, this improvement was only realized when the field data were combined with sufficient volume padding and a warm initial guess. Without these preprocessing steps, the reconstructions were degraded by artifacts. For our phantom sample, a weak-scattering approximation provided a sufficiently good initial guess. However, biological specimens may exhibit more complex scattering properties, making the weak-scattering approximation inadequate. In addition, larger samples may require extensive padding, which can significantly increase computational cost. In contrast, reconstructions using amplitude-only measurements required neither an initial guess nor volume padding to produce comparable image quality, thus offering a more computationally efficient approach less dependent on prior knowledge. We next investigate whether these trends persist in real-world biological specimens.

#### Comparison of MSBP-based inverse-scattering strategies with 3D scattering biological sample

We next examine whether the MSBP-based inverse-scattering reconstruction strategies demonstrated in phantoms translate effectively to a real-world biological sample. Here, we use an intestinal organoid immersed in phosphate-buffered saline (PBS) as an example of a complex 3D scattering specimen. Guided by insights from our phantom experiments, we first implemented the MSBP inverse-scattering solver using complex-valued field measurements acquired at multiple illumination angles. To avoid edge-related artifacts, we implemented sufficient volume padding equal to 100% of the imaging FOV.

**Fig. 5.**
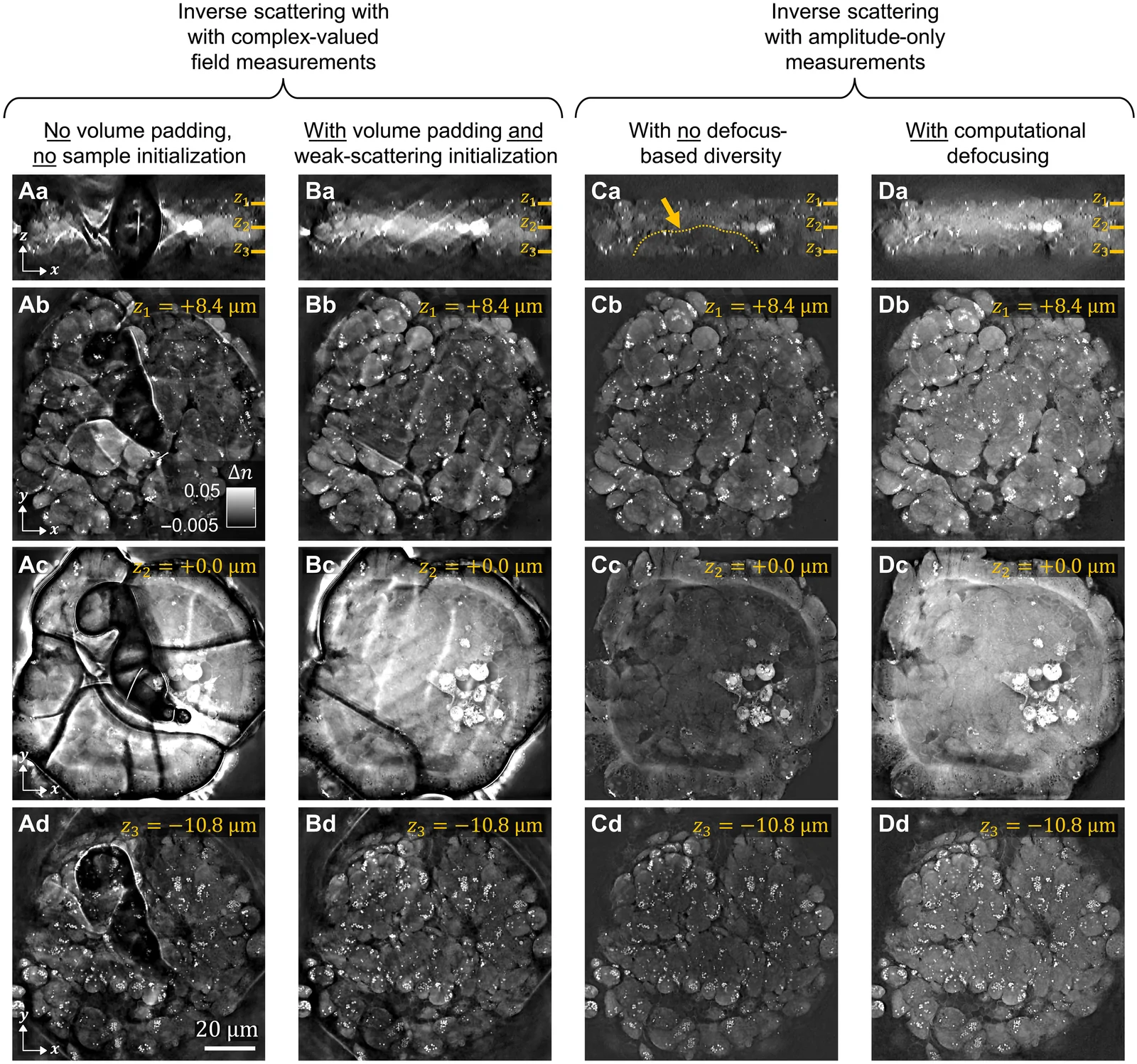
Comparison of MSBP-based inverse-scattering strategies for 3D RI reconstruction of an intestinal organoid sample. Axial cross sections are shown from the 3D RI reconstructions obtained using different inverse-solving strategies: (**Aa**) reconstruction using complex-valued field measurements with a uniform initial guess (Δn=0); (**Ba**) reconstruction using complex-valued field measurements with an initial guess derived from the weak-scattering approximation; (**Ca**) reconstruction using amplitude-only measurements without any initialization; and (**Da**) reconstruction using amplitude-only measurements with additional defocused measurements to introduce depth diversity. For each axial cross section, corresponding RI reconstructions at three lateral planes are also shown. Specifically, (**Ab**) to (**Ad**), (**Bb**) to (**Bd**), (**Cb**) to (**Cd**), and (**Db**) to (**Dd)** display lateral cross sections located at *z* = +8.4, +0.0, and −10.8 μm relative to the axial planes shown in (Aa), (Ba), (Ca), and (Da), respectively.

As a baseline, the inverse solver was first initialized with a uniform RI distribution equal to the background medium (Δn=0). As shown in Fig. 5 (Aa to Ad), this approach resulted in pronounced artifacts distributed throughout the organoid’s 3D reconstruction. This outcome mirrors the artifacts observed previously in Fig. 4 (Ca to Cd), where the phantom data underwent the same reconstruction conditions. This suggests that similar challenges arise in both synthetic and biological scattering scenarios when the reconstruction is poorly initialized and complex-valued field measurements are used in the inverse-scattering solver.

We then initialized the inverse solver with a 3D RI estimate derived from the weak-scattering approximation. In our earlier phantom experiments, this strategy of using complex-valued field measurements combined with a weak scattering–based initial guess proved effective in driving convergence to high-quality RI reconstructions. We observe a different outcome here. As shown in Fig. 5 (Ba to Bd), while the weak-scattering initialization does lead to improved results compared to the constant Δn=0 initial guess, the final 3D reconstruction still contains pronounced artifacts throughout the volume. This suggests fundamental differences in the scattering behavior of the organoid versus the phantom. Specifically, the organoid appears to exhibit significantly stronger multiple scattering, rendering the weak-scattering approximation insufficient as a warm start for the inverse solver. This interpretation is supported by a comparison of raw phase measurements (see fig. S2), where the organoid displays substantially more chaotic phase-wrapping than the phantom, indicating greater optical heterogeneity and a higher degree of multiple scattering.

We next implemented the inverse-scattering solver using amplitude-only measurements, without using an initial guess or volume padding. As shown in our earlier phantom experiments, this strategy demonstrated an unexpected robustness to reconstruction artifacts across a range of scattering strengths while still achieving reconstruction quality comparable to that of field-based measurements when combined with warm initialization and volume padding. Consistent with those findings, the results shown in Fig. 5 (Ca to Cd) exhibit similar behavior here. Unlike the field-based reconstructions in Fig. 5 (Aa to Ad) and Fig. 5 (Ba to Bd), no sharp spatial discontinuities or artifacts are observed. Structural features such as lipid droplets, apoptotic cells, and cellular boundaries can be clearly resolved.

However, one notable artifact is the presence of a low-contrast cavity region within the 3D organoid, which does not align with the expected physiological morphology. This region appears axially localized and lies beneath highly scattering structures such as apoptotic cells and lipid aggregates. We speculate that these features may scatter light away from the underlying cavity, misleading the inverse solver to converge to a solution containing a “shadow” beneath these high-RI structures (see dashed yellow line in Fig. 5Ca). Similar shadowing artifacts are observed in other scattering-based imaging modalities, such as optical coherence tomography (*62*).

To mitigate this effect, we introduced defocus diversity into the reconstruction process to increase axial sensitivity and stabilize reconstruction of features that may otherwise be occluded by high-RI structures. We do this by synthetically generating amplitude-only measurements from multiple defocused planes (*z* = −6, 0, and 6 μm relative to the imaging plane). This was achieved by computationally propagating the raw field measurements (described previously) and extracting only their amplitude components. As shown in Fig. 5 (Da to Dd), incorporating these defocused measurements significantly improves reconstruction quality by eliminating the shadow artifact and restoring contrast in the cavity region, thereby enabling clear visualization of the full organoid volume. This approach thus overcomes the major limitation of amplitude-only reconstruction without defocus diversity, which is that the sample’s phase information is disregarded. By leveraging the phase information indirectly encoded within defocused measurements, but not directly using the phase within the inverse solver to avoid phase-wrap artifacts, the inverse-scattering reconstruction process becomes more robust and yields more accurate reconstruction of internal structures.

Notably, this sample was immersed in PBS, a homogeneous aqueous medium. We observed similarly high-quality RI reconstruction when imaging scattering samples embedded in hydrogel (see fig. S4), highlighting the robustness of our method across different sample preparation conditions.

By combining insights from both the phantom and organoid experiments, we observe a consistent trend: Inputting complex-valued field data from multiple illumination angles into the inverse solver without warm initialization results in 3D reconstructions corrupted by sharp, discontinuous artifacts when imaging multiple-scattering samples. Initializing the solver with a 3D RI estimate based on the weak-scattering approximation helps stabilize the reconstruction and may be sufficient when the sample exhibits only mild multiple scattering, as seen with the phantom. However, for more strongly scattering samples like the organoid, this weak-scattering initialization is inadequate, and strong artifacts persist when using complex-field data in the solver. In contrast, amplitude-only measurements across multiple illumination angles proved to be a more robust strategy, producing artifact-free reconstructions in both phantoms and organoids without requiring any prior initialization. Nonetheless, even this approach may exhibit shadowing artifacts beneath highly scattering structures, reducing image clarity in deeper regions. To address this, defocus diversity from synthetically defocused amplitude-only measurements can be leveraged. This strategy of combining angular and defocus diversity significantly improved reconstruction quality and eliminated axial shadowing, enabling more uniform and high-clarity visualization of biological morphology throughout the organoid, as well as other biologically scattering samples (see figs. S5 and S6). Notably, in all cases when imaging these scattering samples, reconstruction with the traditional weak-scattering approximation fails (see fig. S7). With this methodology established, we now apply it to additional biologically relevant scattering samples to demonstrate its broader utility.

### Biological results

#### Mutant C. elegans strain with increased lipid synthesis

**Fig. 6.**
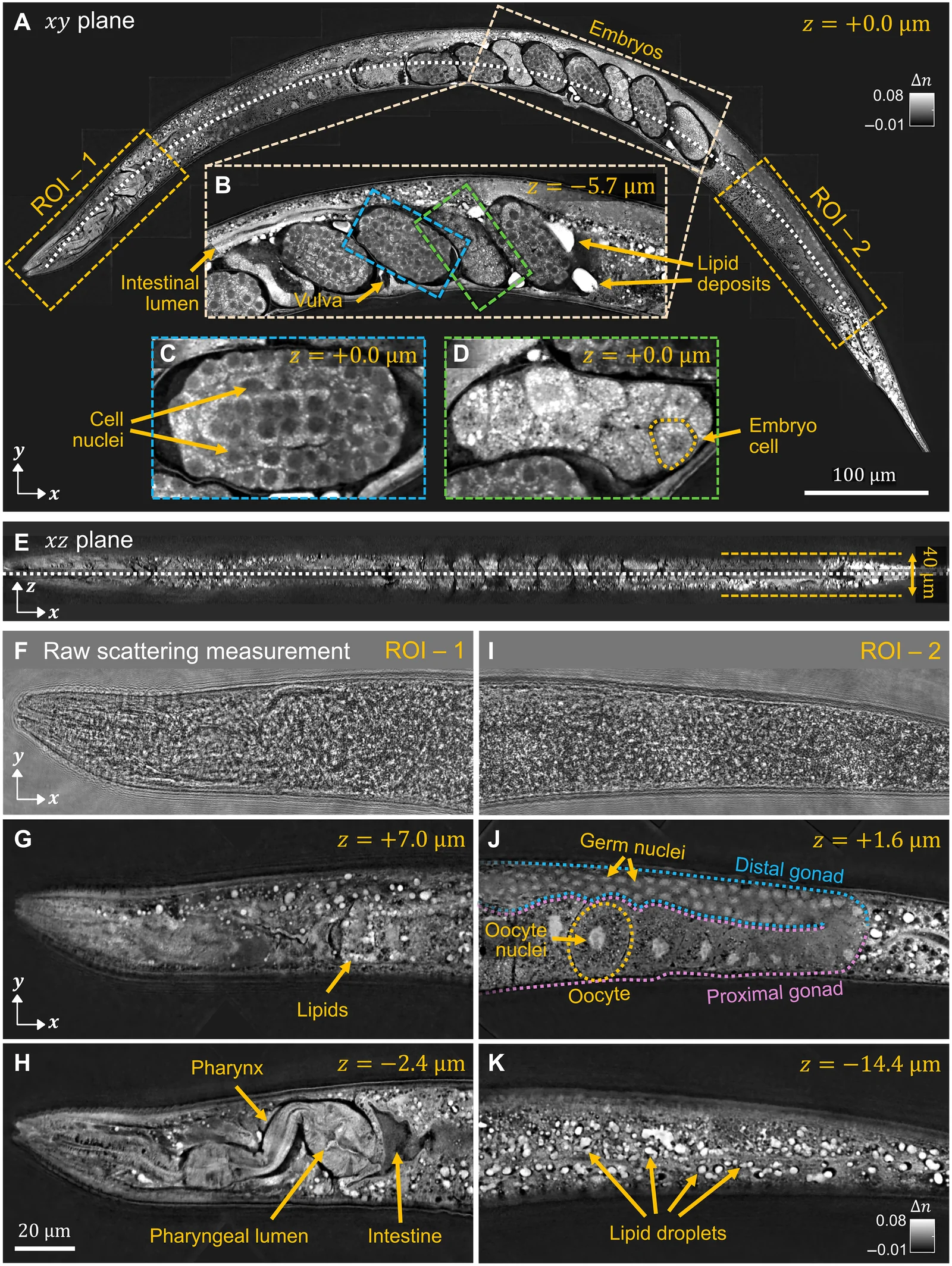
RI reconstruction results obtained in *daf-2(e1370)* mutant *C. elegans* samples, which is characterized by increased lipid accumulation. (**A**) Cross-sectional view is shown of the central *xy* plane, with various structures such as embryos, intestinal lumen, and vulva. (**B**) A zoomed-in image is shown of the region with embryos at different development stages. (**C** and **D**) Further magnified views of individual embryos show subcellular resolution, with embryonic cell boundaries and nuclei clearly visible. (**E**) Volumetric imaging capability is demonstrated by showing a curvature-corrected *xz* plane along the dashed white line in (A). (**F** to **H** and **I** to **K**) Raw scattering measurements (F and I) and corresponding 2D cross-sectional views are shown for two ROIs [(G), (H), (J), and (K)]. ROI-1 includes the head and pharynx region, and ROI-2 covers the tail region; both marked by boxes in the cross-sectional view in (A). The intestine, lipids, pharyngeal lumen, and pharynx in ROI-1, as well as the proximal and distal gonads, germ nuclei, oocytes, oocyte nuclei, distal gonad, and lipid droplets in ROI-2, are indicated by arrows.

We first demonstrate the capability of this inverse-scattering methodology by reconstructing the 3D RI of a *daf-2(e1370)* mutant *C. elegans* worm. The *daf-2(e1370)* mutant of *C. elegans* is a temperature-sensitive partial loss of function mutant with elevated lipid synthesis and storage compared to the wild type due to decreased insulin/insulin-like growth factor 1 signaling (*63*, *64*). The abundant internal lipid content in *daf-2(e1370)* mutants lead to increased optical scattering due to their different RI compared to the surrounding tissues (*65*). Figure 6A presents a lateral cross-sectional view of the central *xy* plane from the 3D image reconstructed via the methodology described previously. Despite the strong scattering caused by the high lipid concentration, the 3D structural features of the *C. elegans* were successfully recovered with remarkably high contrast. A magnified region is also shown, highlighting an area containing *C. elegans* embryos. Figure 6B presents a zoomed-in view of the worm’s embryo region, clearly revealing anatomical structures such as the intestinal lumen and vulva. Further magnified views in Fig. 6 (C and D) show individual embryos at subcellular resolution, with distinct cell boundaries and nuclei visible. Notably, the embryos display different cell counts that mark their respective developmental stages. This level of detail can be particularly valuable when studying embryonic development, especially when individual embryonic cells and nuclei can be volumetrically visualized within the intact worm. Combined with its capabilities for label-free morphological contrast, this imaging method thus provides a powerful tool for potentially identifying and analyzing different stages of cell development in vivo.

To highlight 3D structure, Fig. 6E shows a curvature-corrected axial cross-sectional *xz* plane along the dashed white line in Fig. 6A. Figure 6 (F to H and I to K) shows representative raw measurements and the corresponding reconstruction results from two different regions of interest (ROI) and imaging depths in the mutant *C. elegans*. ROI-1 corresponds to the anterior head region, while ROI-2 includes the tail region. In Fig. 6 (F and I), the raw intensity images captured under standard illumination show strongly scattered light, which obscures internal features because of the complex scattering within the sample. In contrast, the rich RI-based label-free contrast from the inverse scattering reconstructions in Fig. 6 (G and H and J and K) reveal detailed internal structures across different depths, including the pharynx, pharyngeal lumen, intestine, proximal gonad, germ nuclei, oocyte, oocyte nuclei, distal gonad, and lipid-rich regions. Specifically, lipid-rich regions appear as bright, spherical, vesicle-like structures due to their elevated RI and correspondingly increased optical scattering properties (*65*). Their morphology is consistent with previous studies, which showed that major fat stores in *C. elegans* are contained within distinct, spherical cellular compartments (*63*). Furthermore, high-quality volumetric visualization of intact cellular structures together with individual lipid droplets provides valuable insights into internal lipid organization without the need for additional labels or dyes. This capability is especially promising for studies on lipid metabolism, metabolic disorders, and the aging process (*66*, *67*).

Notably, these imaging results correspond to the reconstruction obtained using amplitude data with digital refocusing to axial positions of *z* = 3, 6, and 9 μm relative to the imaging plane. In contrast, reconstructions based on amplitude data without digital refocusing (i.e., raw amplitude data collected from one imaging plane) fail to resolve the internal structures (see fig. S5). Specifically, while the overall outline of the organism is still visible, fine internal features remain indistinct. We note that 3D reconstruction of a wild-type *C. elegans*, which does not have elevated lipid synthesis and is thus less optically scattering, does not seem to be heavily affected by whether digital refocusing is used (see fig. S6). This result demonstrates that incorporating digitally refocused data is most effective for samples with complex internal scattering.

#### Intestinal organoid

Next, we performed MSBP-based inverse scattering of an intestinal organoid larger than the one shown previously in Fig. 5. Organoids are 3D cellular structures derived from stem cells that closely mimic the architecture and function of the tissue of origin. These models have emerged as powerful tools for studying tissue development, host-microbiome interactions, and drug-induced toxicity, with more physiological relevance than traditional 2D cultures (*68*, *69*). However, imaging organoids presents challenges due to their multicellular organization and size, which leads to substantial light scattering. Traditional imaging systems such as bright-field and epifluorescence microscopy often lack sufficient optical sectioning and are prone to high background noise, making them suboptimal for volumetrically resolving internal features in 3D organoids (*70*). While confocal and light-sheet microscopies enable optical sectioning for 3D organoid imaging, they typically rely on fluorescent labeling, which is subject to photobleaching and can disrupt organoid function (*71*, *72*).

To address these limitations, we evaluated the performance of our inverse-scattering framework for label-free 3D RI imaging of an intestinal organoid sample with a physical thickness of approximately 180 μm. In our previous experiments, we used a high numerical aperture (NA) 1.42 oil immersion lens to achieve high-resolution imaging. However, the short working distance of such lenses restricted the imaging depth, limiting their applicability for the thicker organoid specimens. Therefore, we adopted a 0.8-NA water-dipping objective for this study, which offered a longer working distance that could accommodate the relatively thick size of the organoids.

**Fig. 7.**
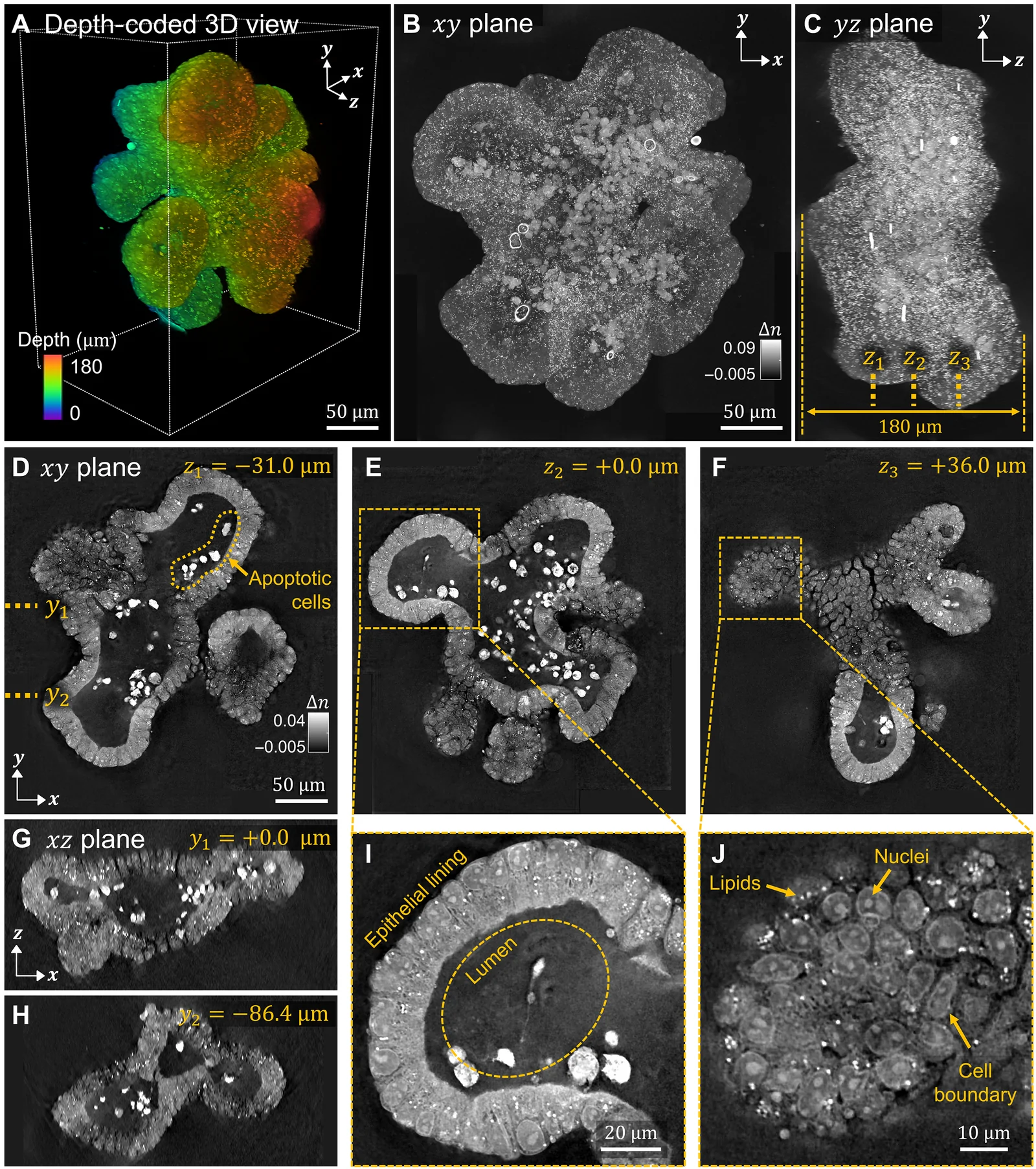
Inverse-scattering reconstruction results for intestinal organoid samples. (**A**) Depth-coded tomographic view of the reconstructed 3D RI volume. (**B** and **C**) Maximum intensity projections are shown along the *z* axis and *x* axis, respectively. (**D** to **F**) Lateral *xy* plane cross-sectional views are shown at different *z* planes within the reconstruction volume, corresponding to the dashed lines indicated in (C). (**G** and **H**) Axial *xz* plane cross-sectional views at different *y* planes of the reconstruction volume, corresponding to the dashed lines indicated in (D). (**I** and **J**) Zoomed-in *xy* plane lateral views of the regions outlined by dashed boxes in (E) and (F). Epithelial lining, lumen, lipids, nuclei, and cell boundaries are clearly visible in the images and indicated by arrows.

Figure 7 presents the 3D RI reconstruction results of the intestinal organoid. Figure 7A shows a 3D rendered, depth-coded tomographic view, while Fig. 7 (B and C) displays maximum intensity projections along the *z* and *x* axes of the reconstructed volume, respectively. Figure 7 (D to F) shows lateral *xy*-plane cross-sectional views at different *z* planes, corresponding to the dashed lines indicated in Fig. 7C. Figure 7 (G and H) shows axial *xz*-plane cross-sectional views at different *y* positions, corresponding to the dashed lines in Fig. 7D. Together, these views volumetrically reveal important morphological features of the organoid, such as the crypts, lumen, and epithelial lining. In addition, Fig. 7 (I and J) illustrates zoomed-in views that reveal densely packed cell clusters along the epithelial lining, allowing detailed subcellular visualization of cell boundaries, nuclei, lipids, and other intracellular components. Together, the overall morphology and the fine subcellular details revealed in the RI reconstruction offer valuable insights into the sample’s spatial organization and internal architecture.

This imaging capability can have important applications. For example, intestinal physiology dictates a continuous cell turnover in the intestinal epithelium, with the cells at the villus tip undergoing anoikis and shedding into the lumen (*73*). The presence of bright signal (corresponding to high RI) from cell bodies within the organoid lumen is suggestive of apoptotic cells with elevated lipid content. Previous studies have consistently demonstrated that lipid accumulation occurs in epithelial cells during apoptosis (*74*). This phenomenon can serve as an indication for apoptotic cell detection using magnetic resonance spectroscopy (*75*). The increase in intracellular lipid content is attributed to the suppression of mitochondrial fatty acid β oxidation, which diminishes fatty acid catabolism and enhances lipid biosynthesis during the apoptotic process (*76*). Because RI-based contrast is highly sensitive to lipid content, image reconstruction via inverse scattering may be especially useful for the detection of apoptotic cells in certain tissues.

As before, the 3D RI reconstruction shown in Fig. 7 was performed using amplitude-only data with computational defocusing (*z* = 36, 40, and 44 μm relative to the imaging plane). Without incorporating defocused measurements, sharp reconstruction artifacts appeared both around the periphery and within the organoids (see fig. S6). These artifacts were more severe than the slowly varying shadow artifacts observed previously in Fig. 5 when 3D RI in a smaller intestinal organoid was reconstructed without using computational defocusing. We speculate that this is due to the specific internal structure of the intestinal organoids. Specifically, the interface between the epithelial lining and the lumen in this larger organoid constitutes a sharper RI transition that may introduce refractive effects not accurately modeled by the MSBP framework. This model mismatch may thus lead to artifacts around interfaces with abrupt RI change. By including defocused data that shift the imaging focus away from the central lumen toward regions where the RI mismatch is less severe, these effects were observed to be substantially reduced although not always eliminated (see figs. S8 and S9).

#### Zebrafish embryo

Last, we show 3D RI reconstruction of a 24–hour postfertilization (hpf) zebrafish embryo, which is a widely used model organism in developmental biology and biomedical research. As a representative multicellular system, it provides invaluable insights into embryogenesis and organ development. However, imaging the zebrafish embryo at subcellular resolution, can be challenging due to its complex multicellular architecture, which gives rise to substantial multiple light scattering. Accurately visualizing and quantifying its internal structures in 3D is essential for understanding developmental processes, but conventional imaging techniques often fall short because of scattering-induced degradation in resolution and contrast.

To address these limitations, we used an MSBP-based inverse-scattering method to perform label-free, high-resolution 3D RI reconstruction of the zebrafish embryo. As in our imaging experiments with intestinal organoids, we used the 0.8-NA water-dipping objective lens for longer working distance. We reconstructed the sample’s 3D RI using refocused datasets (*z* = −8, 0, and 8 μm relative to the imaging plane), which allowed for better compensation of scattering and improved convergence during the iterative reconstruction process. This approach outperformed conventional amplitude-only MSBP reconstructions, particularly in deeper or highly scattering regions of the sample.

**Fig. 8.**
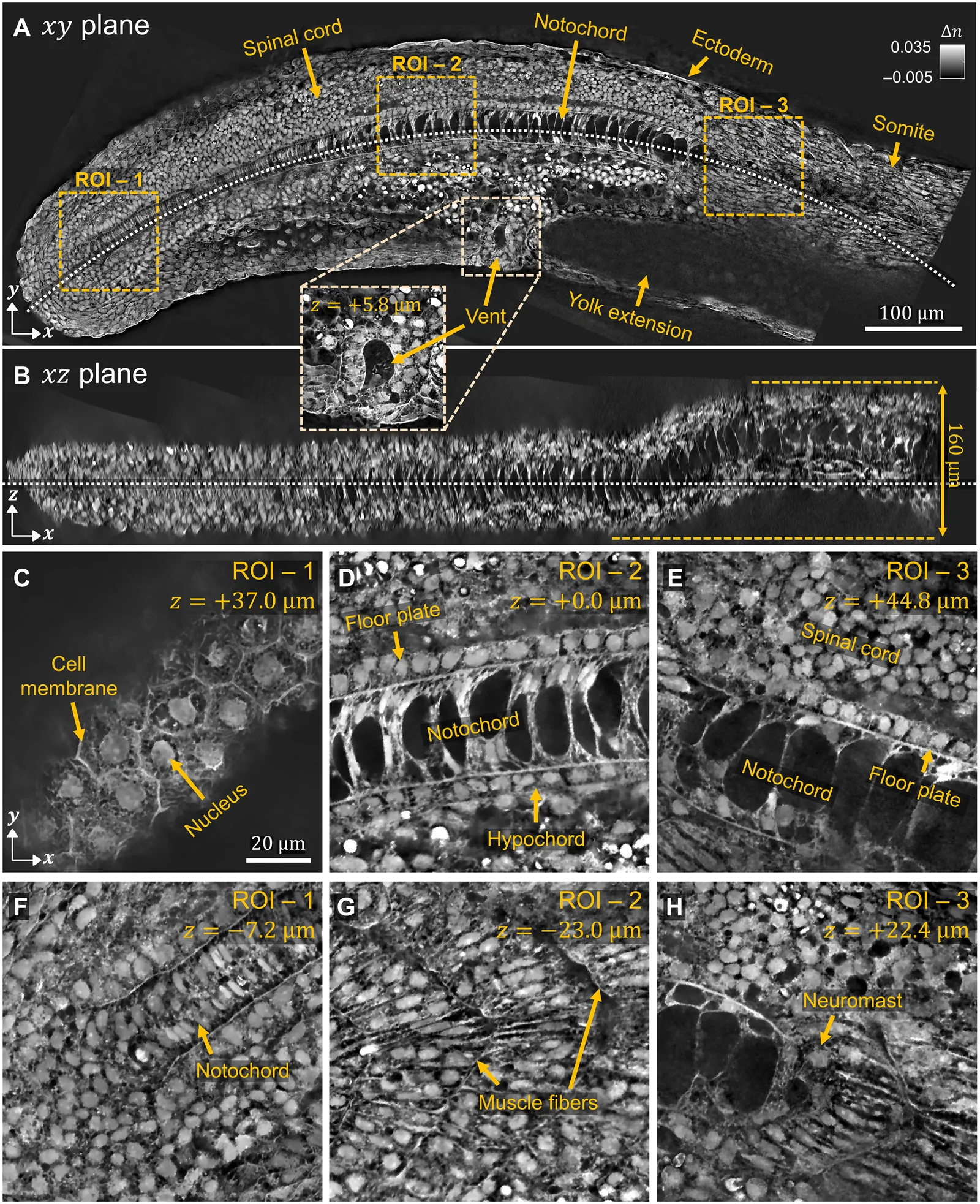
MSBP-based inverse-scattering reconstruction of a 24-hpf zebrafish embryo, with large-scale anatomical landmarks highlighted. (**A**) Cross-sectional view of the central *xy* plane, along with (**B**) a curvature-corrected *xz* plane along the dashed white line in (A), reveal the 3D morphology based on RI contrast. (**C**) and (**F**), (**D**) and (**G**), and (**E**) and (**H**) show the zoomed-in *xy* views at different depths within three ROIs—ROI-1, ROI-2, and ROI-3, respectively. These zoomed-in views reveal subcellular-level resolution, enabling clear identification of fine structural features.

Figure 8 presents detailed views from the 3D RI reconstruction, which may provide valuable insights into the spatial organization of cells and the morphogenetic processes of the embryo. For example, Fig. 8A presents a central *xy*-plane slice of the reconstructed 3D RI volume, where internal anatomical features such as the spinal cord, notochord, vent, and somites are clearly visualized with subcellular resolution. The epithelial nature of the digestive track is readily evident, and a zoomed-in view clearly visualizes the vent, which is the external opening of the cloaca. Figure 8B shows a curvature-corrected *xz* plane along the dashed white line in Fig. 8A, clearly revealing the distribution of notochord from an axial perspective. Figure 8 (C to H) provides magnified views of different ROIs indicated by the dashed boxes in Fig. 8A. For example, Fig. 8C shows the surface of the sample, where the nuclei and cell membrane at regions of cell-cell contact between neighboring periderm cells are clearly evident. Intracellular heterogeneity, possibly due to cytoskeletal structures, are also visible. Figure 8F shows that the nascent notochord can be visualized lower in depth, closer to the midline of the sample. Moving anteriorly along the sample, Fig. 8D shows a more developed notochord, within which prominent vacuoles are clearly distinguishable. The hypochord is visible just ventral to the notochord, and the floor plate of the spinal cord is readily discernible. Imaging deeper into the sample (Fig. 8G), somites are clearly visible and individual muscle fibers can be distinguished. Further anterior, Fig. 8E shows that cells within the notochord are highly vacuolated; thus, in this specific sample, the maturation of the notochord can be visualized. Lower in depth, Fig. 8H shows that cells of a neuromast can be visualized situated between the muscle and surface ectoderm.

The region indicated by the arrow in Fig. 8A corresponds to the yolk extension, which could not be successfully reconstructed likely because of its excessive scattering and absorption properties. Figure S9 shows that the scattering profile of the yolk region consists essentially of chaotic speckle, with speckle patterns from adjacent illumination angles appearing almost completely uncorrelated. Effectively performing inverse scattering in such highly heterogeneous regions will be an important direction for future research.

## DISCUSSION

Our motivation for this work stems from the long-standing fundamental challenge in optical imaging regarding its inability to see deep into tissue due to strong biological scattering. Hardware-based approaches have been developed to physically resist or reverse effects of scattering but often face challenges such as limited degrees of freedom for scatter correction, reliance on guide stars, the need for labeling, and high system costs (*77*–*79*). In contrast, computational inverse-scattering methods aim to digitally undo scattering effects. In this approach, the primary challenges lie in the accuracy of the physics-based scattering model and the efficiency of the inversion algorithms. As computational power improves, these challenges become more manageable. However, a fundamental issue has remained largely unaddressed: Most multiple-scattering inverse solvers are inherently nonconvex and can produce different converged solutions depending on the specific implementation strategy, even when using the same underlying physical model to reconstruct from a single dataset. In complex scattering scenarios, these solutions may fail to reflect the sample’s true physiological structure.

In this work, we aimed to develop a methodology for MSBP-based inverse scattering that enables robust and accurate 3D RI reconstruction in optically thick, scattering samples. Recall that a sample’s 3D RI distribution can itself be interpreted as a volumetric image that enables label-free and quantitative visualization of the sample’s morphology, providing detailed insights into cellular structures, multicellular organization, and tissue architecture. RI imaging enables these features to be visualized with high contrast without the need for exogenous labels or perturbative agents. Furthermore, aside from serving as a source of imaging contrast, RI is also the fundamental physical parameter governing a sample’s scattering behavior. Thus, accurately reconstructing RI not only enables morphological imaging but may also have valuable downstream implications for scatter correction in other techniques, such as wavefront shaping (*80*, *81*), adaptive optics (*82*, *83*), super-resolution (*84*, *85*), and 3D virtual labeling (*86*, *87*).

To formulate an effective inverse-scattering strategy, we first started with 3D scattering microphantoms, which provided ground-truth structures for initial validation and benchmarking. This enabled us to identify implementation strategies that produce robust and accurate RI maps. We then extended and refined these approaches using intestinal organoids as examples of biologically relevant and more complex scattering samples. All implementation strategies were evaluated across consistent datasets, thereby controlling for acquisition conditions, sample content, and signal-to-noise ratio. This allowed for a fair, side-by-side comparison of implementation strategies under consistent conditions.

We found that using amplitude-only measurements in the MSBP inverse solver in Eq. 4 generally produced more stable and physiologically realistic 3D RI reconstructions compared to using complex-valued field measurements. This result is unexpected given that field measurements contain both amplitude and phase information, which intuitively should provide more data for reconstruction. We speculate that the phase component introduces ambiguities due to phase wrapping, which can mislead the nonconvex inverse solver and cause it to converge to incorrect solutions. This effect was consistently observed across datasets collected of scattering biological samples (e.g., intestinal organoid in Fig. 5, and mutant *C. elegans* in fig. S5), where reconstructions from field data exhibited strong artifacts that worsened as the level of phase wrapping increased in the raw field measurements. We note that for moderately scattering samples without severe phase wrapping (e.g., scattering cube phantoms; Fig. 4 and fig. S2), field-based MSBP achieves high-quality RI reconstructions when appropriate initialization and volume padding were used. In such regimes, a more careful investigation of initialization and padding in complex-field MSBP may be worthwhile for future work.

Phase information encoded within complex-valued field measurements proved indirectly valuable for amplitude-based MSBP. By numerically defocusing the measured fields and then incorporating their amplitude components into the inverse solver, we were able to resolve artifacts that persisted when using only angular amplitude measurements. This indicates that combining angled illumination and sample defocus measurements constrain the MSBP inverse problem more effectively than illumination angles alone, enabling convergence toward more physiologically accurate solutions (see fig. S10). This is consistent with the broader principle that diverse measurements are critical to robustly navigate the solution space of nonconvex inverse problems (*88*–*90*). Notably, for weakly scattering samples, prior works have shown that amplitude-only measurements acquired under different illumination angles and sample defocus position directly probe specific regions of the sample’s 3D RI spectrum (*23*, *54*). This provides a useful framework for linking reconstruction quality to the measurement strategy. No comparable framework exists yet for multiple-scattering samples, presenting an important and exciting direction for future work.

Several strategies can be explored to further improve the methodology introduced in this work. For example, our current off-axis interferometric system captures complex-valued fields to achieve computational defocusing but incurs drawbacks of complex system design, large physical footprint, and sensitivity to optical path fluctuations. A promising alternative is a noninterferometric system combining angular illumination with high-speed remote focusing to directly acquire amplitude images at multiple sample planes. Although there are important practical challenges associated with physical defocusing [e.g., independent noise at each physical measurement plane, partial-coherence (*91*), and optical aberration effects (*92*), aliasing artifacts that accumulate for large-distance digital defocus (*93*), etc.], comparable reconstruction quality should be achievable with a well-designed but simpler, more stable, and more compact system.

Another avenue for future work is optimizing the refocusing process by determining the most informative defocus planes and the number of defocus levels needed to optimally enhance reconstruction quality. Furthermore, although our datasets in this work composed of scattering measurements resulting from angular plane-wave illumination applied one direction at a time, future work could examine how the choice of illumination angles or the simultaneous use of multiple angles influences reconstruction results (*94*, *95*). Another possibility is to design nonplanar illumination wavefronts or structured patterns that effectively constrain the inverse problem and improve robustness (*96*, *97*). In addition, while total variation was used as the regularizer in this work, more advanced forms of regularization could be developed. For example, as realistic biomimicking scattering microphantoms become more accessible (*98*, *99*), it may be possible to train neural networks that map scattering measurements to RI distributions for specific types of samples (*100*, *101*). These networks could then serve as learned regularizers that provide more targeted constraints, improving the convergence, and accuracy, and speed of the inverse-scattering process for particular biological systems. Last, other studies have also explored using neural networks to accelerate the inverse-scattering process (*102*, *103*). Incorporating such approaches could offer valuable improvements in this context as well.

Last, all of the inverse-scattering results presented in this work are based on the MSBP model, which is a relatively simple framework for light scattering that assumes forward-only, paraxial propagation. While the practical limitations of this assumption are not yet fully established [e.g., MSBP has been shown to provide quantitatively accurate reconstruction even with nonparaxial imaging (*34*)], it is reasonable to expect challenges as samples become increasingly scattering beyond the regime where MSBP provides accurate modeling. These challenges may include reconstruction artifacts at interfaces with sharp RI transitions (such as with certain organoids as shown in fig. S9), as well as failures to reconstruct in regions exhibiting highly chaotic scattering (such as with the yolk region in the zebrafish embryo in Fig. 8). Scattering models more advanced than MSBP have been proposed and may be better suited for such challenging cases (*39*, *40*, *46*, *104*, *105*). However, their added complexity makes rigorous evaluation even more critical as they may exhibit different convergence behaviors and require distinct implementation strategies. Thus, if properly optimized, these models may extend the reach of inverse-scattering methods to a broader class of samples, including to those where our current approach struggles. We hope this work motivates future efforts to systematically refine and adapt such inverse-scattering techniques for deep-tissue imaging in increasingly thick, multicellular samples, paving the way toward robust, large-scale volumetric biological imaging.

## MATERIALS AND METHODS

### Optical system

Our experimental system consists of a Mach-Zehnder off-axis interferometer configured to perform angular illumination scans, as illustrated in Fig. 2A. Light from a red laser (Thorlabs CPS635R, 635-nm center wavelength) is delivered via a single-mode fiber into the system after being collimated using lens L1 (Newport MVC-10X Air, NA 0.25). The collimated beam is then split into a sample arm and a reference arm using a beam splitter. In the sample arm, the beam is directed onto a dual-axis galvanometric mirror that steers the beam’s angle. A 4f optical relay composed of lens L2 and a condenser objective lens images the galvo mirror plane onto the sample plane, allowing precise control of the illumination angle. Light scattered from the sample is collected by a second 4f system, consisting of an imaging objective lens and lens L3, and directed toward the detection path. To maintain symmetry and minimize aberrations, we use identical optics in both arms: Lenses L2 and L3 are matched (Thorlabs ACT508-200-A) as are the condenser and imaging objectives. In the case when optimizing for high-resolution, oil-immersion objective lenses were used (Nikon CFI Plan Apochromat Lambda D 60× oil, NA 1.42). When optimizing for long working distance to image thicker samples, water-dipping objective lenses were used (Nikon N16XLWD-PF 16× water, NA 0.8, WD 3.0 mm). Last, the sample and reference beams are recombined at a second beam splitter, forming a spatially modulated interferogram that is captured by a complementary metal-oxide semiconductor camera (ToupTek IUA20000KMA).

### Data acquisition and preprocessing

Interferograms are first acquired at various illumination angles for both the sample and an empty region without the sample (i.e., background). These angles are controlled by steering the two-axis galvo mirror. From each interferogram, complex-valued field images containing both amplitude and phase are reconstructed by filtering the sample’s spectrum in Fourier space, following standard procedures for quantitative phase imaging via off-axis holography. This yields a set of amplitude and phase measurements across different illumination angles for both the sample and background. Background correction is performed by complex division of the sample and background fields, corresponding to dividing the amplitude and subtracting the phase terms. This step compensates for system-induced phase variations unrelated to the sample. Figure 2 illustrates this data acquisition pipeline using a scattering microphantom.

Depending on the reconstruction strategy, either the background-corrected complex field measurements or only their amplitude components were used in the MSBP-based inverse-scattering solver described in Eq. 4. The complex field data can also be combined with the Rytov approximation and the Fourier diffraction theorem to analytically reconstruct an initial RI distribution for the sample under the weak-scattering assumption. This initial estimate can serve as a warm start for the MSBP solver to help stabilize and accurately guide the convergence process, as demonstrated when using field measurements in the inverse solver (see Fig. 4).

Last, complex field measurements can be digitally propagated to synthetically generate defocus diversity without having to physically capture defocused measurements. This was shown earlier to reduce reconstruction artifacts when using amplitude-only data in the MSBP solver (see Figs. 4 and 5). To perform digital propagation, a Fourier transform is first applied to the measured field. The resulting spectrum is then multiplied by the free-space transfer function from Eq. 1, followed by an inverse Fourier transform to reconstruct the propagated field. Technical details on data acquisition and computational reconstruction for the various samples are provided in table S1.

### MSBP inverse scattering for complex-valued field and amplitude-only measurements

Here, we provide additional details about the mathematics that compose the MSBP inverse-scattering model. As discussed earlier, MSBP models light scattering by representing the 3D sample volume as a series of infinitesimally thin 2D layers, each acting as a phase-modulating element. In Eqs. 2 and 3, we mathematically defined the wave propagation and phase modulation steps that determine how the field at the mth layer, $E_{m}$, evolved on the basis of the RI at that layer and the field from the preceding layer, $E_{m-1}$. By starting with the field at the 0th sample layer (i.e., the illumination field), repeatedly applying Eqs. 2 and 3 propagates the incident field layer-by-layer through the complete sample. The sample’s cumulative scattering effects are ultimately expressed in the output field at the final layer, i.e., $E_{M}$ for a M-layer sample.

In practice, however, $E_{M}$ does not correspond directly to the field at the imaging plane, which is typically focused to a specific depth within the sample. Furthermore, to physically reach the imaging plane, $E_{M}$ must propagate through the detection optics and must thus be subject to the finite NA of the objective lens, which limits spatial resolution. As a result, the field at the imaging plane is a back-propagated and resolution-limited version of $E_{M}$ and can thus be expressed as

$$E_{\text{im}}^{\left(\ell \right)}\left(\vec{r}\right)=\left[p⊗h_{-\hat{z}}⊗E_{M}^{\left(\ell \right)}\right]\left(\vec{r}\right)$$ (5)

Recall that $\vec{r}$ denotes the 2D transverse (x,y) spatial coordinates, ℓ denotes the index of measurement, and ⊗ represents the convolution operator. The term $h_{-\hat{z}}$ is the optical back-propagation kernel corresponding to the distance $\hat{z}$ between the final sample layer and the conjugate imaging plane within the sample. Last, p represents the system’s point spread function, which is determined by the NA of the detection optics and defines the system’s spatial resolution.

If the system directly measures complex-valued fields, the MSBP forward model describing the raw measurement for some known 3D RI distribution is simply

$$G_{\text{field}}^{\left(\ell \right)}\left\{n\left({\vec{r}}_{3D}\right)\right\}=E_{\text{im}}^{\left(\ell \right)}\left(\vec{r}\right)$$ (6)

For mathematical clarity, we use slightly different notation here compared to before by introducing the term $G_{\text{field}}^{\left(\ell \right)}$ as the MSBP forward model for complex-valued field measurements. Recall that ${\vec{r}}_{3D}$ denotes the 3D spatial coordinate (x,y, and m), where m indexes the axial layers of the sample’s 3D RI distribution, i.e., $n\left({\vec{r}}_{3D}\right)=n_{m}\left(\vec{r}\right)$ for m=1,2,…M for an M-layer sample. Note that to measure complex-valued fields, some version of quantitative phase imaging must be used. In this work, we computationally extract the complex-valued field term from an off-axis interferogram.

The MSBP forward model for amplitude-only measurements can be simply expressed as

$$G_{\text{amp}}^{\left(\ell \right)}\left\{n\left({\vec{r}}_{3D}\right)\right\}=|E_{\text{im}}^{\left(\ell \right)}\left(\vec{r}\right)|=|G_{\text{field}}^{\left(\ell \right)}\left\{n\left({\vec{r}}_{3D}\right)\right\}|$$ (7)

Thus, for a known 3D sample RI distribution, the MSBP forward models in Eqs. 6 and 7 predicts either the complex-valued field or amplitude-only scattering measurements that the imaging system would acquire.

In practice, the goal of inverse scattering is to infer the sample’s 3D RI from a set of scattering measurements. As described earlier in Eq. 4, we perform this inversion by iteratively minimizing the difference between experimentally measurements and model predictions. For mathematical clarity, we reexpress Eq. 4 below

$$\hat{n}\left({\vec{r}}_{3D}\right)=\underset{n\left({\vec{r}}_{3D}\right)}{\text{argmin}}\;\left[\frac{1}{2L}\sum_{\ell =1}^{L}D^{\left(\ell \right)}+\tau \cdot R\right]\left\{n\left({\vec{r}}_{3D}\right)\right\}$$ (8)

Recall from Eq. 4 that R denotes the regularization operator that stabilizes the iterative search. The parameter τ sets the strength of regularization and is empirically tuned. Here, we introduce the term D to consolidate the least-squares term in Eq. 4 based on whether complex-valued field or amplitude-only measurements are being used

Least-squares term for field measurements

$$D_{\text{field}}^{\left(\ell \right)}\left\{n\left({\vec{r}}_{3D}\right)\right\}={\left\|y_{\text{field}}^{\left(\ell \right)}\left(\vec{r}\right)-G_{\text{field}}^{\left(\ell \right)}\left\{n\left({\vec{r}}_{3D}\right)\right\}\right\|}_{L2}^{2}$$ (9)

Least-squares term for amplitude measurements

$$D_{\text{amp}}^{\left(\ell \right)}\left\{n\left({\vec{r}}_{3D}\right)\right\}={\left\|y_{\text{amp}}^{\left(\ell \right)}\left(\vec{r}\right)-G_{\text{amp}}^{\left(\ell \right)}\left\{n\left({\vec{r}}_{3D}\right)\right\}\right\|}_{L2}^{2}$$ (10)

Here, we use the terms $y_{\text{field}}^{\left(\ell \right)}$ and $y_{\text{amp}}^{\left(\ell \right)}$ to differentiate between complex-valued field versus amplitude-only measurements.

The 3D RI reconstructions shown in this work are obtained by solving the minimization problem in Eq. 8 using accelerated stochastic gradient descent. Other optimization algorithms could also be used and would give comparable results (see fig. S11). All strategies require computing the gradient of the least-squares term D, either through automatic differentiation or manual derivation. To better understand how the measurement type influences the optimization, we analytically derive the gradient expressions for both the complex field and amplitude-only measurements (see note S1), as summarized below

Gradient for field measurements

$${\left[\nabla D_{\text{field}}^{\left(\ell \right)}\right]}^{H}={\left[\frac{\text{∂}}{\text{∂}n}G_{\text{field}}^{\left(\ell \right)}\right]}^{H}\left[G_{\text{field}}^{\left(G\right)}-y_{\text{field}}^{\left(\ell \right)}\right]$$ (11)

Gradient for amplitude measurements

$${\left[\nabla D_{\text{amp}}^{\left(\ell \right)}\right]}^{H}={\left[\frac{\text{∂}}{\text{∂}n}G_{\text{field}}^{\left(\ell \right)}\right]}^{H}\left[G_{\text{field}}^{\left(\ell \right)}-y_{\text{amp}}^{\left(\ell \right)}\;e^{j∠G_{\text{field}}^{\left(\ell \right)}}\right]$$ (12)

Here, ∇ denotes the gradient operator and H represents Hermitian transpose. The gradient expressions above for complex-valued field and amplitude-only measurements are nearly identical and can both be expressed in terms of the MSBP forward model for complex-valued fields. The key distinction between Eqs. 11 and 12 is that the amplitude-only measurement $y_{\text{amp}}^{\left(\ell \right)}$ in Eq. 12 is paired with the phase of the predicted field $G_{\text{field}}^{\left(\ell \right)}$. This combined term $y_{\text{amp}}^{\left(\ell \right)}\;e^{j∠G_{\text{field}}^{\left(\ell \right)}}$ is effectively a synthetic complex-valued measurement that mimics the $y_{\text{field}}^{\left(\ell \right)}$ term in Eq. 11. A detailed derivation of these gradients, along with a step-by-step guide for implementing the inverse-scattering framework, is provided in the Supplementary Materials.

Notably, while the mathematical difference between the two gradient expressions appears subtle, it leads to substantial differences in convergence behavior and accuracy, as shown in the 3D reconstructions in Figs. 4 and 5. Future work could investigate the theoretical implications of this difference and explore whether modified formulations might further improve reconstruction accuracy and stability.

### Quantitative characterization and selection of step size and regularization parameters

To iteratively solve the minimization problem in Eq. 8, users must select values for the step size and the regularization parameter τ. We found that these choices significantly influence the reconstructed RI values, affecting the quantitative accuracy of the RI reconstructions. To empirically determine suitable parameters, we collected angular scattering measurements from polystyrene, polymethyl methacrylate, and silica microspheres (BangLabs) of varying diameters, immersed in index-matching oils (Cargille). We then reconstructed the RI maps of the microspheres using a range of step size and regularization values.

Because the true RI contrasts of these samples were known, we were able to evaluate the quantitative accuracy of each reconstruction and identify parameter combinations that yielded the most accurate results. Given the nonconvex nature of the inverse problem, multiple parameter pairs achieved high accuracy on a single sample. To increase robustness, we then selected the parameter combination that consistently produced accurate reconstructions across all tested samples. See fig. S1 to see more details about this selection process.

Last, we validated this parameter set using the 40-μm scattering phantom, confirming its generalizability. These parameters were then used for all subsequent reconstructions of biological samples.

### Sample preparation for imaging experiments

#### Microphantom samples

The microphantoms were fabricated via the two-photon polymerization lithography (*106*) using the Photonic Professional GT2 system (Nanoscribe GmbH & Co. KG, Karlsruhe, Germany), equipped with 1.4 NA 63× microscope objective, piezo stage for vertical positioning of the sample, and dual-axis galvo for lateral scanning. The fabrication process leveraged 3D-printing principles, where a tightly focused femtosecond laser was scanned across liquid resin (IP-DIP2, Nanoscribe GmbH & Co. KG, Karlsruhe, Germany). This induced two-photon absorption, polymerizing the resin along the laser’s path. This technique enabled fabrication of microstructures, with high 200-nm lateral resolution, directly on standard #1.5H coverslips. Crucially, the local energy dose could be precisely controlled to dictate the degree of monomer cross-linking, which in turn was proportional to the resulting RI (*99*, *107*). Thus, microstructures were fabricated with carefully calibrated 3D spatial profiles and RI. Moreover, the fabrication process was performed in dip-in configuration (*108*), where the microphantoms were cured layer-by-layer as the substrate moved away from the objective lens. This strategy prevented the polymerization beam from being degraded by previously cured layers, thereby ensuring the high fidelity of the structures throughout their entire height.

After patterning, unpolymerized resin was removed by a 12-min bath in propylene glycol monomethyl ether acetate (Sigma-Aldrich, catalog: 484431), followed by a 1-min bath in N7100 (Novec 7100 Engineered Fluid; Sigma-Aldrich, catalog: SHH0002), and then air-drying. Drawing from previous work (*61*, *109*), microphantom designs featured a cell phantom embedded within a pseudo-random distribution of rods. These rods alternated their orientation in successive layers, with each rod measuring 0.5 μm in width and 1.8 μm in height. The lateral spacing between rods in each layer was randomized from 0.7 to 3 μm, and layers were stacked vertically every 1.4 μm. The polymer occupied approximately 25% of the total volume (fill-factor), with scattering strength determined by the size of the scattering region (40 to 100 μm). During imaging experiments, the microphantoms were immersed in index-matching oil (Cargille, RI = 1.512), which also served as the immersion medium for the oil-immersion objective lens. The objective lens was directly immersed into the same oil, allowing imaging without an additional coverslip on top of the sample.

#### C. elegans worms

Wild-type and *daf-2(e1370)* mutant *C. elegans* were obtained from the *Caenorhabditis* Genetics Center (CGC). To prepare for imaging experiments, 5 to 10 wild-type and mutant worms were transferred onto a large coverslip in 50 μl of PBS. Excess PBS was carefully removed using a 10-μl pipette tip to avoid disturbing the specimens. The worms were fixed with 50 μl of 4% paraformaldehyde in 1× PBS for 2 min, after which the fixative was removed. A graded ethanol rehydration series was then performed: Fifty microliters of 95, 75, 50, and 25% ethanol was sequentially applied, each for 2 min, with the ethanol removed between steps. To complete rehydration, the animals were incubated in 50 μl of PBS for 2 min. After removing the PBS, the animals were resuspended in 40 μl of double-distilled water and remounted on a coverslip.

#### Intestinal organoids

Intestinal tissue (colon) used for establishing the organoid culture was purchased from Marshall BioResources, which belonged to a single female research-bred Beagle (∼2 years and 7 months old, 8.4 kg). Culture and maintenance of organoids were performed as per published protocols (*110*, *111*). To prepare samples for imaging, organoids were suspended in Matrigel, seeded as domes into 24-well plates and cultured for 5 days. On day 5, organoids were recovered from the Matrigel domes by replacing the culture medium in the wells with 0.5 ml of cold Cell Recovery Solution (Corning Inc.). The domes were triturated gently several times, and the contents of the wells were transferred to a 15-ml conical tube. The tube was then filled with 10 ml of ice-cold PBS (1×) and centrifuged at 100*g* for 3 min at 4°C. The supernatant, containing most of the Matrigel, was aspirated, and the pellet was kept on ice.

For preparing the sample in Fig. 5, the pellet was resuspended in Matrigel and gently pipetted multiple time to homogenize. Two pieces of stacked tapes, each with a height of ∼450 μm, were attached to either side of a cover slide, and a 40-μl drop of the mixture was transferred to the center of that cover slide. The drop was sandwiched by placing another #1.5 cover slide on the top and transferred for imaging.

For preparing the sample in Fig. 7, the organoid pellet was fixed by resuspending it in 1 ml of 4% paraformaldehyde (Santa Cruz Biotechnology), followed by incubation at 4°C for 1 hour. In the middle of the fixation period, the organoids were gently resuspended once. To validate the thickness of organoids, the nuclei of cells were stained with 4′,6-diamidino-2-phenylindole (DAPI) (excitation: 402 nm, band-pass: 410 to 489 nm) after organoids’ recovery from the fixing solution using the same centrifugation setting as mentioned above. A 2× working solution of DAPI was prepared in PBS and added to the organoid pellet, followed by incubation at 4°C for 30 min. The organoids were washed twice with PBS to remove any unbound DAPI and centrifuged to recover organoids in a pellet. Next, 100 μl of PBS was added to the pellet, pipetted a few times, and then the suspension was dispensed at the center of a 50-mm μ-dish (Ibidi, low, polymer coverslip). Four milliliters of PBS was slowly added to wet the entire dish area and enable imaging using water-dipping objectives. Last, the dish containing the fixed and stained organoids was transferred for imaging.

#### Zebrafish embryos

Zebrafish were housed at UT Austin under IACUC protocol AUP-2023-00297. Zebrafish embryos were raised according to (*112*) and staged according to (*113*). Before imaging 24 hpf, embryos were fixed in 4% paraformaldehyde overnight at 4°C. Subsequently, they were placed in increasing concentrations of methanol in PBS until 100% methanol was reached and embryos were dehydrated. Embryos were then stored at −20°C. To prepare embryos for imaging embryos were rehydrated by placing them in increasing concentrations of PBS in the methanol/PBS solutions until 100% PBS was reached.

#### Spheroids

Cultures of MCF-7 breast adenocarcinoma cells (American Type Culture Collection, passages 10 to 15) were maintained in high-glucose Dulbecco’s Modified Eagle’s Medium (DMEM) containing fetal bovine serum (10%), penicillin-streptomycin (1×), and insulin (10 μg/ml) housed in a sterile incubator at 37°C, 5% CO_2_, and 99% humidity. For spheroid preparation, cells were harvested using Trypsin EDTA (0.25%) and counted using an automated cell counter (Countess, Thermo Fisher Scientific). Cells were then seeded in an Aggrewell 800 plate (STEMCELL Technologies) at a density of 300,000 cells per well. The microwells of the plate were precoated with an anti-adherence solution using a standardized protocol, and the plate was centrifuged at 300 rcf for 3 min after cell seeding. MCF-7 spheroids were allowed to form over a period of 12 to 24 hours in the Aggrewell plate in the incubator. For hydrogel embedded samples, such as the one shown in fig. S4, spheroids were collected by gentle agitation using a pipette and centrifugation at 100 rcf for 1 min. Collagen type IA–based Cellmatrix Cell culturing kit (Nitta Gelatin) was used for embedding the spheroids, and gels were cast on a 35-mm Mattek glass-bottom dish. On the day of imaging, cells were fixed using 4% paraformaldehyde treatment (gentle rocking for 3 hours) followed by washing using PBS. The gels were submerged in PBS for the entire duration of imaging. For suspension samples, such as the one shown in fig. S9, spheroids were collected by gentle agitation using a pipette and centrifugation at 100 rcf for 1 min. The spheroids were then fixed in suspension using 4% paraformaldehyde by gentle rocking, washed with PBS, and dropped onto a glass coverslip for imaging.

## Supplementary Materials

### The PDF file includes:

Supplementary Text Figs. S1 to S11 Table S1 Legends for movies S1 to S6 References
