# Supplementary material for: Inverse scattering in biological samples via beam propagation
Source: Sci Adv. 2026 Aug 21;12(34):eaec8678. doi: 10.1126/sciadv.aec8678 (PMC13496197; doi:10.1126/sciadv.aec8678)
Supplement: Supplementary file 1 [file sciadv.aec8678_sm.pdf]

Science Advances
AAAS

Supplementary Materials for

Inverse scattering in biological samples via beam propagation

Jeongsoo Kim *et al.*

Corresponding author: Shwetadwip Chowdhury, shwetadwip.chowdhury@utexas.edu

Sci. Adv. **12**, eaec8678 (2026)
DOI: 10.1126/sciadv.aec8678

The PDF file includes:

Supplementary Text
Figs. S1 to S11
Table S1
Legends for movies S1 to S6
References

Other Supplementary Material for this manuscript includes the following:

Movies S1 to S6

Supplementary Texts

Supplementary Note 1: Gradient derivation for multi-slice beam propagation (MSBP)

This section details the gradient derivations for the multi-slice beam propagation (MSBP) model. The gradient is critical in solving the inverse problem presented in Eq. 4 of the main manuscript, enabling iterative reconstruction of a sample's 3D refractive index (RI). We've incorporated additional terms in these derivations to account for the finite diffraction limit and the refocusing from the final sample layer to the image conjugate plane. Furthermore, we describe how the gradient form changes based on whether complex-valued field or amplitude-only measurements are used to solve the inverse problem.

<u>MSBP gradient derivation with complex-valued field measurements</u>

We first derive the MSBP gradient specifically for scenarios where complex-valued fields are measured (e.g., using interferometric approaches) and used directly in the reconstruction process. Our derivation largely follows the notation and strategies presented in Kamilov *et al.*'s original work (*50*), which utilized discrete matrices to represent optical operations. This formulation allows us to compactly express the MSBP model, summarized by Eqs. 2 and 3 in the main text, with just one equation expressed in discrete matrix form:

$$\mathbf{E}_m(\mathbf{x}) = \mathrm{diag}\big(\mathbf{T}_m(\mathbf{x})\big) \cdot \mathbf{H}_{\Delta z} \cdot \mathbf{E}_{m-1}(\mathbf{x}) \tag{S1}$$

Here, the electric field at the mth layer is denoted as $\mathbf{E}_m(\mathbf{x})$, where $m = 1,2, \ldots M$ for a M-layer sample. $\mathbf{x}$ denotes the *difference* between the sample's 3D RI and that of the surrounding homogenous media, n_0. The operator $\mathrm{diag}(\cdot)$ converts an input vector into a diagonal matrix, where the elements of the vector are placed along the main diagonal. $\mathbf{T}_m(\mathbf{x})$ denotes the transmittance function at the mth layer of the object, and $\mathbf{H}_{\Delta z}$ represents the optical propagation operation by the distance Δz between adjacent sample layers. The transmittance function is itself related to the object's RI via the relation $\mathbf{T}_m(\mathbf{x}) = \exp\left(j\frac{2\pi\, n_0}{\lambda}\Delta z\, \mathbf{x}_m\right)$, where λ is the imaging wavelength and $\mathbf{x}_m$ is the discrete variable representing RI-difference in *only* the sample's m-th layer (and zero everywhere else). Note that decomposing the sample into its constituent layers is mathematically equivalent to the summation $\mathbf{x} = \mathbf{x}_1 + \mathbf{x}_2 + \cdots \mathbf{x}_M$.

Beginning with a known illumination field incident to the sample (i.e., $\mathbf{E}_0$), repeated application of Eq. (S1) calculates the resulting field as it propagates layer-by-layer through the sample, up to the field $\mathbf{E}_M$ exiting the final M-th layer. When complex-valued fields are measured, this scattered field is directly related to the output of the MSBP forward model, and is a function of $\mathbf{x}$, i.e., $\mathbf{E}_M = \mathbf{E}_M(\mathbf{x})$.

In practice, $\mathbf{x}$ is not directly known and must be inferred from the measurements. This is referred to as solving the inverse-problem. A popular approach is to formulate this as a minimization problem that aims to find a distribution for $\mathbf{x}$ that minimizes the difference between the measured scattered fields and predictions by the MSBP forward model. This minimization process can be accomplished using various gradient-descent methods, highlighting the critical need to compute the gradient of the MSBP model.

Since $\mathbf{x}$ represents a 3D distribution and the forward model's predictions are 2D, reconstructing $\mathbf{x}$ from a single measurement is inherently ill-posed. To improve the posedness of

this inverse problem, we collect multiple measurements using various system configurations, such as angled illuminations and/or defocus planes. The goal then becomes finding a solution for $\mathbf{x}$ that minimizes the measurement-to-prediction difference across *all* these configurations. Sufficient diversity within the measurement dataset stabilizes the reconstruction of $\mathbf{x}$.

This inverse problem, expressed as a minimization across all measurements, can be represented by the notation below:

$$\hat{\mathbf{x}} = \underset{\mathbf{x}}{\operatorname{argmin}}\{\mathcal{D}_{\text{field}}(\mathbf{x}) + \mathcal{R}(\mathbf{x})\} \tag{S2}$$

where $\mathcal{D}_{\text{field}}(\mathbf{x})$ is the data fidelity term for complex-valued field measurements and $\mathcal{R}(\mathbf{x})$ is the regularization term (described later). The data fidelity term quantifies the discrepancy between the measured complex-valued fields and the field predictions from the MSBP model, and is defined as:

$$\mathcal{D}_{\text{field}}(\mathbf{x}) = \frac{1}{2L}\sum_{\ell=1}^{L}\left\|\mathbf{y}_{\text{field}}^{(\ell)} - \mathcal{G}_{\text{field}}^{(\ell)}(\mathbf{x})\right\|_{\text{L2}}^{2} \tag{S3}$$

where $\mathbf{y}_{\text{field}}^{(\ell)}$ denotes the complex-valued scattered field measured under the ℓ-th system configuration. For field-based measurements, we only varied the incident illumination angle – thus the ℓ-th system configuration corresponds directly to the ℓ-th illumination angle, with a total of L distinct angles. $\mathcal{G}_{\text{field}}^{(\ell)}(\mathbf{x})$ denotes the MSBP forward model for field-based measurements and can be decomposed into $\mathcal{G}_{\text{field}}^{(\ell)}(\mathbf{x}) = \mathbf{P}\cdot\mathbf{H}_{-\hat{z}}\cdot\mathbf{E}_{M}^{(\ell)}(\mathbf{x})$, where $\mathbf{H}_{-\hat{z}}$ denotes the discrete propagation kernel that back-propagates the last layer's output field $\mathbf{E}_{M}^{(\ell)}(\mathbf{x})$ by a distance $\hat{z}$ back to the conjugate image plane within the sample, and $\mathbf{P}$ represents convolution with the system's point spread function (PSF). In this work, we assume that the system PSF is diffraction-limited (i.e., no aberrations).

We now describe the derivation of the gradient of $\mathcal{D}_{\text{field}}$. For notational simplicity, we consider a single illumination angle and thus omit the index ℓ in the subsequent mathematical steps. Furthermore, since both $\mathbf{P}$ and $\mathbf{H}_{-\hat{z}}$ are known constants independent of the $\mathbf{x}$ variable, we group them into a single measurement operator $\mathbf{W} = \mathbf{P}\cdot\mathbf{H}_{-\hat{z}}$. With this simplification, field-based forward model can be expressed as $\mathcal{G}_{\text{field}}(\mathbf{x}) = \mathbf{W}\cdot\mathbf{E}_{M}(\mathbf{x})$, and the data fidelity term in Eq. (S3) can be expanded as follows:

$$\begin{aligned}\mathcal{D}_{\text{field}}(\mathbf{x}) &= \frac{1}{2}\|\mathbf{y}_{\text{field}} - \mathbf{W}\cdot\mathbf{E}_{M}(\mathbf{x})\|_{\text{L2}}^{2} \\ &= \frac{1}{2}\langle\mathbf{y}_{\text{field}},\mathbf{y}_{\text{field}}\rangle - \operatorname{Re}\{\langle\mathbf{y}_{\text{field}},\mathbf{W}\cdot\mathbf{E}_{M}(\mathbf{x})\rangle\} + \frac{1}{2}\langle\mathbf{W}\cdot\mathbf{E}_{M}(\mathbf{x}),\mathbf{W}\cdot\mathbf{E}_{M}(\mathbf{x})\rangle\end{aligned} \tag{S4}$$

Here, $\langle\ldots\rangle$ represents the inner product of two vectors, such that $\langle\mathbf{A},\mathbf{B}\rangle = \mathbf{A}^{H}\mathbf{B}$, where the H superscript denotes Hermitian transpose. Then, the gradient $\nabla_{\mathbf{x}}\mathcal{D}_{\text{field}}(\mathbf{x})$ can be computed as follows:

$$\begin{aligned}\nabla_{\mathbf{x}}\mathcal{D}_{\text{field}}(\mathbf{x}) &= -\frac{\partial}{\partial\mathbf{x}}\text{Re}\{\mathbf{y}_{\text{field}}^{H}\cdot\mathbf{W}\cdot\mathbf{E}_{M}(\mathbf{x})\}+\frac{1}{2}\frac{\partial}{\partial\mathbf{x}}\left[\left(\mathbf{W}\cdot\mathbf{E}_{M}(\mathbf{x})\right)^{H}\cdot\left(\mathbf{W}\cdot\mathbf{E}_{M}(\mathbf{x})\right)\right]\\ &= -\text{Re}\left\{\mathbf{y}_{\text{field}}^{H}\cdot\mathbf{W}\cdot\frac{\partial}{\partial\mathbf{x}}\mathbf{E}_{M}(\mathbf{x})\right\}+\text{Re}\left\{\mathbf{E}_{M}(\mathbf{x})^{H}\cdot\mathbf{W}^{H}\cdot\mathbf{W}\cdot\frac{\partial}{\partial\mathbf{x}}\mathbf{E}_{M}(\mathbf{x})\right\}\\ &= \text{Re}\left\{(\mathbf{W}\cdot\mathbf{E}_{M}(\mathbf{x})-\mathbf{y}_{\text{field}})^{H}\cdot\mathbf{W}\cdot\frac{\partial}{\partial\mathbf{x}}\mathbf{E}_{M}(\mathbf{x})\right\}\end{aligned} \tag{S5}$$

In practice, we are interested in the gradient as a column vector - accordingly, we take the Hermitian transpose. Recalling that $\mathcal{G}_{\text{field}}(\mathbf{x}) = \mathbf{W}\cdot\mathbf{E}_{M}(\mathbf{x})$, this transpose leads to the following expression:

$$\left(\nabla_{\mathbf{x}}\mathcal{D}_{\text{field}}(\mathbf{x})\right)^{H} = \text{Re}\left\{\left(\frac{\partial}{\partial\mathbf{x}}\mathbf{E}_{M}(\mathbf{x})\right)^{H}\cdot\mathbf{W}^{H}\cdot\left(\mathcal{G}_{\text{field}}(\mathbf{x})-\mathbf{y}_{\text{field}}\right)\right\} \tag{S6}$$

<u>Computing Eq. (S6) fulfills our objective to obtain the gradient of the MSBP model for complex-valued field measurements</u>. To achieve this, we first need to compute $\partial\mathbf{E}_{M}(\mathbf{x})/\partial\mathbf{x}$. We begin by differentiating both sides of Eq. (S1):

$$\begin{aligned}\frac{\partial}{\partial\mathbf{x}}\mathbf{E}_{m}(\mathbf{x}) &= \frac{\partial}{\partial\mathbf{x}}\left(\text{diag}\left(\mathbf{T}_{m}(\mathbf{x})\right)\cdot\mathbf{H}_{\Delta z}\cdot\mathbf{E}_{m-1}(\mathbf{x})\right)\\ &= \text{diag}\left(\mathbf{H}_{\Delta z}\cdot\mathbf{E}_{m-1}(\mathbf{x})\right)\cdot\frac{\partial}{\partial\mathbf{x}}\mathbf{T}_{m}(\mathbf{x})+\text{diag}\left(\mathbf{T}_{m}(\mathbf{x})\right)\cdot\mathbf{H}_{\Delta z}\cdot\frac{\partial}{\partial\mathbf{x}}\mathbf{E}_{m-1}(\mathbf{x})\end{aligned} \tag{S7}$$

Taking the Hermitian transpose to prepare for eventual substitution into Eq. (S6), we obtain:

$$\begin{aligned}\left(\frac{\partial}{\partial\mathbf{x}}\mathbf{E}_{m}(\mathbf{x})\right)^{H} &= \left(\frac{\partial}{\partial\mathbf{x}}\mathbf{E}_{m-1}(\mathbf{x})\right)^{H}\cdot\mathbf{H}_{\Delta z}^{H}\cdot\text{diag}\left(\overline{\mathbf{T}_{m}(\mathbf{x})}\right)\\ &\quad+\left(\frac{\partial}{\partial\mathbf{x}}\mathbf{T}_{M}(\mathbf{x})\right)^{H}\cdot\text{diag}\left(\overline{\mathbf{H}_{\Delta z}\cdot\mathbf{E}_{m-1}(\mathbf{x})}\right)\end{aligned} \tag{S8}$$

where the operation $\overline{\mathbf{A}}$ represents the complex conjugate of $\mathbf{A}$ and $\frac{\partial}{\partial\mathbf{x}}\mathbf{T}_{m}(\mathbf{x}) = j\frac{2\pi\, n_0}{\lambda}\Delta z\cdot\mathbf{T}_{m}(\mathbf{x})$.

Eq. (S8) shows that the derivative term $\partial\mathbf{E}_{m}(\mathbf{x})/\partial\mathbf{x}$ at the m-th sample layer can be expressed in terms of the derivative at the preceding layer, $\partial\mathbf{E}_{m-1}(\mathbf{x})/\partial\mathbf{x}$. Thus, to compute the derivative of the final sample layer $\partial\mathbf{E}_{M}(\mathbf{x})/\partial\mathbf{x}$, as required by the full MSBP gradient expression in Eq. (S6), one must recursively back-propagate the derivative terms through each layer of the sample. This recursion terminates with a boundary condition: the field at the $m = 0$ layer *before* the sample (i.e., $\mathbf{E}_0(\mathbf{x})$) is simply the incident illumination field, and is therefore independent of $\mathbf{x}$. Thus, its gradient with respect to $\mathbf{x}$ is zero:

$$\left(\frac{\partial}{\partial\mathbf{x}}\mathbf{E}_{0}(\mathbf{x})\right)^{H} = 0 \tag{S9}$$

Using the recursive relationship in Eq. (S8) for the case $m = M$, we can express the derivative term $\partial \mathbf{E}_M(\mathbf{x})/\partial \mathbf{x}$ in Eq. (S6) in terms of $\partial \mathbf{E}_{M-1}(\mathbf{x})/\partial \mathbf{x}$. Furthermore, by defining a residual term for the last sample layer as $\mathbf{r}_M = \mathbf{W}^H \cdot (\mathcal{G}_{\text{field}}(\mathbf{x}) - \mathbf{y}_{\text{field}})$, we can rewrite the MSBP gradient expression in Eq. (S6) as:

$$\left(\nabla_{\mathbf{x}} \mathcal{D}_{\text{field}}(\mathbf{x})\right)^H = \operatorname{Re}\left\{\left(\frac{\partial}{\partial \mathbf{x}} \mathbf{E}_{M-1}(\mathbf{x})\right)^H \cdot {\mathbf{H}_{\Delta z}}^H \cdot \operatorname{diag}\left(\overline{\mathbf{T}_M(\mathbf{x})}\right) \cdot \mathbf{r}_M + \left(\frac{\partial}{\partial \mathbf{x}} \mathbf{T}_M(\mathbf{x})\right)^H \cdot \operatorname{diag}\left(\overline{\mathbf{H}_{\Delta z} \cdot \mathbf{E}_{M-1}(\mathbf{x})}\right) \cdot \mathbf{r}_M\right\} \tag{S10}$$

Here, the term ${\mathbf{H}_{\Delta z}}^H \cdot \operatorname{diag}\left(\overline{\mathbf{T}_M(\mathbf{x})}\right) \cdot \mathbf{r}_M$ can be conceptually interpreted as the back-propagation of the original last-layer residual term $\mathbf{r}_M$. Thus, for $m \neq M$, we define the general form of a back-propagated residual as:

$$\mathbf{r}_{m-1} = {\mathbf{H}_{\Delta z}}^H \cdot \operatorname{diag}\left(\overline{\mathbf{T}_m(\mathbf{x})}\right) \cdot \mathbf{r}_m \tag{S11}$$

Thus, in the case of $m = M$, the back-propagated residual term $\mathbf{r}_{M-1}$ can take the place of ${\mathbf{H}_{\Delta z}}^H \cdot \operatorname{diag}\left(\overline{\mathbf{T}_M(\mathbf{x})}\right) \cdot \mathbf{r}_M$ in the gradient expansion in Eq. (S10):

$$\left(\nabla_{\mathbf{x}} \mathcal{D}_{\text{field}}(\mathbf{x})\right)^H = \operatorname{Re}\left\{\left(\frac{\partial}{\partial \mathbf{x}} \mathbf{E}_{M-1}(\mathbf{x})\right)^H \cdot \mathbf{r}_{M-1} + \left(\frac{\partial}{\partial \mathbf{x}} \mathbf{T}_M(\mathbf{x})\right)^H \cdot \operatorname{diag}\left(\overline{\mathbf{H}_{\Delta z} \cdot \mathbf{E}_{M-1}(\mathbf{x})}\right) \cdot \mathbf{r}_M\right\} \tag{S12}$$

Notably, the expression in Eq. (S12) is simply an expansion of the MSBP gradient formulation first introduced in Eq. (S6), except where the derivative term $\partial \mathbf{E}_M(\mathbf{x})/\partial \mathbf{x}$ at the final sample layer is now expressed in terms of the derivative term from the preceding layer $\partial \mathbf{E}_{M-1}(\mathbf{x})/\partial \mathbf{x}$.

By repeatedly applying Eqs. (S8) and (S11), the derivative term $\partial \mathbf{E}_{M-1}(\mathbf{x})/\partial \mathbf{x}$ in Eq. (S12) can be recursively rewritten in terms of derivative terms at progressively earlier sample layers until the boundary condition defined in Eq. (S9) is reached, thereby terminating the recursion. At this point, the MSBP gradient can be written simply as a summation of terms across all sample layers:

$$\left(\nabla_{\mathbf{x}} \mathcal{D}_{\text{field}}(\mathbf{x})\right)^H = \operatorname{Re}\left\{\sum_{m=1}^{M}\left[\left(\frac{\partial}{\partial \mathbf{x}} \mathbf{T}_m(\mathbf{x})\right)^H \cdot \operatorname{diag}\left(\overline{\mathbf{H}_{\Delta z} \cdot \mathbf{E}_{m-1}(\mathbf{x})}\right) \cdot \mathbf{r}_m\right]\right\} \tag{S13}$$

In practice, this summation is computed using the efficient back-propagation scheme proposed by Kamilov *et al*. (*50*), which calculates the derivative terms layer-by-layer *backwards* through the sample.

We begin with an initial estimate of the sample's 3D RI-difference distribution $\mathbf{x}$, either by setting it to zero (signifying a uniform RI equal to the surrounding medium) or by using a weak-scattering approximation from Fourier diffraction theory. The incident illumination field $\mathbf{E}_0$ is then propagated through the sample while storing the intermediate fields $\mathbf{E}_m(\mathbf{x})$ at each layer $m = 1,2, \dots M$. The residual term at the last layer is then computed as described earlier, $\mathbf{r}_M = \mathbf{W}^H \cdot (\mathcal{G}_{\text{field}}(\mathbf{x}) - \mathbf{y}_{\text{field}})$. Here, we introduce the adjoint variable $\mathbf{s}_M = 0$, which serves as the starting point for the recursive summation used to compute the gradient, as shown in Eq. (S13).

Next, the process to compute the MSBP gradient is initiated by back-propagating both the adjoint variable and the residual term from the final sample layer. The residual term's back-propagation follows Eq. (S11), while the adjoint variable's back-propagation follows the strategy depicted in Eq. (S13), and can be expressed as:

$$\mathbf{s}_{m-1} = \mathbf{s}_m + \left(\frac{\partial}{\partial \mathbf{x}} \mathbf{T}_m(\mathbf{x})\right)^H \cdot \operatorname{diag}\left(\overline{\mathbf{H}_{\Delta z} \cdot \mathbf{E}_{m-1}(\mathbf{x})}\right) \cdot \mathbf{r}_m \tag{S14}$$

Repeatedly evaluating Eqs. (S11) and (S14) is equivalent to solving the gradient summation in Eq. (S13) layer-by-layer *backwards* through the sample. Finally, the MSBP gradient is obtained as the real component of the accumulated adjoint variable at the input plane:

$$\left(\nabla_{\mathbf{x}} \mathcal{D}_{\text{field}}(\mathbf{x})\right)^H = \operatorname{Re}\{\mathbf{s}_0\} \tag{S15}$$

Notably, this recursive back-propagation scheme eliminates the need to explicitly store or operate on large matrices, making it highly suitable for iterative optimization. Once the gradient is computed, it is used to iteratively update the voxel values in the current estimate of the sample $\mathbf{x}$. The magnitude of each iterative update is tuned by a step-size parameter. Repeating this process gradually refines $\mathbf{x}$, driving it toward a solution for the sample's 3D RI-difference distribution. Adding the converged distribution of $\mathbf{x}$ with the RI of the surrounding media n_0 yields the sample's true 3D RI.

To stabilize this process and promote physically plausible solutions, a regularization term $\mathcal{R}(\mathbf{x})$ is often integrated into the minimization problem, as shown earlier in Eq. (S2). In this work, we use a 3D isotropic total-variation (TV) prior (*114*), which promotes piecewise-smooth reconstructions. This regularization is implemented using a proximal operator:

$$\operatorname{prox}\{\mathbf{x}, \tau\} = \underset{\mathbf{z}}{\operatorname{argmin}} \left\{\frac{1}{2} \|\mathbf{x} - \mathbf{z}\|_{\text{L2}}^2 + \tau \cdot \operatorname{TV}(\mathbf{z})\right\} \tag{S16}$$

where $\mathbf{x}$ represents the 3D RI variable being optimized, TV denotes the 3D total-variation operation (*115*), and τ is a tuning parameter that controls the strength of TV regularization.

Notably, both the step-size and regularization parameters are empirically tuned for optimal performance. We discuss in a later section how we select these parameters.

MSBP gradient derivation with amplitude-only field measurements:

When the full complex-valued field cannot be measured (e.g., most non-interferometric systems), an inverse problem can still be formulated to reconstruct 3D RI. In this case, the goal is to minimize the difference between raw amplitude measurements and the *amplitude* of the scattered fields predicted by the MSBP model. This method was used by Chowdhury *et al*. (*34*) for inverse-scattering, but a rigorous derivation of the gradient had not yet been presented.

To start this derivation, we first express below the updated inverse problem for *amplitude-only* measurements:

$$\hat{\mathbf{x}} = \underset{\mathbf{x}}{\operatorname{argmin}} \left\{ \mathcal{D}_{\text{amp}}(\mathbf{x}) + \mathcal{R}(\mathbf{x}) \right\} \tag{S17}$$

where $\mathcal{D}_{\text{amp}}(\mathbf{x})$ is now the data fidelity term that quantifies the difference between the *amplitude* measurements and the *amplitude* component of the MSBP field predictions, across all L illumination angles:

$$\mathcal{D}_{\text{amp}}(\mathbf{x}) = \frac{1}{2L} \sum_{\ell=1}^{L} \left\| \mathbf{y}_{\text{amp}}^{(\ell)} - \mathcal{G}_{\text{amp}}^{(\ell)}(\mathbf{x}) \right\|_{\text{L2}}^{2} \tag{S18}$$

We see here that Eq. (S18) closely resembles Eq. (S3) earlier. The major differences are: 1) $\mathbf{y}_{\text{amp}}^{(\ell)}$ now denote the *amplitude* measurement of the sample illuminated under the ℓ-th illumination angle; and 2) $\mathcal{G}_{\text{amp}}^{(\ell)}(\mathbf{x})$ denotes the MSBP forward model for the corresponding *amplitude-only* measurement, equivalent to the amplitude component of the scattered field.

In terms of the variables introduced in Eq. (S3), this is equivalent to $\mathcal{G}_{\text{amp}}^{(\ell)}(\mathbf{x}) = \left| \mathcal{G}_{\text{field}}^{(\ell)}(\mathbf{x}) \right|$,
212 or equivalently, $\mathcal{G}_{\text{amp}}^{(\ell)}(\mathbf{x}) = \left| \mathbf{W} \cdot \mathbf{E}_{M}^{(\ell)}(\mathbf{x}) \right|$, where we recall that $\mathbf{W} = \mathbf{P} \cdot \mathbf{H}_{-\hat{z}}$. We now start the derivation of the gradient for $\mathcal{D}_{\text{amp}}$. Similar as before, we also now omit the index ℓ for notational simplicity. With these notational substitutions, Eq. (S18) can be expanded:

$$\begin{aligned} \mathcal{D}_{\text{amp}}(\mathbf{x}) &= \frac{1}{2} \left\| \mathbf{y}_{\text{amp}} - \mathcal{G}_{\text{amp}}(\mathbf{x}) \right\|_{\text{L2}}^{2} \\ &= \frac{1}{2} \langle \mathbf{y}_{\text{amp}}, \mathbf{y}_{\text{amp}} \rangle - \operatorname{Re}\left\{ \langle \mathbf{y}_{\text{amp}}, \mathcal{G}_{\text{amp}}(\mathbf{x}) \rangle \right\} + \frac{1}{2} \langle \mathcal{G}_{\text{amp}}(\mathbf{x}), \mathcal{G}_{\text{amp}}(\mathbf{x}) \rangle \end{aligned} \tag{S19}$$

Following similar steps as described earlier in Eqs. (S4) – (S5), the gradient $\left(\nabla_{\mathbf{x}} \mathcal{D}_{\text{amp}}(\mathbf{x}) \right)^{H}$ can be expressed as follow:

$$\begin{aligned}\left(\nabla_{\mathbf{x}}\mathcal{D}_{\text{amp}}(\mathbf{x})\right)^H &= \left(-\frac{\partial}{\partial\mathbf{x}}\text{Re}\{\mathbf{y}_{\text{amp}}^H\cdot\mathcal{G}_{\text{amp}}(\mathbf{x})\}+\frac{1}{2}\frac{\partial}{\partial\mathbf{x}}\left[\left(\mathcal{G}_{\text{amp}}(\mathbf{x})\right)^H\cdot\mathcal{G}_{\text{amp}}(\mathbf{x})\right]\right)^H\\ &= \left(-\text{Re}\left\{\mathbf{y}_{\text{amp}}^H\cdot\frac{\partial}{\partial\mathbf{x}}\mathcal{G}_{\text{amp}}(\mathbf{x})\right\}+\text{Re}\left\{\left(\mathcal{G}_{\text{amp}}(\mathbf{x})\right)^H\cdot\frac{\partial}{\partial\mathbf{x}}\mathcal{G}_{\text{amp}}(\mathbf{x})\right\}\right)^H\\ &= \text{Re}\left\{\left(\frac{\partial}{\partial\mathbf{x}}\mathcal{G}_{\text{amp}}(\mathbf{x})\right)^H\left(\mathcal{G}_{\text{amp}}(\mathbf{x})-\mathbf{y}_{\text{amp}}\right)\right\}\end{aligned} \tag{S20}$$

<u>*Computing Eq. (S20) fulfills our objective to obtain the gradient of the MSBP model for amplitude-only measurements*</u>. To achieve this, we first need to compute $\frac{\partial\mathcal{G}_{\text{amp}}(\mathbf{x})}{\partial\mathbf{x}}$. Recalling that $\mathcal{G}_{\text{amp}}(\mathbf{x}) = |\mathbf{W}\cdot\mathbf{E}_M(\mathbf{x})|$ is itself a function of $\mathbf{E}_M(\mathbf{x})$, we can apply the chain rule to expand this derivative:

$$\frac{\partial}{\partial\mathbf{x}}\mathcal{G}_{\text{amp}}(\mathbf{x}) = \frac{\partial\mathcal{G}_{\text{amp}}(\mathbf{x})}{\partial\mathbf{E}_M(\mathbf{x})}\cdot\frac{\partial\mathbf{E}_M(\mathbf{x})}{\partial\mathbf{x}} \tag{S21}$$

The first term in this chain rule expansion, $\frac{\partial\mathcal{G}_{\text{amp}}(\mathbf{x})}{\partial\mathbf{E}_M(\mathbf{x})}$, can also be expanded with the chain rule:

$$\begin{aligned}\frac{\partial\mathcal{G}_{\text{amp}}(\mathbf{x})}{\partial\mathbf{E}_M(\mathbf{x})} &= \frac{\partial|\mathbf{W}\cdot\mathbf{E}_M(\mathbf{x})|}{\partial\mathbf{E}_M(\mathbf{x})}\\ &= \text{diag}\left(\frac{\mathbf{W}\cdot\mathbf{E}_M(\mathbf{x})}{|\mathbf{W}\cdot\mathbf{E}_M(\mathbf{x})|}\right)^H\cdot\mathbf{W}\end{aligned} \tag{S22}$$

Here, we recall from Eqs. (S3) and (S4) that $\mathcal{G}_{\text{field}}(\mathbf{x}) = \mathbf{W}\cdot\mathbf{E}_m(\mathbf{x})$. Thus, Eq. (S22) can be re-expressed *in terms of the MSBP forward model for complex-valued measurements:*

$$\begin{aligned}\frac{\partial\mathcal{G}_{\text{amp}}(\mathbf{x})}{\partial\mathbf{E}_M(\mathbf{x})} &= \text{diag}\left(\frac{\mathcal{G}_{\text{field}}(\mathbf{x})}{|\mathcal{G}_{\text{field}}(\mathbf{x})|}\right)^H\cdot\mathbf{W}\\ &= \text{diag}\left(e^{-j\,\angle\mathcal{G}_{\text{field}}(\mathbf{x})}\right)\cdot\mathbf{W}\end{aligned} \tag{S23}$$

The last line in Eq. (S23) was derived from the first by applying three basic mathematical rules: (1) for any complex number z, the operation $\frac{z}{|z|}$ directly yields its phase component, i.e., $\exp(j\,\angle z)$; (2) the Hermitian transpose of a diagonal matrix is equivalent to simply conjugating its diagonal elements; and (3) the complex conjugate of a pure-phase term simply negates the phase, i.e., $\overline{\exp(j\angle z)} = \exp(-j\angle z)$.

By substituting Eqs. (S21) and (S23) into the $\frac{\partial\mathcal{G}_{\text{amp}}(\mathbf{x})}{\partial\mathbf{x}}$ term in Eq. (S20), we can rewrite the gradient for $\mathcal{D}_{\text{amp}}(\mathbf{x})$:

$$\begin{aligned}\left(\nabla_{\mathbf{x}}\mathcal{D}_{\text{amp}}(\mathbf{x})\right)^H &= \text{Re}\left\{\left(\text{diag}\left(e^{-j\,\angle\mathcal{G}_{\text{field}}(\mathbf{x})}\right)\cdot\mathbf{W}\cdot\frac{\partial\mathbf{E}_M(\mathbf{x})}{\partial\mathbf{x}}\right)^H\left(\mathcal{G}_{\text{amp}}(\mathbf{x})-\mathbf{y}_{\text{amp}}\right)\right\}\\ &= \text{Re}\left\{\left(\frac{\partial\mathbf{E}_M(\mathbf{x})}{\partial\mathbf{x}}\right)^H\cdot\mathbf{W}^H\cdot\ \text{diag}\left(e^{j\angle\mathcal{G}_{\text{field}}(\mathbf{x})}\right)\left(\mathcal{G}_{\text{amp}}(\mathbf{x})-\mathbf{y}_{\text{amp}}\right)\right\}\end{aligned}\tag{S24}$$

Lastly, recall that $\mathcal{G}_{\text{amp}}(\mathbf{x})$ is simply the amplitude of $\mathcal{G}_{\text{field}}(\mathbf{x})$, i.e., $\mathcal{G}_{\text{amp}}(\mathbf{x}) = |\mathcal{G}_{\text{field}}(\mathbf{x})|$. Using the basic property of complex polar form, $z = |z|\exp(j\ \angle z)$, Eq. (S24) can be simplified:

$$\left(\nabla_{\mathbf{x}}\mathcal{D}_{\text{amp}}(\mathbf{x})\right)^H = \text{Re}\left\{\left(\frac{\partial\mathbf{E}_M(\mathbf{x})}{\partial\mathbf{x}}\right)^H\cdot\mathbf{W}^H\cdot\left(\mathcal{G}_{\text{field}}(\mathbf{x})-\ \text{diag}\left(e^{j\angle\mathcal{G}_{\text{field}}(\mathbf{x})}\right)\cdot\mathbf{y}_{\text{amp}}\right)\right\}\tag{S25}$$

Here, we see that the gradient of $\mathcal{D}_{\text{amp}}(\mathbf{x})$, initially presented in Eq. (S20) in terms of $\mathcal{G}_{\text{amp}}(\mathbf{x})$, is now expressed in terms of $\mathcal{G}_{\text{field}}(\mathbf{x})$, i.e., the forward model for *complex-valued measurements*. In fact, this gradient expression is virtually identical to that of $\mathcal{D}_{\text{field}}(\mathbf{x})$ in Eq. (S6). The *only* difference is that the residual term in Eq. (S25) combines the amplitude-only measurements $\mathbf{y}_{\text{amp}}$ with the phase term of $\mathcal{G}_{\text{field}}(\mathbf{x})$, effectively generating a synthetic complex-valued residual. Both the recursive back-propagation used to compute $\frac{\partial\mathbf{E}_M(\mathbf{x})}{\partial\mathbf{x}}$ and the overall iterative optimization process to solve for $\mathbf{x}$ with regularization follow the same approach as that of the field-based MSBP model described earlier in Eqs. (S6) – (S17).

Notably, although the mathematical difference between the gradient terms for complex-valued and amplitude-only MSBP forward models appears minor, it leads to substantial differences in the reconstructed RI distributions, as discussed and demonstrated in the main manuscript.

MSBP gradient with amplitude-only field measurements and computational defocusing:

As shown in **Fig. 5** of the main manuscript, RI reconstructions from amplitude-only measurements outperformed those using complex-valued field data. We speculated that this is due to ambiguities introduced by wrapped phase, which likely misdirect the inverse solver's convergence when included with the $\mathcal{G}_{\text{field}}(\mathbf{x})$ term in the field-based data-fidelity expression in Eq. (S3). By using only the amplitude component of the complex-valued field within the data-fidelity term (Eq. (S18)), we observed more accurate RI reconstructions.

However, we observed that completely discarding phase information introduced shadowing artifacts, particularly beneath sample regions containing high-RI structures (see **Fig. 5** in main manuscript). To retain useful phase information while avoiding phase-wrapping issues within the data-fidelity term, we used defocused amplitude measurements. Although this could be done physically by defocusing the sample, we instead took advantage of our interferometric system's ability to capture complex-valued field data. We computationally defocused this field to selected virtual planes and extracted only the amplitude components, which were then treated as the "measurements" in the inverse solver. This approach embedded phase information into the amplitude measurements via defocus, allowing phase to enhance RI reconstruction without introducing errors from wrapped phase. This highlights a key advantage of incorporating defocus diversity into the measurement dataset when performing amplitude-based inverse scattering.

Here, we present the MSBP gradient formulation for defocused amplitude-only measurements. Let $z^{(k)}$ represents the axial distance from the image plane to the kth defocused

plane, where $k = 1,2,\ldots,K$. Here, K denotes the total number of defocused planes. As before, we omit the parameter ℓ denoting the illumination number for notational simplicity. Given a complex-valued field $\mathbf{y}_{\text{field}}$ at the conjugate image plane within the sample volume, we denote the *amplitude* of this field *after* being defocused to the kth plane as:

$$\mathbf{y}_{\text{amp}}^{(k)} = \left|\mathbf{H}_{z^{(k)}} \cdot \mathbf{y}_{\text{field}}\right| \quad \text{(S26)}$$

Here, $\mathbf{H}_{z^{(k)}}$ denotes the discrete propagation kernel used to propagate the field by a distance $z^{(k)}$. Similarly, the MSBP model prediction for each kth defocused amplitude measurement is given by $\mathcal{G}_{\text{amp}}^{(k)}(\mathbf{x}) = \left|\mathbf{H}_{z^{(k)}} \cdot \mathcal{G}_{\text{field}}(\mathbf{x})\right|$, where we recall that $\mathcal{G}_{\text{field}}(\mathbf{x})$ is specifically the model prediction for the complex-valued *field* at the image conjugate plane.

Thus, the total data fidelity term $\mathcal{D}_{\text{amp}}(\mathbf{x})$ can be expressed as a simple summation of individual terms that compare the defocused amplitude measurements to the defocused amplitude model predictions, $\mathcal{D}_{\text{amp}}^{(k)}(\mathbf{x})$:

$$\mathcal{D}_{\text{amp}}(\mathbf{x}) = \sum_{k=1}^{K} \mathcal{D}_{\text{amp}}^{(k)}(\mathbf{x}) \quad \text{(S27)}$$

$$\text{where:} \quad \mathcal{D}_{\text{amp}}^{(k)}(\mathbf{x}) = \tfrac{1}{2}\left\| \mathbf{y}_{\text{amp}}^{(k)} - \mathcal{G}_{\text{amp}}^{(k)}(\mathbf{x}) \right\|_{\text{L2}}^{2} \quad \text{(S28)}$$

Computing the gradient of $\mathcal{D}_{\text{amp}}(\mathbf{x})$ is equivalent to adding together the gradient of each individual term $\mathcal{D}_{\text{amp}}^{(k)}(\mathbf{x})$. Notably, the mathematical form $\mathcal{D}_{\text{amp}}^{(k)}$ is equivalent to that shown previously in Eq. (S19) and thus the same steps taken earlier can be used to compute the gradient.

Supplementary Note 2: Choosing parameters for step-size and regularization

Since the MSBP-based inverse problem is nonconvex, the final 3D RI reconstruction is sensitive to parameter choices. In particular, accurate results require careful tuning of the step size and regularization parameters. Poor choices can cause artifacts like over-smoothing, contrast loss, or convergence failure. Systematic, empirical tuning is therefore essential for achieving accurate and robust RI reconstructions.

To address this, we performed a comprehensive grid search over a range of step sizes and regularization parameters. For each parameter pair, we compared the reconstructed RI of various types of calibration microspheres with their RI values known from literature (*116*). These results are summarized in **Figure S1** below. Notably, all reconstruction results reflect fully converged values.

Figure S1A specifically shows the reconstructed RI values of a 10 µm polystyrene microsphere for 36 combinations of step size (ranging from 0.5×10^{-4} to 3×10^{-4}) and regularization strength (0.5×10^{-3} to 3×10^{-3}), each sampled uniformly at six intervals. Each grid point reflects the reconstruction result for a specific parameter pair, illustrating that both parameters influence reconstructed RI values. To visually isolate their effects, we varied one parameter while keeping the other fixed. The resulting effects are illustrated in **Figure S1(B, C)**,

which show center-plane *xy* cross-sections of the reconstructed RI for a polystyrene microsphere. In **Figure S1B**, we specifically fixed the step size (1.5×10^{-4}) and observed that increasing the regularization parameter produced progressively lower RI values. This trend reflects the smoothing effect of regularization, which suppresses noise and fine details but can underestimate RI contrast. Conversely, **Figure S1C** fixes the regularization strength (0.5×10^{-3}) and varies the step size. Here, larger step sizes led to higher converged RI values. However, excessively large steps risk overshooting and instability, especially without sufficient regularization. Together, these results highlight the distinct yet complementary roles of step size and regularization strength, and underscore the need for careful parameter balancing to ensure accurate and stable RI reconstruction.

To identify a step size and regularization combination that consistently produces accurate and stable reconstructions, we evaluated reconstruction errors for calibration microspheres

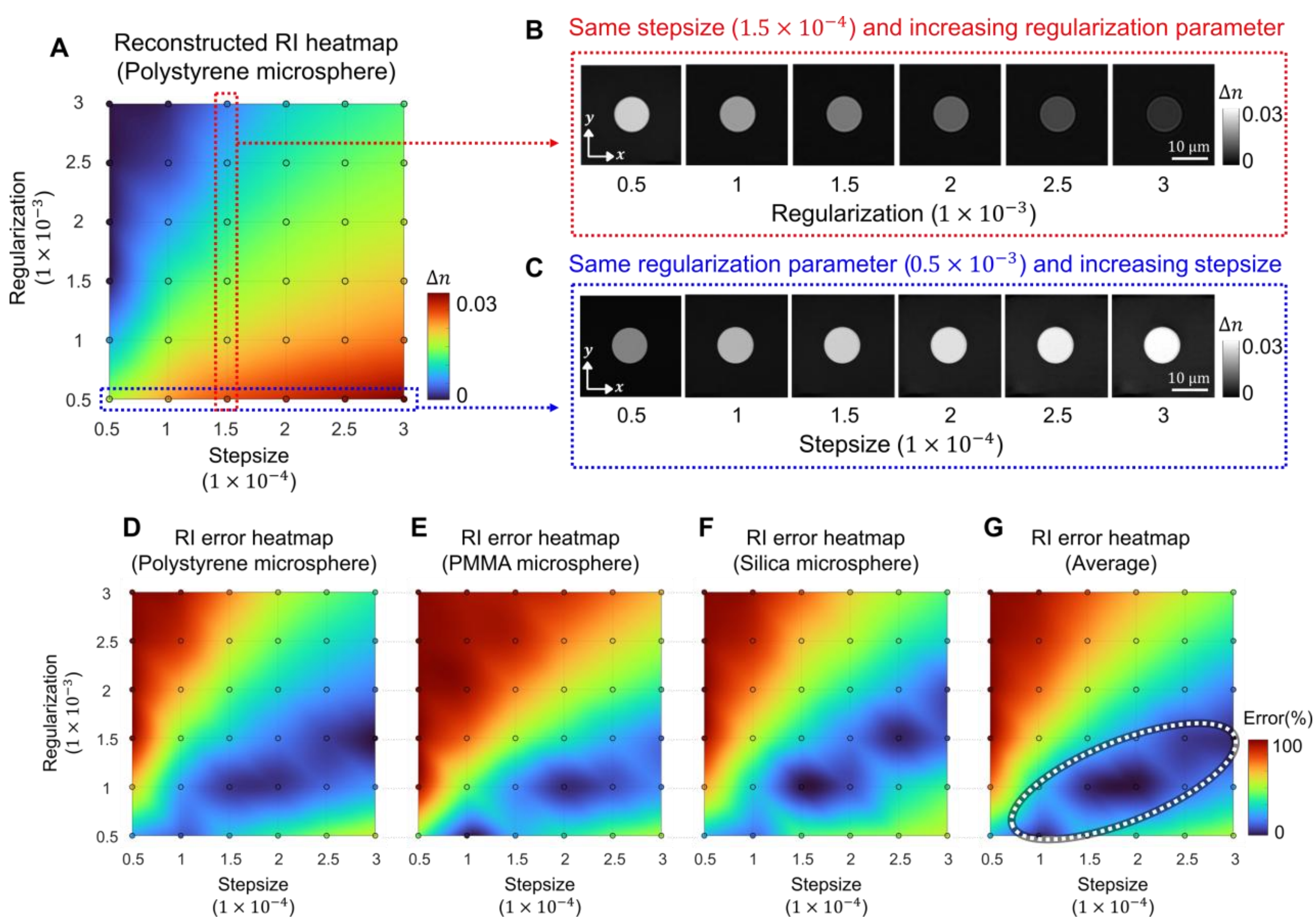

Figure S1. Sensitivity analysis for step size and regularization in MSBP-based RI reconstruction. (A) Reconstructed RI-difference values (Δn) of a 10 µm diameter polystyrene microsphere are shown across 36 combinations of step size and regularization parameters. Central xy-slices are shown from reconstructed RI volumes from the polystyrene microsphere with **(B)** increasing regularization (at fixed step size), showing that stronger regularization suppresses reconstructed Δn; and **(C)** increasing step size (at fixed regularization), illustrating that larger step sizes increases reconstructed Δn. **(D-F)** Error maps are shown comparing reconstructed Δn to ground-truth Δn for samples composed of **(D)** 10 µm diameter polystyrene microspheres, **(E)** 10 µm diameter polymethyl methacrylate (PMMA) microspheres, and **(F)** 5 µm diameter silica microspheres. **(G)** Average error map across all three microsphere types reveal a consistent low-error region (outlined in dashed white), suggesting a robust and generalizable parameter choice for step-size and regularization.

composed of different materials with varying RI (n_{sample}). Each microsphere sample was immersed in index-matching oils (Cargille) also with varying RI (n_{oil}), resulting in distinct RI-differences per sample ($\Delta n = n_{\text{sample}} - n_{\text{oil}}$). For each sample, the same parameter sweep as shown in **Figure S1A** was used to reconstruct 3D RI-difference maps via MSBP. Cross-sectional Δn values from these maps were then compared with the ground-truth values derived from known literature values of n_{sample} and n_{oil}. These results are presented as RI error maps below for: [**Figure S1D**] 10 µm diameter polystyrene microspheres ($n_{\text{sample}} = 1.5874$, $n_{\text{oil}} = 1.5682$); [**Figure S1E**] 10 µm diameter polymethyl methacrylate (PMMA) microspheres ($n_{\text{sample}} = 1.4886$, $n_{\text{oil}} = 1.4701$), and [**Figure S1F**] 5 µm diameter silica microspheres ($n_{\text{sample}} = 1.457$, $n_{\text{oil}} = 1.4336$).

As shown in the error maps in **Figure S1(D-F)**, reconstruction error varies with different combinations of step size and regularization strength. However, across all microsphere samples, we observe that the regions of lowest error consistently occur at similar parameter combinations. This trend is summarized in **Figure S1G**, which displays the mean error across all three microsphere types. A low-error region shared across all error maps is highlighted by a white dashed contour. Based on this observation, we selected parameter values from within this region for all subsequent RI reconstructions.

We note that this parameter combination was selected to produce robust and consistent quantitative accuracy across calibration samples that were weakly-scattering – more strongly scattering or structurally complex biological specimens may require different regularization strengths or step-size settings. In such cases, stronger regularization may be beneficial for stabilizing the optimization, at the cost of increased smoothing, whereas weaker regularization may preserve fine structures but risk instability.

Supplementary Note 3: Comparing phase measurements of scattering phantoms and biological samples

With field-based MSBP reconstruction, we observed that scattering phantoms could be successfully reconstructed when a Rytov-approximation-based initial guess was used (**Fig. 4**), whereas biological samples failed to reconstruct properly even under the same initialization (**Fig. 5**). To investigate this discrepancy and identify the underlying factors, we examined the complex-valued scattered fields from both the phantoms and biological samples to identify any visual markers that may distinguish between these two classes of samples. We measured complex-valued scattered fields using the off-axis interferometric setup shown in **Fig. 2** of the main manuscript.

Figure S2 compares phase measurements from the field scattered from the phantoms and biological samples under both orthogonal and oblique illumination angles. Specifically, **Figure S2(A-D)** show that phase measurements from phantoms with thicknesses of 40 µm, 60 µm, 80 µm, and 100 µm, respectively, displayed phase wraps primarily at the center of the phantoms, especially under oblique illumination angles. This accounts for the central artifacts commonly observed in field-based MSBP reconstructions without an initial guess (see **Fig. 4**), as phase-wrapping-induced ambiguities were most severe in the core of the phantom volume. With a sufficiently warm initial guess based on the Rytov weak-scattering approximation, these ambiguities were resolved *specifically in the phantoms*, suggesting that a reasonably accurate initialization helps the MSBP inverse solver avoid local minima caused by phase wrapping.

However, **Figure S2(E-G)** reveal that the phase measurements of an intestinal organoid, a wild-type *C. elegans*, and a lipid-rich mutant *C. elegans* exhibit far more chaotic and spatially complex phase- wrapping patterns than those of the scattering phantoms. As we see in **Fig. 5** (main text) and **Figure S5** for the organoid and mutant *C. elegans*, respectively, these types of samples cannot be accurately reconstructed even with Rytov-based initialization. This suggests that the samples' chaotic phase-wrapping behavior, which reflect their heterogeneously scattering characteristics, renders the Rytov approximation insufficient as a warm initial guess. In contrast, the scattering phantoms achieve successful reconstruction with the same initialization likely because they exhibit fewer and more structured phase wraps.

Notably, the mutant *C. elegans* exhibited the most severe and spatially intricate phase-wrapping among the samples. This strain was genetically modified to overproduce lipids, increasing its scattering strength and leading to stronger phase delays and wrapping. The contrast between the phase measurements of the wild-type and mutant *C. elegans* highlights how the severity of phase-wrapping can serve as a useful heuristic for estimating a sample's scattering strength. While not a quantitative measure, the structure and complexity of the wrapped phase offers insight into the degree of tissue heterogeneity and the anticipated challenges of computational reconstruction with field-based MSBP.

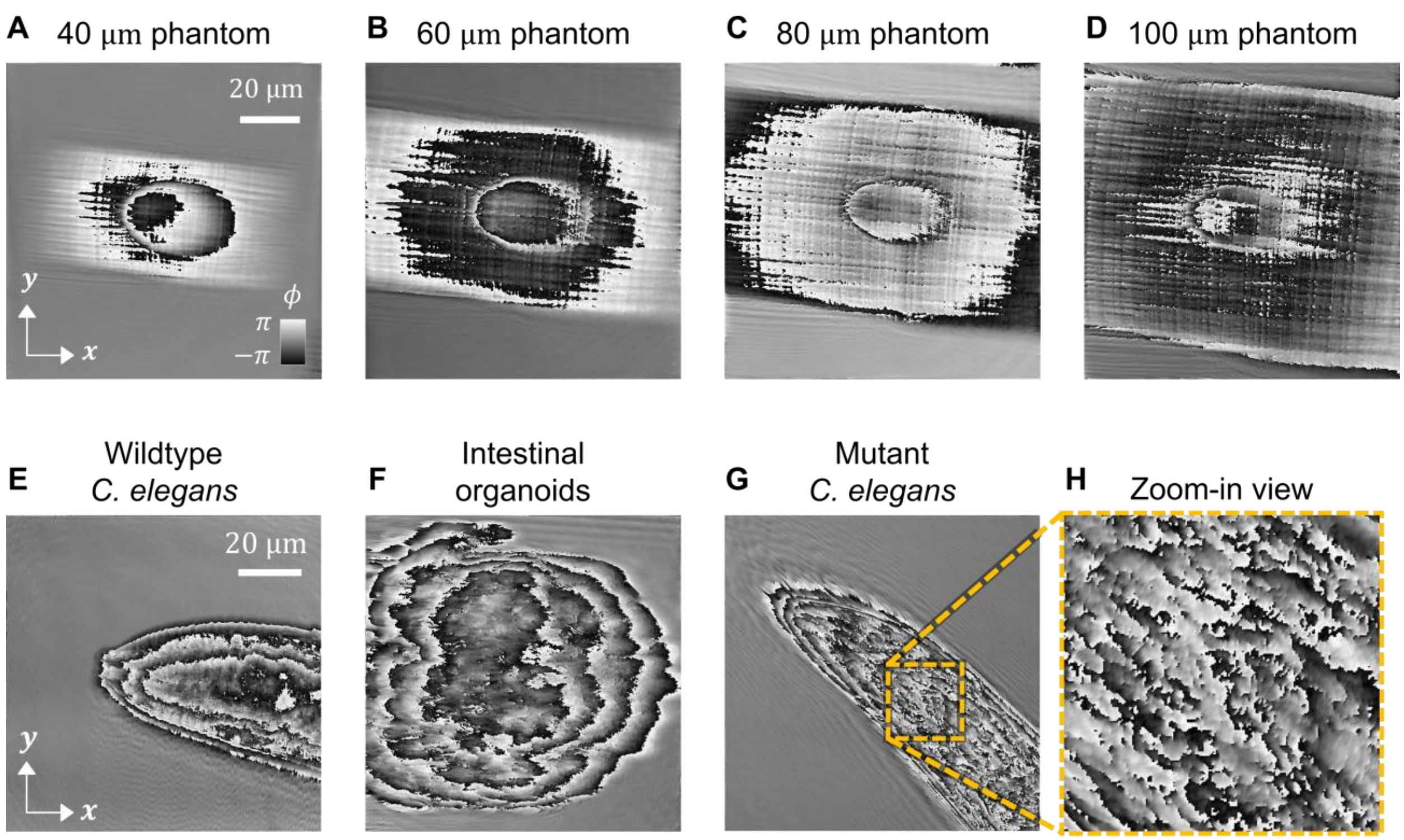

Figure S2. Phase measurements of scattering phantom and biological samples under angled illumination. (A-D) Measured phase maps of scattering phantom with thickness of 40 μm, 60 μm, 80 μm, and 100 μm, respectively. **(E-G)** Phase measurements of wild-type *C. elegans*, intestinal organoid, and mutant *C. elegans* strain (known for increased lipid synthesis), respectively, under oblique illumination, exhibiting severe and spatially complex phase-wrapping. **(H)** Zoom-in view to the phase measurement of the mutant *C. elegans* shows chaotic phase-wrapping, indicating strong tissue heterogeneity.

Supplementary Note 4: Effects of phase-wrapping in MSBP-based reconstruction

In the main text, we speculated that the strong artifacts in the 3D RI reconstructions using complex field-based MSBP stemmed from phase-wrapping in the measurements. To further show evidence of this, we performed a controlled numerical experiment in which we simulated two digital phantoms with identical geometry but different RI contrast levels, chosen such that one induced phase wrapping while the other did not. All other aspects of the simulation (e.g., illumination angles, numerical aperture, sampling grid, noise level, reconstruction parameters, etc) were kept strictly identical. In these simulations, the digital phantoms were imaged through a numerical aperture of 1.0. The phantoms were each illuminated at 100 distinct angles. As in real experiments, the amplitude-only MSBP reconstruction used only the amplitude components of the simulated field measurements in the inverse-solver, whereas the complex-field MSBP reconstruction used the full complex-valued fields.

Figure S3(A, B) show the RI values for the low RI-contrast phantom as well as the simulated amplitude/phase measurements captured at normal and high illumination angle, respectively.

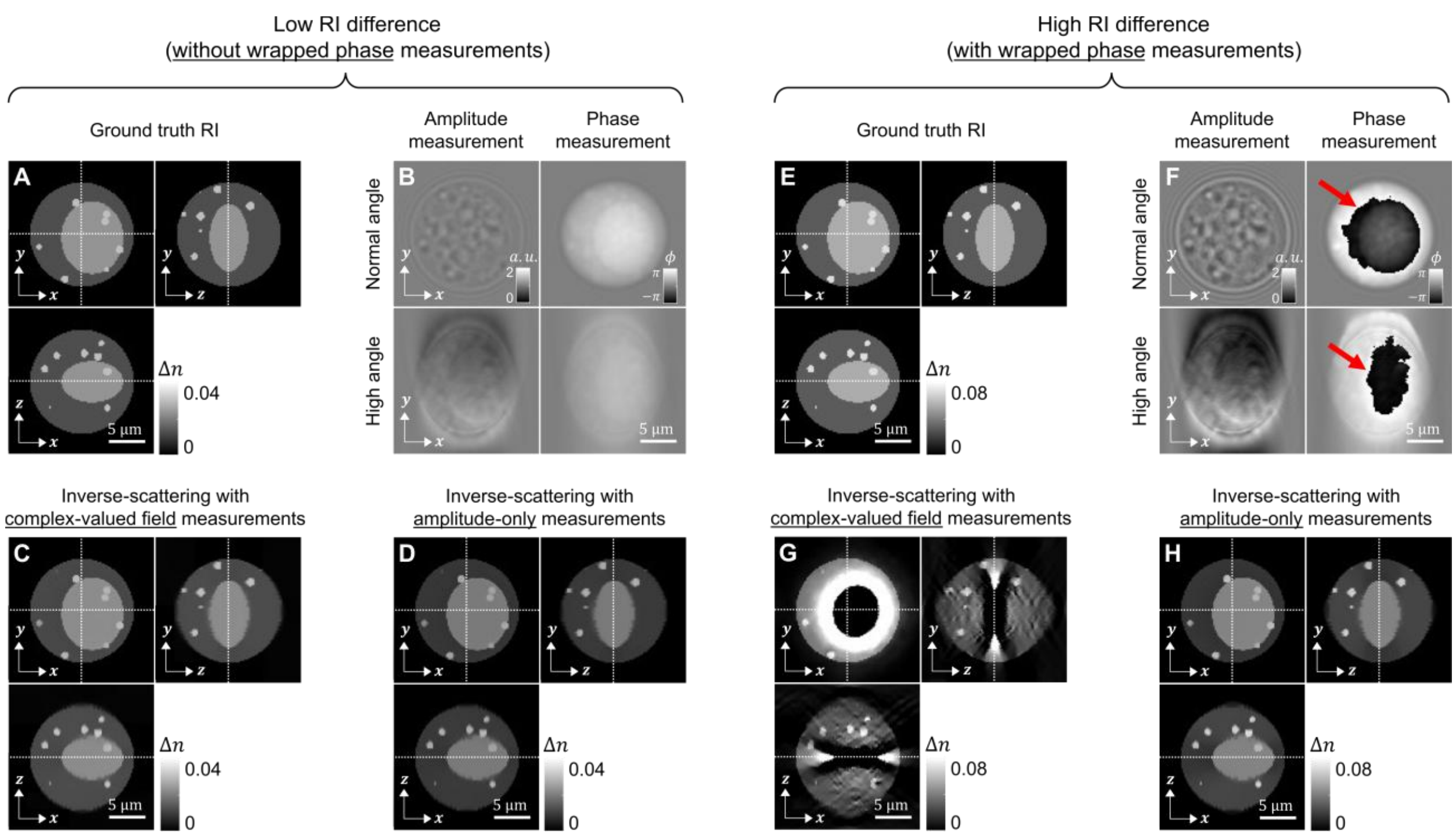

Figure S3. Simulation demonstrating the effects of phase-wrapping on 3D RI reconstruction using complex-field and amplitude-only MSBP. Two digital phantoms were simulated with identical geometry but different RI-contrast levels – low RI-contrast phantom does not induce phase wrapping while the high RI-contrast phantom does produce phase wrapping. **(A)** Ground-truth RI distribution is shown for the low RI-contrast phantom (Δn = 0.04). **(B)** Simulated amplitude and phase measurements under normal and high-angle illumination showed no phase-wraps. Successful RI reconstruction via MSBP was achieved both when **(C)** using complex-valued field measurements as well as **(D)** amplitude-only measurements. **(E)** Ground-truth RI distribution is shown for a high RI-contrast phantom (Δn = 0.08) with identical geometry and structures to (A). **(F)** Simulated amplitude and phase measurements under normal and high-angle illumination showed strong phase-wrapping, as indicated by red arrows in the phase images. **(G)** MSBP reconstruction using complex-valued field measurements showed severe artifacts that spatially coincide with the regions where phase wrapping occurred in the measurements. **(H)** MSBP reconstruction using amplitude-only measurements remained high-quality and successfully recovered the phantom's 3D RI distribution despite the phase-wrapped field measurements.

Notably, no phase-wrapping is present for this low RI-contrast phantom. Correspondingly, *both* complex-field MSBP reconstruction (**Figure S3C**) and amplitude-only MSBP reconstruction (**Figure S3D**) successfully reconstructed the phantom with high fidelity.

In contrast, **Figure S3(E-H)** show the RI distribution for a high RI-contrast phantom as well as amplitude/phase measurements captured at normal and high illumination angles, respectively. Notably for this phantom, strong phase wrapping is clearly visible in the measured phase images (**Figure S3F**). Correspondingly, reconstruction with the complex-field MSBP framework failed (**Figure S3G**), producing strong artifacts and physically incorrect structures. Importantly, these artifacts appear in spatial regions that closely coincide with the locations where severe phase wrapping occurs in the measurements, consistent with the failure patterns observed in our experimental results. Meanwhile, the amplitude-only MSBP reconstruction remained high-quality (**Figure S3H**).

Notably, the two cases depicted in **Figure S3(A-D)** versus **Figure S3(E-H)** differed only in the presence or absence of phase wrapping, while sharing identical object geometry, forward model, and simulation conditions. Thus, these results strongly demonstrate that phase-wrapping is the dominant mechanism responsible underlying the observed failures in complex-field MSBP reconstruction, rather than other external factors such as calibration errors, optical aberrations, noise statistics, etc. Moreover, these results establish that amplitude-only MSBP is inherently more robust for strongly scattering samples that induce strong phase-wrapping due to their pronounced RI heterogeneity.

Supplementary Note 5: Imaging MCF7 spheroids embedded in hydrogel media

In many biological imaging applications, 3D samples are embedded in specialized media such as hydrogels either during formation or for stabilization during imaging. These types of media help maintain sample structure and reduce movement, particularly when using modalities that require long acquisition times. However, such embedding may potentially introduce additional scattering and RI mismatches, making accurate reconstruction more challenging. To evaluate whether our proposed method remains effective under these conditions, we tested it on an MCF7 spheroid sample immersed in hydrogel.

Figure S4 shows the reconstruction results of a hydrogel-embedded MCF7 spheroid using amplitude-only MSBP inverse-scattering *with* defocus diversity. **Figure S4Aa** and **Figure S4Ab** present maximum intensity projections along the *xy* and *xz* planes, respectively, clearly revealing the internal cellular structures and the overall spheroid morphology. Cross-sectional views in **Figure S4Ba** and **Figure S4Bb**, corresponding to the z-planes marked in **Figure S4Ab**, provide detailed visualization of internal features at different depths. Similarly, **Figure S4Bc** and **Figure S4Bd**, extracted along the *y*-planes indicated in **Figure S4Aa**, highlight the consistent recovery of structures along the *xz* plane. **Figure S4Ca** and **Figure S4Cb** present zoomed-in views of the regions indicated by dashed lines in **Figure S4Ba** and **Figure S4Bb**, respectively, highlighting finer details. Individual cells are distinctly resolved within the spheroid, and subcellular features such as nuclei and lipids can be clearly differentiated based on their RI contrast. Furthermore, the spatial arrangement of these cells and their physical continuity can be observed, illustrating how they organize into a 3D spheroid structure.

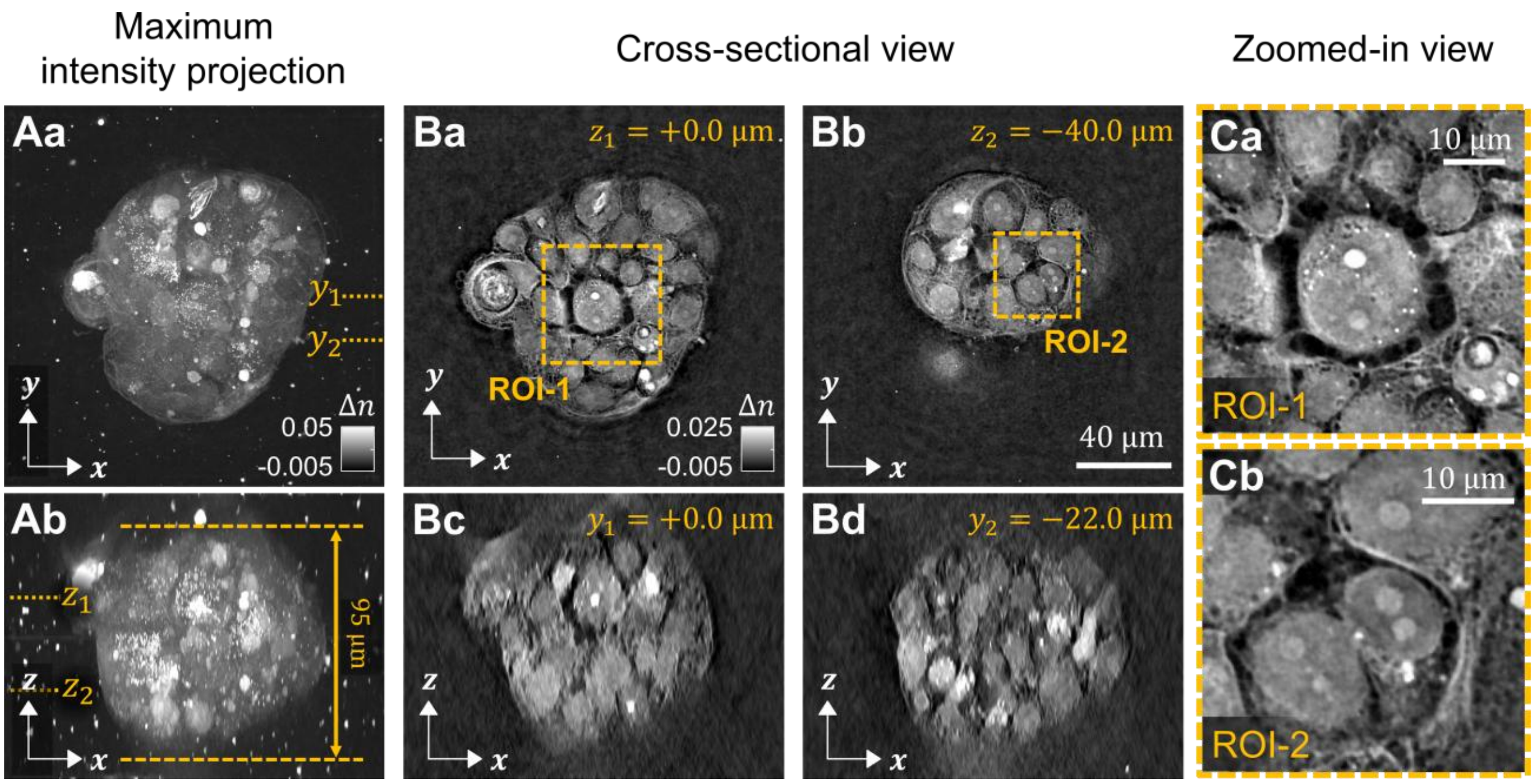

Figure S4. 3D RI reconstruction of an MCF7 spheroid embedded in hydrogel using amplitude-only MSBP inverse-scattering with defocus diversity. (Aa, Ab) Maximum intensity projections along the *xy* and *xz* planes. **(Ba, Bb)** *xy* cross-sections at depths $z = 0.0$ µm and $z = -40.0$ µm, corresponding to the slices indicated in (Ab). **(Bc, Bd)** *xz* cross-sections at heights $y = 0.0$ µm and $y = -22.0$ µm, as marked in (Aa). **(Ca, Cb)** Zoomed-in view of the regions outlined with dashed lines in (Ba) and (Bb), respectively. The reconstructed 3D RI volume reveals well-resolved internal cellular structures throughout the spheroid, highlighting the compatibility of the approach with hydrogel-embedded biological samples.

These results demonstrate that our method can robustly reconstruct samples embedded in hydrogel, preserving fine structural detail across multiple cross sections. This compatibility with hydrogel-based sample preparation protocols suggests that our approach is well-suited for a broad range of biological imaging scenarios and can be integrated with other common imaging workflows.

Supplementary Note 6: Comparison of inverse-scattering strategies with mutant C. elegans

In the main text (see **Fig. 5**), we compared how complex-valued and amplitude-only MSBP performed in reconstructing 3D RI in an organoid sample. Our observations indicated that performing amplitude-only inverse-scattering with computational defocusing yielded best results. We now conduct the same analysis with a different biological sample to see whether those results generalize.

In this demonstration, we used a genetically modified *C. elegans* mutant engineered to overproduce lipids, resulting in significantly higher scattering compared to the wild-type *C. elegans* (see **Figure S2** above). As in **Fig. 5**, we compared four reconstruction strategies: complex-valued MSBP with and without weak-scattering initialization, and amplitude-only MSBP with and without defocus diversity. The outcomes of these approaches are shown in **Figure S5**.

As shown in **Figure S5(Aa-Ac)**, field-based reconstruction with a constant initial guess (i.e., *without* weak-scattering initialization) resulted in prominent artifacts throughout the 3D volume. These artifacts were clearly observed in the axial cross-section (**Figure S5Aa**) and in the

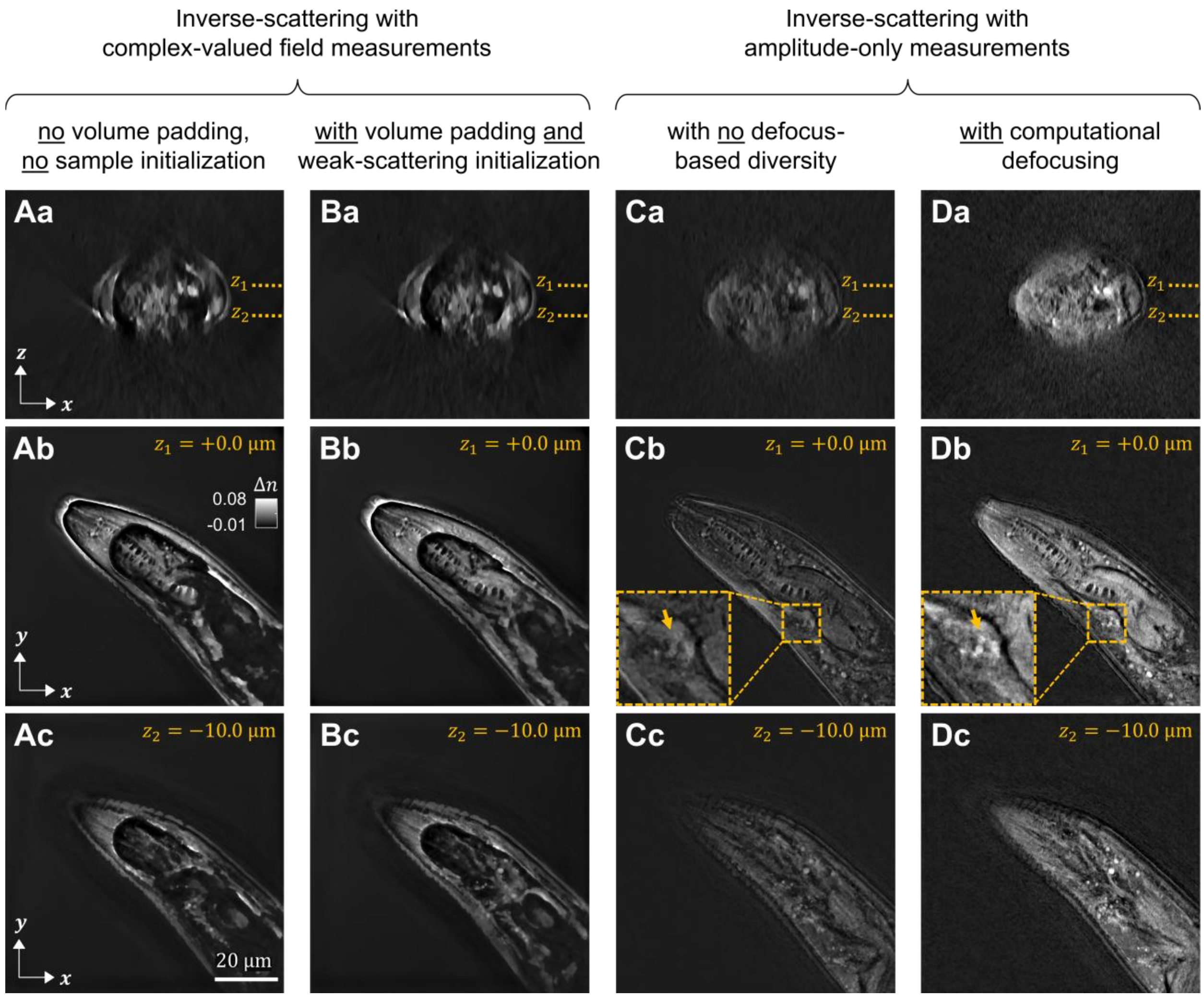

Figure S5. Comparison of MSBP-based inverse-scattering strategies for 3D RI reconstruction of a mutant *C. elegans* sample. 3D RI reconstruction is shown using complex-valued field measurements with **(Aa-Ac)** no weak-scattering initialization (i.e., RI initialized to $\Delta n = 0$), and **(Ba-Bc)** with weak-scattering initialization, where 3D RI is reconstructed via Fourier diffraction theorem and Rytov-based weak-scattering approximation(*56, 57*). 3D RI reconstruction is also shown when using amplitude-only measurements in the inverse-solver, **(Ca-Cc)** without and **(Da-Dc)** with additional defocused measurements to introduce depth-diversity into the measurement dataset. For each 3D RI reconstruction, **(Aa, Ba, Ca, Da)** the central axial cross-sectional planes and **(Ab, Ac)**, **(Bb, Bc)**, **(Cb, Cc)**, and **(Db, Dc)** two lateral cross-sectional planes, located at z = +0.0 µm and –10.0 µm are shown.

transverse *xy*-plane slices at two different depths (**Figure S5(Ab, Ac)**). In **Figure S5(Ba-Bc)**, we initialized the field-based MSBP solver using a 3D RI estimate derived from the Fourier diffraction theorem and Rytov weak-scattering approximation (*56, 57*). However, similar to before, the resulting RI reconstruction still exhibited sharp discontinuities and structural distortion. As with the organoid sample in **Fig. 5**, this suggests that weak-scattering initialization does not provide a sufficiently accurate initial guess for samples with this level of scattering complexity.

In **Figure S5(Ca-Cc)**, we see that amplitude-only inverse-scattering removes sharp discontinuities observed in the field-based inverse-scattering reconstructions. However, similar to earlier observations shown in the main manuscript's **Fig. 5**, the resulting RI distribution lacks contrast, and several internal structures remain unresolved (see zoomed-in views in **Figure S5Cb** and **Figure S5Db**). In contrast, incorporating defocused amplitude measurements significantly

improves reconstruction quality – the sharp discontinuities from field-based reconstruction are removed, and internal structures are recovered with higher and more uniform contrast compared to when not using defocused measurements for amplitude-only inverse-scattering.

These improvements mirror the results observed in the organoid sample in **Fig. 5**, as well as for the larger organoid and zebrafish embryo shown in **Fig. 7** and **Fig. 8** of the main text, respectively. This suggests the generalizability of these findings across diverse biological sample types.

Supplementary Note 7: Comparing RI reconstructions with and without defocus diversity

To more closely evaluate how defocus diversity improves 3D RI reconstruction using amplitude-based MSBP inverse-scattering, we assessed its impact across a range of samples with differing scattering strengths. Below, **Figure S6**(columns **(A)** – **(E)**) compares reconstruction results for five representative samples: a 100 μm scattering phantom, wild-type *C. elegans*, mutant *C. elegans*, intestinal organoids, and a zebrafish embryo. For each sample, subfigures **(a)** and **(b)** show *xz*- and *xy*-plane cross sections reconstructed *without* defocus diversity, while subfigures **(c)** and **(d)** show the corresponding cross sections *with* defocus diversity. White dashed lines mark the slicing positions for each view

Figure S6A and **Figure S6B** show results from the 100 μm scattering phantom and the wild-type *C. elegans*, respectively. In both cases, utilizing defocus diversity in the amplitude-based inverse solver yields modest improvements, specifically towards slightly enhancing contrast for rod-like features within the phantom and internal biological structures in the *C. elegans* (highlighted by yellow arrows in **Figure S6Ab** and zoomed-in view in **Figure S6Bb**). However, the improvements are minor and do not reveal entirely new features missing from the reconstructions without defocus diversity.

In contrast, incorporating defocus diversity yields substantially improved reconstructions for the mutant *C. elegans*, intestinal organoid, and zebrafish embryo, as shown in **Figure S6(C-E)**. For the mutant *C. elegans*, which is genetically engineered to overproduce lipids and thereby exhibit increased scattering, **Figure S6C** shows that internal anatomical features are clearly visualized only when defocus diversity is incorporated into the inverse-scattering framework. Without it, structural details remain obscured (see zoomed-in views in **Figure S6Cb** and **Figure S6Cd**). In **Figure S6D**, defocus diversity reduces edge artifacts along the sharp boundary of the intestinal organoid, which are otherwise prominent in reconstructions without it (see zoomed-in views in **Figure S6Db** and **Figure S6Dd**). Finally, **Figure S6E** demonstrates that in the zebrafish embryo, defocus diversity enables the recovery of entirely new internal features within regions that otherwise appear empty or unresolved.

Notably, the mutant *C. elegans*, intestinal organoid, and zebrafish embryo exhibit substantially higher scattering than either the phantoms or the wild-type *C. elegans*. While defocus diversity yields only modest improvements in RI reconstruction for the latter two, it produces markedly enhanced results in the more strongly scattering samples. This suggests that when multiple scattering effects are relatively modest, single-plane measurements may suffice for accurate reconstruction (*34, 35, 50, 60*). In contrast, for highly scattering samples, defocus diversity becomes essential to enhance contrast, reduce artifacts, and enable recovery of features otherwise lost in single-plane reconstructions. Interestingly, although the mutant *C. elegans* is only ~40 μm thick, which is less than half the thickness of the 100 μm phantom, the benefits of defocus

diversity are significantly greater. This underscores that the utility of defocus diversity is driven more by the scattering complexity of the sample than by its physical thickness.

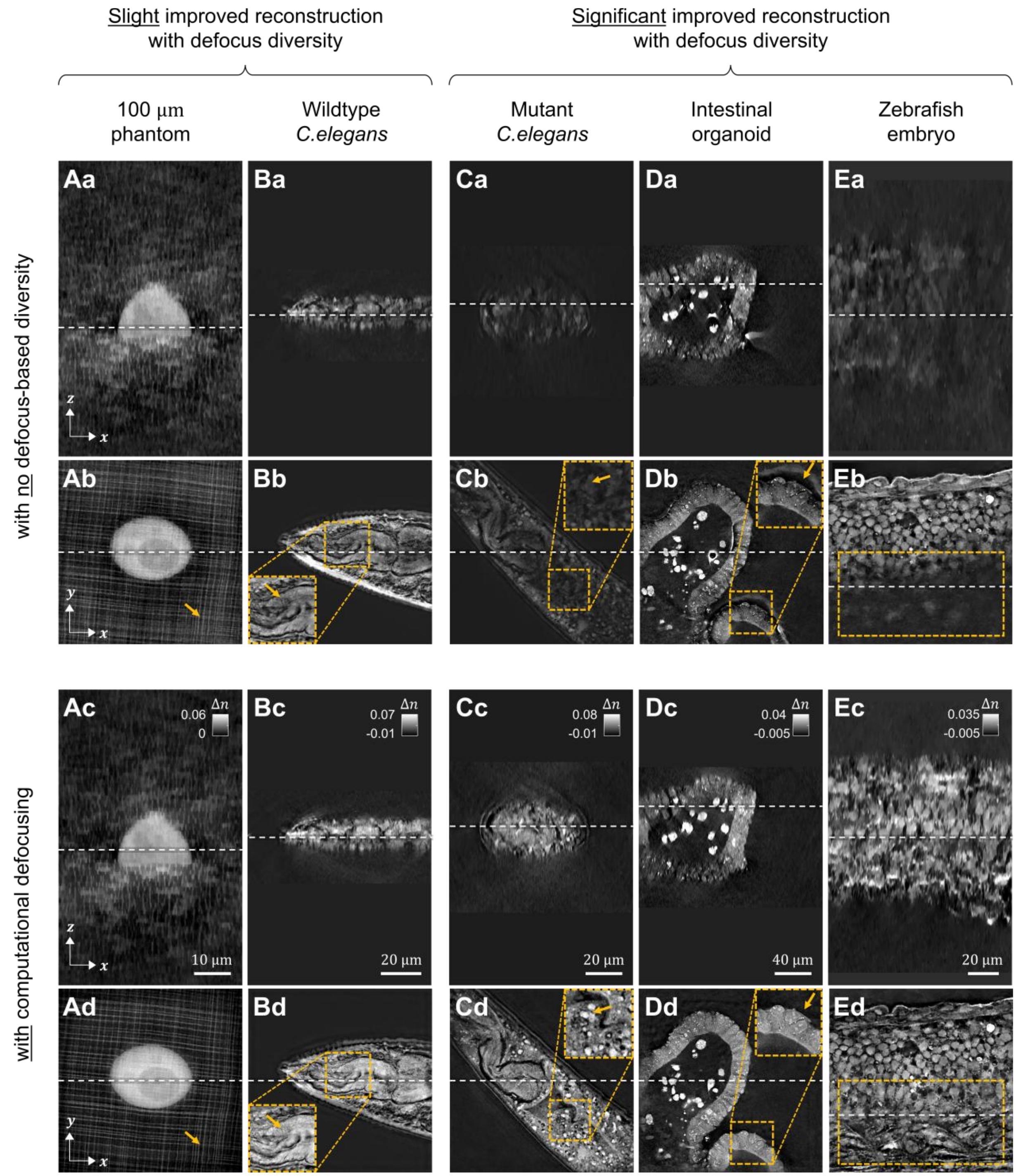

Figure S6. Comparison of reconstruction results with and without defocus diversity across different types of samples. Columns correspond to: **(A)** 100 µm scattering phantom, **(B)** wild-type *C. elegans*, **(C)** mutant *C. elegans*, **(D)** intestinal organoid, and **(E)** zebrafish embryo. For each sample, **(a)** and **(b)** show reconstruction results without defocus diversity, displaying the *xz*-plane and *xy*-plane cross sections, respectively, while **(c)** and **(d)** show the corresponding *xz*- and *xy*-plane results obtained with defocus diversity. The white dashed lines indicate the positions of the orthogonal slices shown in the corresponding cross-sectional views.

Supplementary Note 8: Comparing 3D RI reconstructions using MSBP versus weak-scattering model

To highlight the advantage of using MSBP-based inverse-scattering over the traditional weak-scattering approximation when reconstructing multiple-scattering samples, we provide a side-by-side comparison between reconstructions with the weak-scattering approximation and the MSBP-based inverse-scattering method.

Figure S7 shows representative results for four different samples: a 100 μm scattering phantom, a mutant *C. elegans*, intestinal organoids, and a zebrafish embryo. For each sample, we

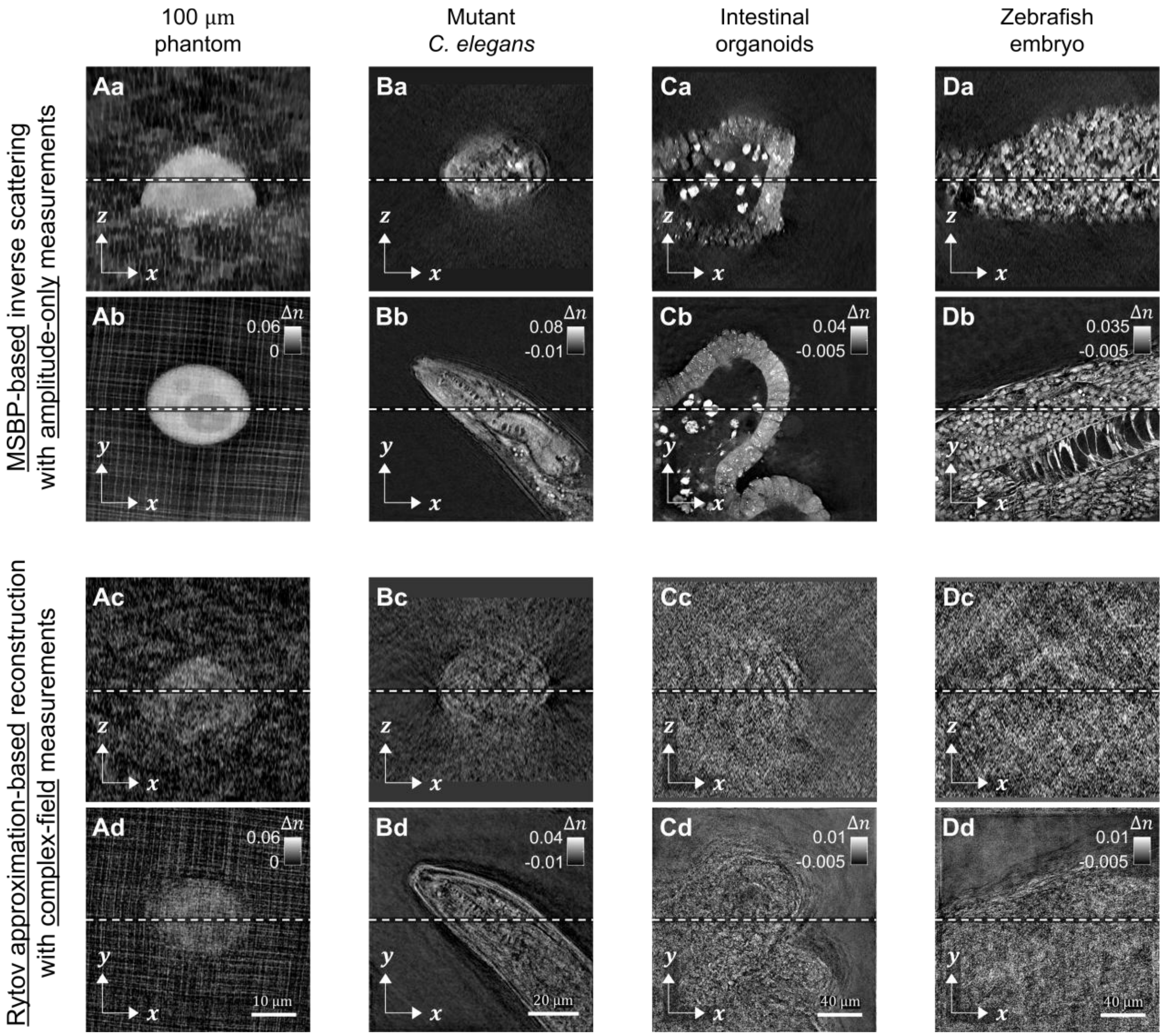

Figure S7. Comparison between 3D RI reconstructions with MSBP-based inverse-scattering versus weak-scattering linear reconstruction. (Aa-Ad) 100 μm scattering phantom, **(Ba-Bd)** mutant *C. elegans*, **(Ca-Cd)** intestinal organoids, and **(Da-Dd)** zebrafish embryo. For each sample, **(a, b)** show MSBP reconstructions (*xz* and *xy* cross-sections), and **(c, d)** show the corresponding linear reconstructions based on the Rytov approximation (*xz* and *xy* cross-sections). For the 100 μm scattering phantom, the *xz* views (Aa, Ac) are truncated along the z-axis for display. The dashed lines indicate the locations of the corresponding orthogonal slices. For the 100 μm scattering phantom, the weak-scattering linear reconstruction yields a noisy result with poorly resolved structural features. A similar behavior is observed for the scattering biological samples, whereas the MSBP reconstructions remain physically meaningful.

compare the MSBP-based inverse-scattering reconstruction with amplitude-only measurements and the linear reconstruction obtained using the Rytov approximation with complex-field measurements. The MSBP reconstructions are performed using the same reconstruction settings and parameters as in the main text. In these MSBP reconstructions, the scattering phantom is reconstructed without using defocused amplitude measurements, whereas the biological samples are reconstructed using defocused amplitude data, consistent with the strategies used in the main text.

For the 100 μm scattering phantom, the linear reconstruction based on the weak-scattering approximation fails to accurately recover the 3D RI distribution and yields a noisy reconstruction in which structural features are difficult to resolve (**Figure S7(Aa-Ad)**). A similar behavior is observed for scattering biological samples. The linear reconstructions fail to recover meaningful internal structure and are dominated by severe artifacts (**Figure S7(Bc–Dc, Bd–Dd)**), whereas the MSBP-based inverse-scattering reconstructions remain physically meaningful (**Figure S7(Ba–Da, Bb–Db)**).

Overall, this comparison demonstrates that the weak-scattering, linearized model is only valid in a limited regime and fails in scattering biological samples, whereas the MSBP-based approach remains applicable to these more challenging cases.

Supplementary Note 9: Limitations and challenges

While amplitude-based MSBP inverse-scattering incorporating defocused measurements demonstrates robust performance across a wide range of samples, it does not guarantee optimal imaging results. In the following section, we highlight limitations in this current method that provide insights into areas for future improvement.

Selection of defocus planes:

A key factor that influenced RI reconstruction quality was the choice of defocus planes. **Figure S8** compares reconstructions of an intestinal organoid sample using three different sets of virtually defocused amplitude-only measurements. In each case, three defocus planes, denoted as $z^{(1)}$, $z^{(2)}$ and $z^{(3)}$, were selected to computationally generate the defocused amplitude measurements. In the first configuration, shown in **Figure S8A** and **Figure S8B**, the defocus planes were set to $z^{(1)} = +44$ μm, $z^{(2)} = +40$ μm, and $z^{(3)} = +36$ μm, all physically above the conjugate imaging plane (z = 0 μm). The corresponding amplitude measurements under normal incidence illumination are shown in **Figure S8(Aa-Ac)**. **Figure S8Ba** displays the *xz*-plane cross-section of the reconstructed volume, with the selected defocus planes marked by red dashed lines. **Figure S8Bb** and **Figure S8Bc** show *xy* cross-sectional planes at different axial depths, as indicated by the yellow dashed lines in **Figure S8Ba**. This defocus configuration produced artifact-free reconstructions with clearly resolved internal structures.

In contrast, **Figure S8A** and **Figure S8D** show results using a symmetric defocus plane configuration centered around the imaging plane at $z^{(1)} = +36$ μm, $z^{(2)} = 0$ μm, and $z^{(3)} = -36$ μm. **Figure S8(Ca-Cc)** display representative amplitude measurements corresponding to these defocus positions, which were used to generate the reconstruction shown in **Figure S8(Da-Dc)**. As illustrated in the *xz* cross-section in **Figure S8Da**, where the defocus planes are marked by green dashed lines, this configuration introduced noticeable reconstruction artifacts, particularly near regions with sharp RI transitions. These artifacts were especially evident in the *xy*-plane cross-sections in **Figure S8Db** and **Figure S8Dc**, as indicated by the yellow arrows.

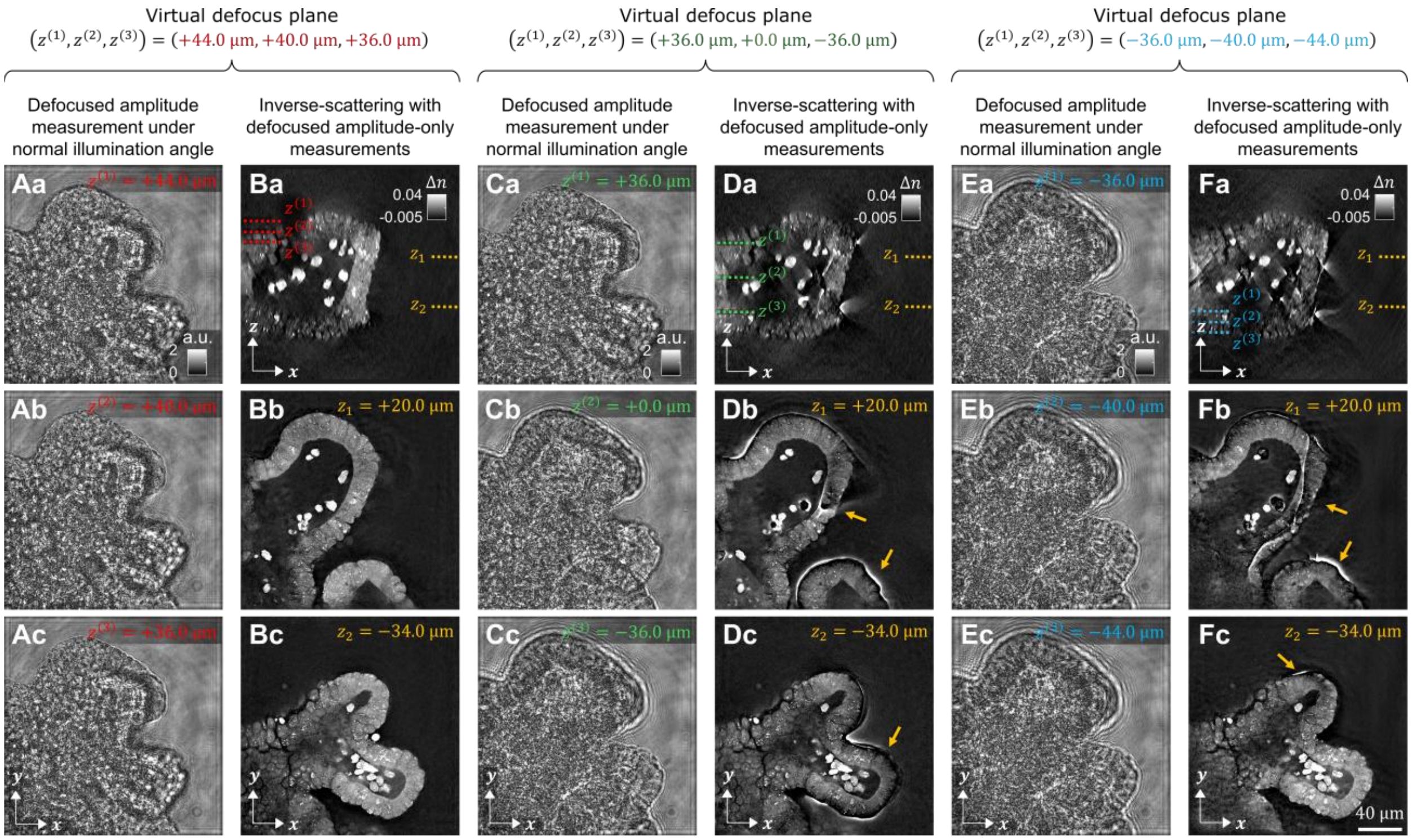

Figure S8. Comparison of amplitude-only MSBP reconstructions using different sets of virtually defocused measurements. (Aa-Ac) Representative amplitude images generated at defocus planes of +44 µm, +40 µm, and +36 µm with on-axis illumination. **(Ba-Bc)** Corresponding reconstruction results using the measurements in (Aa-Ac). **(Ba)** Central *xz* cross-section of the reconstructed volume, with defocus planes indicated by red dashed lines. Yellow dashed lines denote the z_1 and z_2 axial positions of the lateral xy layers shown in **(Bb)** and **(Bc)**, respectively. RI reconstruction clearly reveals internal structures with no artifacts. **(Ca-Cc)** Amplitude images generated at defocus planes of +36 µm, 0 µm, and –36 µm under the same on-axis illumination condition. **(Da-Dc)** Reconstruction results using (Ca-Cc) as input. **(Da)** *xz* cross-section through the center of the volume, with green dashed lines marking defocus positions. **(Db)** and **(Dc)** show lateral *xy* cross-sectional planes at depths indicated in (Da). Notable artifacts (indicated by yellow arrows) emerge near boundaries with sharp RI transitions. **(Ea-Ec)** Amplitude images generated at defocus planes of –36 µm, –40 µm, and –44 µm under the same on-axis illumination condition. **(Fa-Fc)** Reconstruction results using (Ea-Ec) as input. **(Fa)** *xz* cross-section through the center of the volume, with blue dashed lines marking defocus positions. **(Fb)** and **(Fc)** display lateral *xy* cross-sectional planes at depths marked in (Fa). Artifacts (indicated by yellow arrow) occur at sample boundary with abrupt refractive index changes, similar those in (Db) and (Dc).

Lastly, **Figure S8E** and **Figure S8F** show results obtained using defocus planes configuration located below the conjugated imaging plane, with defocus positions at $z^{(1)} = -36\ \mu m$, $z^{(2)} = -40\ \mu m$, and $z^{(3)} = -44\ \mu m$. **Figure S8(Ea-Ec)** present the representative amplitude images obtained at those defocus planes. The corresponding reconstruction result using those defocused amplitude measurements are shown in **Figure S8(Fa-Fc)**. In **Figure S8Fa**, an *xz* cross-section through the center of the reconstructed volume is displayed, with blue dashed lines indicating the positions of the defocus planes. Similar to the result in **Figure S8Da**, artifacts appear near sample boundaries with abrupt RI transitions. Lateral cross-sections extracted at the depths indicated in **Figure S8Fa**, shown in **Figure S8Fb** and **Figure S8Fc**, also exhibit artifacts (indicated by yellow arrow) resembling those observed in **Figure S8Db** and **Figure S8Dc.**

These observations highlight the critical importance of defocus plane selection in achieving high-fidelity reconstruction. For this organoid sample, using defocus planes clustered above the imaging plane effectively suppressed boundary-related artifacts. While the exact cause remains

uncertain, we hypothesize that the discrepancy arises from a mismatch between the MSBP model and optical effects within the organoid's lumen cavity. Specifically, the sharp RI transition due to the lumen cavity may act as a refractive lens, altering light propagation in ways not captured by the MSBP model, which assumes purely diffractive behavior. When defocus measurements are synthetically generated at axial positions spanning the lumen, unmodeled refractive effects may introduce errors, leading to the artifacts observed in **Figure S8(Da-Dc)** and **Figure S8(Fa-Fc)**. In contrast, generating defocus measurements from planes positioned above the cavity avoids these complications, allowing more accurate modeling via diffraction-only propagation and yielding cleaner reconstructions, as shown in **Figure S8(Ba-Bc)**.

Notably, our strategy to select defocus planes is currently a largely empirical process, guided by intuition rather than established principles. Current choices are based on heuristic reasoning and speculative insights, such as the hypothesis described above. A valuable direction for future work would be to rigorously test these assumptions and develop a systematic methodology to select defocus planes, so that accurate 3D RI reconstructions can be robustly achieved across diverse sample types.

Mismatch between sample scattering and MSBP assumptions:

While our results above highlight the importance of carefully selecting defocus planes, there remain cases where reliable 3D RI reconstruction could not be achieved even with extensive tuning of the defocus configuration. These limitations were especially apparent in three challenging samples: a thick intestinal organoid with a large lumen cavity greater than the ones in previous organoid samples, a MCF7 spheroid characterized by sparse internal structure and large intercellular gaps, and the yolk region in the zebrafish embryo sample that exhibited chaotic speckle scattering. **Figure S9** below shows raw intensity measurements from these samples, obtained under normal illumination, and corresponding RI reconstructions that clearly exhibit imaging artifacts. We speculate that the primary cause of these artifacts is a mismatch between the sample's scattering behavior and the underlying assumptions of the MSBP model, which assumes forward-propagating, diffraction-based scattering that gradually accumulates through the sample with no refraction. We describe this in more detail below.

The first case, shown in **Figure S9(A-C)**, presents the representative raw intensity measurement and reconstruction of an intestinal organoid with a total thickness exceeding 230 μm, thicker than prior samples. **Figure S9Aa** and **Figure S9Ab** show raw intensity measurement and the corresponding zoomed-in view of the region indicated by the dashed line in **Figure S9Aa**. **Figure S9Ba** and **Figure S9Bb** shows maximum intensity projections along the *xy* and *xz* planes, respectively. **Figure S9(Ca-Cd)** show *xy* and *xz* planes cross-sectional views at different *z* and *y* positions, corresponding to the regions indicated by dashed lines in **Figure S9Ba** and **Figure S9Bb**. Notably, for this particular sample, regardless of how virtual defocus planes were selected for this sample, strong artifact signals persisted specifically along the organoid boundaries (indicated by yellow arrows in **Figure S9Ba**). Moreover, the central region of the sample, which shows up as highly heterogeneous in the raw measurement with speckle-like scattering (**Figure S9Ab**), was poorly reconstructed and appeared blurred, without any visual clarity for any of the internal structural features. This blurred region is indicated with the dashed line in the lateral *xy* cross-section at z = +0.0 μm (see **Figure S9Ca**). Axial views in **Figure S9Bb** and **Figure S9Cd** further confirm both boundary artifacts and poor reconstruction quality throughout the volume.

The second case, shown in **Figure S9(D-F)**, involves an MCF7 spheroid with a thickness of approximately 135 μm, which is moderate relative to other samples tested. However, even with

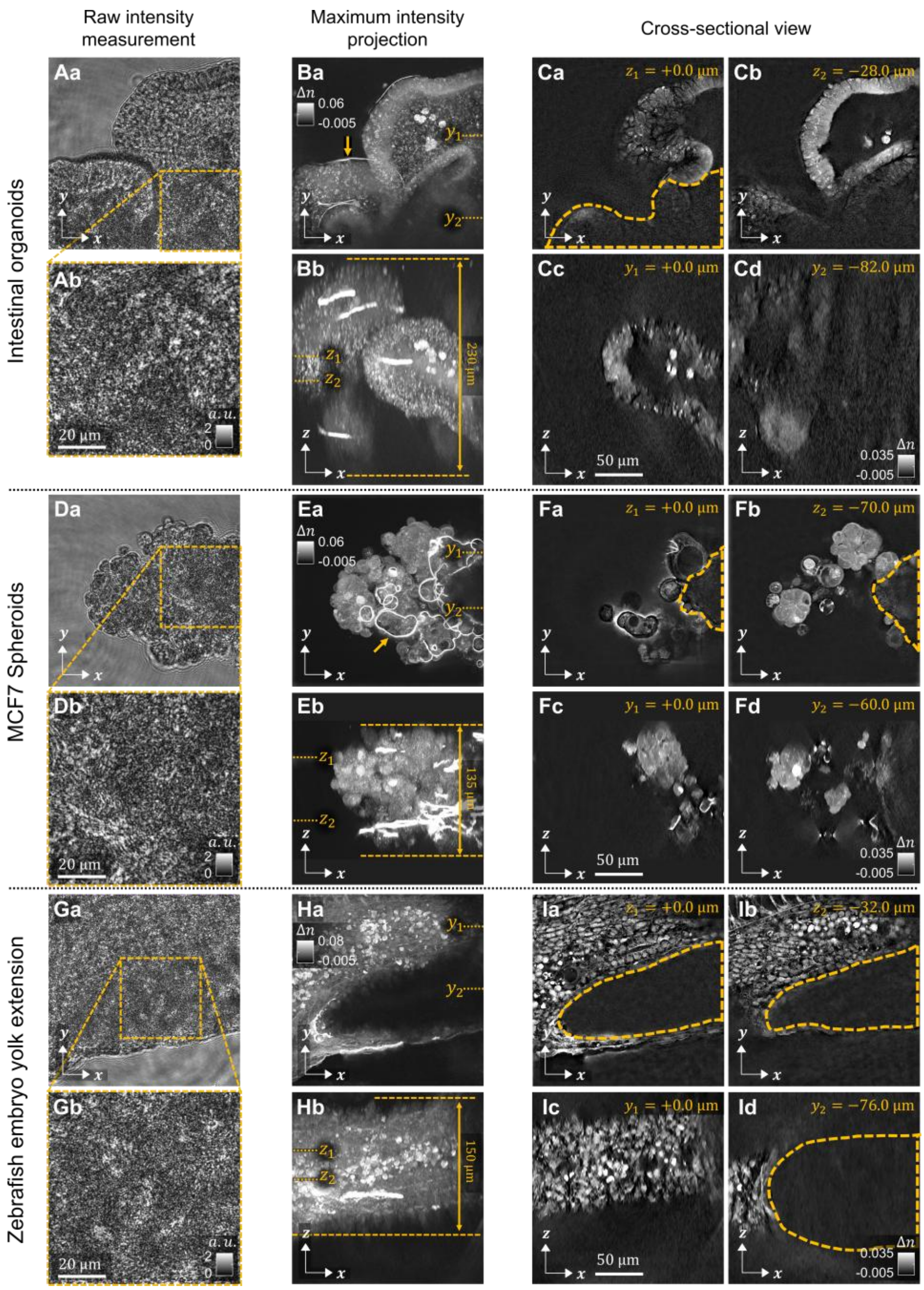

Figure S9. Reconstruction limitations in 3D samples. (A-C) Intestinal organoid results. **(Aa, Ab)** Representative raw intensity measurement obtained under normal illumination, and the corresponding zoomed-in view of the region indicated by the dashed line. **(Ba, Bb)** Maximum intensity projections along the *xy* and *xz* planes. Dashed lines in (Ba) and (Bb) indicate locations for the *xy* and *xz* cross-sections in (Ca-Cd). **(Ca, Cb)** *xy* cross-sections at $z = +0.0$ μm and $z = -28.0$ μm from planes marked in (Bb). **(Cc, Cd)** *xz* cross-sections at y = +0.0 μm and y = –82.0 μm from planes marked in (Ba). **(D-F)** MCF7 spheroid results. **(Da, Db)** Representative raw intensity measurement obtained under normal illumination, and the corresponding zoomed-in view of the region indicated by the dashed line. **(Ea, Eb)** Maximum intensity projections along the *xy* and *xz* planes. Dashed lines in (Ea) and (Eb) indicate positions for *xy* and *xz* cross-sections in (Fa-Fd). **(Fa, Fb)** *xy* cross-sections at $z = +0.0$ μm and $z = -70.0$ μm from (Eb); **(Fc, Fd)** *xz* cross-sections at $y = +0.0$ μm and $y = -60.0$ μm from (Ea). **(G-I)** Zebrafish embryo yolk extension results. **(Ga, Gb)** Representative raw intensity measurement obtained under normal illumination, and the corresponding zoomed-in view of the region indicated by the dashed line. **(Ha, Hb)** Maximum intensity projections along the *xy* and *xz* planes. Dashed lines in (Ha) and (Hb) indicate positions for *xy* and *xz* cross-sections in (Ia-Id). **(Ia, Ib)** *xy* cross-sections at $z = +0.0$ μm and $z = -32.0$ μm from (Hb); **(Ic, Id)** *xz* cross-sections at $y = +0.0$ μm and $y = -76.0$ μm from (Ha).

this moderate thickness, reliable reconstruction could not be achieved. Importantly, unlike the MCF7 spheroid presented earlier in **Figure S4**, which was embedded in hydrogel, this sample was immersed in PBS, resulting in a looser structural arrangement. The absence of hydrogel support led to large intercellular gaps and minimal internal continuity. This sparse and disconnected organization contrasted sharply with the densely packed morphology of the other samples explored in this work, and presented an additional challenge for robust RI reconstruction. Due to the loose cellular packing in this spheroid, the sample exhibited numerous large RI discontinuities at the various sample-to-media interfaces, violating the core assumption of the MSBP model that scattering arises from gradual, layer-by-layer accumulation with minimal refractive effects. The structural sparsity extended throughout the entire volume, making it impossible to identify any virtual focal plane free of significant gaps. As a result, defocus diversity failed to suppress reconstruction artifacts, unlike in the denser organoid samples shown previously. This sparsity is readily visible in the cross-sectional views (**Figure S9(Fa-Fd)**), where large internal voids that disrupt continuity are readily apparent. Consequently, the reconstructed 3D RI volume exhibited prominent artifacts stemming from these sharp RI transitions, as seen in the maximum intensity projections in **Figure S9Ea** and **Figure S9Eb**, with yellow arrows indicating the most affected regions. **Figure S9Da** and **Figure S9Db** present the raw intensity measurement and its zoomed-in view, showing regions with speckle-like patterns similar to those observed in the intestinal organoid in **Figure S9Aa**. As indicated by the dashed lines in **Figure S9Fa** and **Figure S9Fb**, reconstruction of these regions are noticeably blurred.

The final case, shown in **Figure S9(G-I)**, corresponds to the yolk extension region of the zebrafish embryo sample presented in **Fig. 8** of the main text, where reconstruction was unsuccessful. **Figure S9Ga** and **Figure S9Gb** display the raw measurement image and a zoomed-in view of the region containing the yolk extension, respectively, revealing a fully evolved speckle pattern. **Figure S9Ha** and **Figure S9Hb** show the maximum intensity projections of the reconstructed volume in the *xy* and *xz* planes, respectively, with the cross-sectional views corresponding to the dashed lines in each projection presented in **Figure S9(Ia-Id)**. The dashed region in the *xy* cross section corresponds to the yolk extension, which could not be reconstructed and appears simply as an empty cavity in the otherwise structurally-dense zebrafish reconstruction.

These results underscore that both fully-evolved speckle scattering and internal structural gaps with strong RI-mismatch interfaces can limit the effectiveness of MSBP-based reconstruction. In such cases, neither boundary artifacts nor central blurring could be resolved, suggesting a fundamental mismatch between the sample's scattering behavior and the assumptions of the MSBP model. Future work may benefit from exploring alternative scattering models that better accommodate these complexities, offering a promising path to improving 3D RI imaging in similarly challenging specimens.

Supplementary Note 10: Measurement diversity combining angular illumination and sample defocus

In the main manuscript, we showed that measurements collected both at different illumination angles as well as sample defocus positions substantially improved RI reconstruction quality, especially in strongly scattering regimes. Notably, similar observations were made in previous works, where multi-angle and multi-plane (i.e., sample-defocus) measurements were both used to improve RI reconstructions in weak-scattering samples(*23, 54*). Our empirical results suggest that this strategy also yields improvements in the multiple-scattering regime.

However, a natural argument may be that reconstruction improvements result from simply using more measurements in the inverse solver, rather than from the diversity of measurements within the combined angular-illumination and sample-defocus dataset. To confirm whether reconstruction improvements arose from *measurement diversity* versus *measurement count*, we conducted the experiment described below.

We returned to the zebrafish embryo tail-tip dataset, for which a total of 500 angled-illumination measurements were originally acquired. Because this number was already more than sufficient for stable reconstruction, we intentionally restricted ourselves to using only 250 measurements with equal angular spacing to create a more challenging and informative test case. We then compared two reconstruction strategies that both used exactly 250 measurements in total. In the first strategy, we use 250 angular illuminations at a single focal plane (i.e., without defocus diversity). In the second strategy, we use only 125 angular illuminations and computationally generate one additional defocus plane per illumination angle by digitally propagating each complex field measurement by a distance of 8 μm relative to the imaging plane. This resulted in a total of $125 \times 2 = 250$ measurements with mixed angular and defocus diversity.

Figure S10(Aa-Ac) show the reconstruction obtained using 250 angular illuminations *without* defocus diversity. **Figure S10(Ba-Bc)** show the reconstruction obtained using 125 angular illuminations each with two defocus planes (250 total measurements). Although both reconstructions used exactly the same number of measurements in the MSBP amplitude-based inverse problem (i.e., Eq. 4, main text), the reconstruction incorporating both angular and defocus

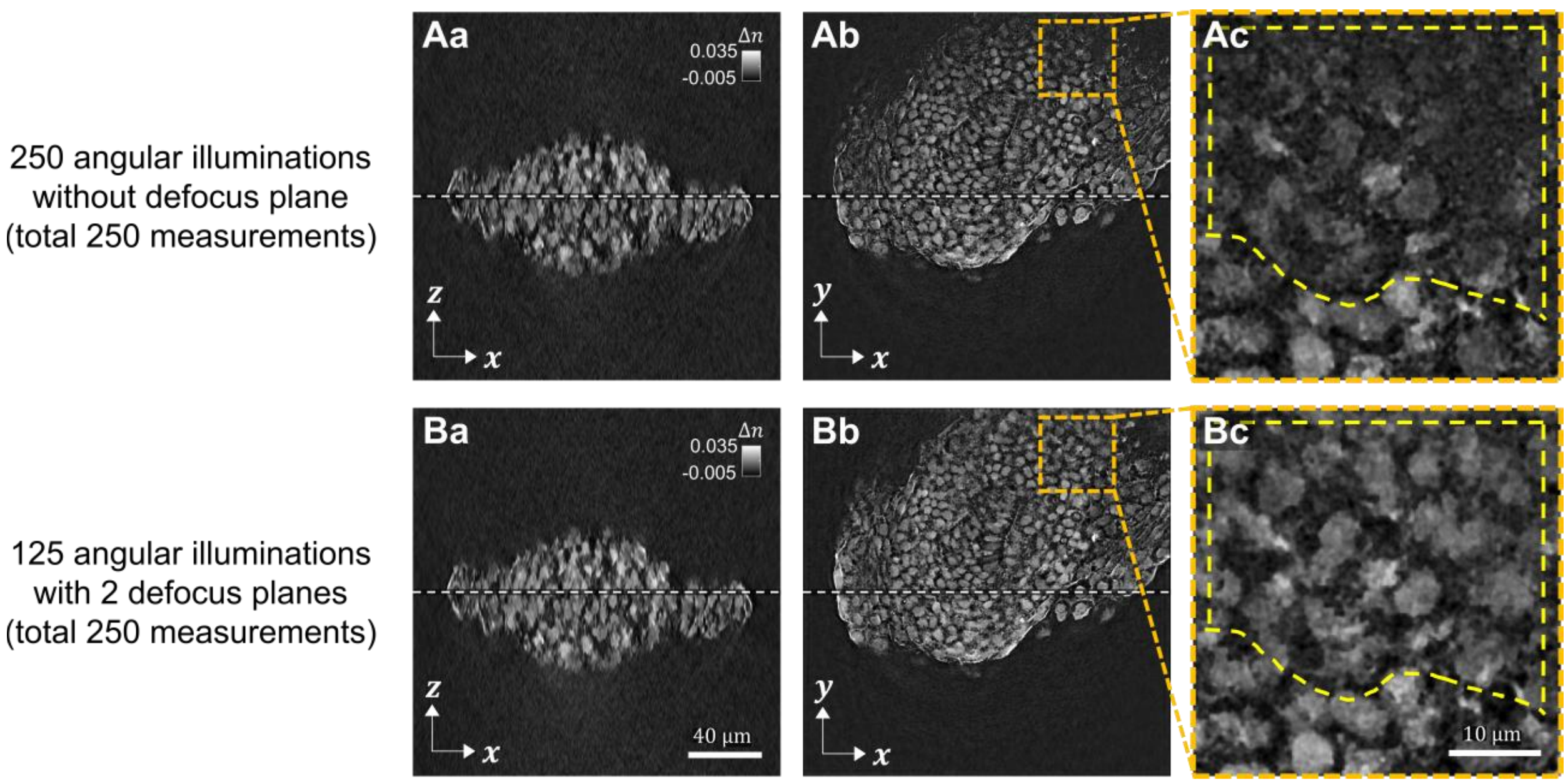

Figure S10. Demonstrating improvements in image reconstruction from combining angled-illumination and sample-defocus measurements. (Aa–Ac) Reconstruction using 250 angular illuminations at a single focal plane (i.e., no defocus diversity). **(Aa)** *xz* cross-section, **(Ab)** *xy* cross-section. The dashed lines in (Aa) and (Ab) indicate the locations of the corresponding orthogonal slices. **(Ac)** Magnified view of the highlighted region. **(Ba-Bc)** Reconstruction using 125 angular illuminations, where two defocus planes for each illumination angle are generated computationally by digitally propagating each complex field measurement, resulting in 250 total measurements. **(Ba)** *xz* cross-section, **(Bb)** *xy* cross-section. The dashed lines in (Ba) and (Bb) indicate the locations of the corresponding orthogonal slices. **(Bc)** Magnified view of the highlighted region. Although both reconstructions in (Aa-Ac) and (Ba-Bc) use the same total number of measurements, the increased measurement-diversity in (Ba-Bc) exhibits improved image quality and structural fidelity compared to the angular-only strategy (Aa-Ac), particularly in the highlighted regions with yellow dashed line.

diversity exhibited visibly improved image quality and structural fidelity, particularly in the dashed-yellow regions (**Figure S10Ac** and **Figure S10Bc**). This result provides experimental support for the observations in the main text that quality of MSBP reconstructions can be significantly improved specifically with the measurement diversity contained within angular and defocus measurements.

Supplementary Note 11: Comparison of optimization algorithms for MSBP reconstruction

Solving the MSBP inverse-scattering problem is inherently a nonconvex optimization problem. As reported in the main text, our results indicated that amplitude-only measurements yielded better reconstruction quality than complex-valued fields. However, since we primarily employed stochastic gradient descent to solve the inverse problem, and given that alternative optimization strategies are known to achieve superior convergence for other types of nonconvex problems (*117-119*), an important question is whether the observed reconstruction behaviors were intrinsic to the choice of cost function, or whether they were a consequence of the optimizer itself. To investigate this, we performed a systematic comparison of various optimization strategies using 40 μm scattering phantom.

In addition to our analytic-gradient implementation with stochastic gradient descent with momentum (SGDM), we developed an independent reconstruction pipeline based on PyTorch's automatic differentiation (autograd) (*120*), which served both to validate our analytic-gradient implementation and enabled systematic comparison across optimizers. Specifically, we benchmarked SGDM, root-mean-square-propagation (RMSProp), and Adam under identical reconstruction settings for both the amplitude-only and complex-field cost functions. These three optimizers were chosen to cover a broad and interpretable range of optimization strategies. SGDM served as the first-order baseline, updating parameters uniformly using gradient history with momentum acceleration. RMSProp added per-parameter adaptivity by scaling updates according to recent gradient magnitudes, and Adam combined both of these properties by incorporating adaptivity and momentum together. As a result, the three optimizers collectively captured the key characteristics of modern first-order gradient-based methods.

Reconstruction results with the amplitude-only cost function are shown in **Figure S11(A-D)**. Specifically, **Figure S11A** shows the *xz* and *xy* results obtained using our analytic-gradient implementation with SGDM, while **Figure S11(B-D)** show the analogous reconstructions obtained using PyTorch autograd with SGDM, RMSProp, and Adam, respectively. The corresponding convergence curves for the amplitude-only cost function are shown in **Figure S11I**. For the amplitude-only formulation, all tested optimizers converged to reconstructions that were largely consistent with those obtained using our analytic-gradient implementation. Apart from Adam, which exhibited somewhat larger fluctuations in the cost curve and slightly less stable convergence, all optimizers produced closely matched cost curves and reconstructed 3D RI distributions that matched the phantom's known structure.

Analogous results for the complex-field cost function are shown in **Figure S11(E-H)** and **Figure S11J**. Across all optimizers, reconstructions were again largely consistent with our analytic-gradient baseline. Notably however, all optimizers working to minimize the complex-field cost function consistently produced strong artifacts surrounding the cell phantom within the scattering cube, which was a region known to exhibit strong phase wraps as previously shown in **Figure S2**. The fact that these artifacts appeared regardless of optimizer or gradient computation strategy

strongly suggests that such reconstruction failures are intrinsic to the complex-field cost function itself for scattering samples rather than a consequence of any particular optimization approach.

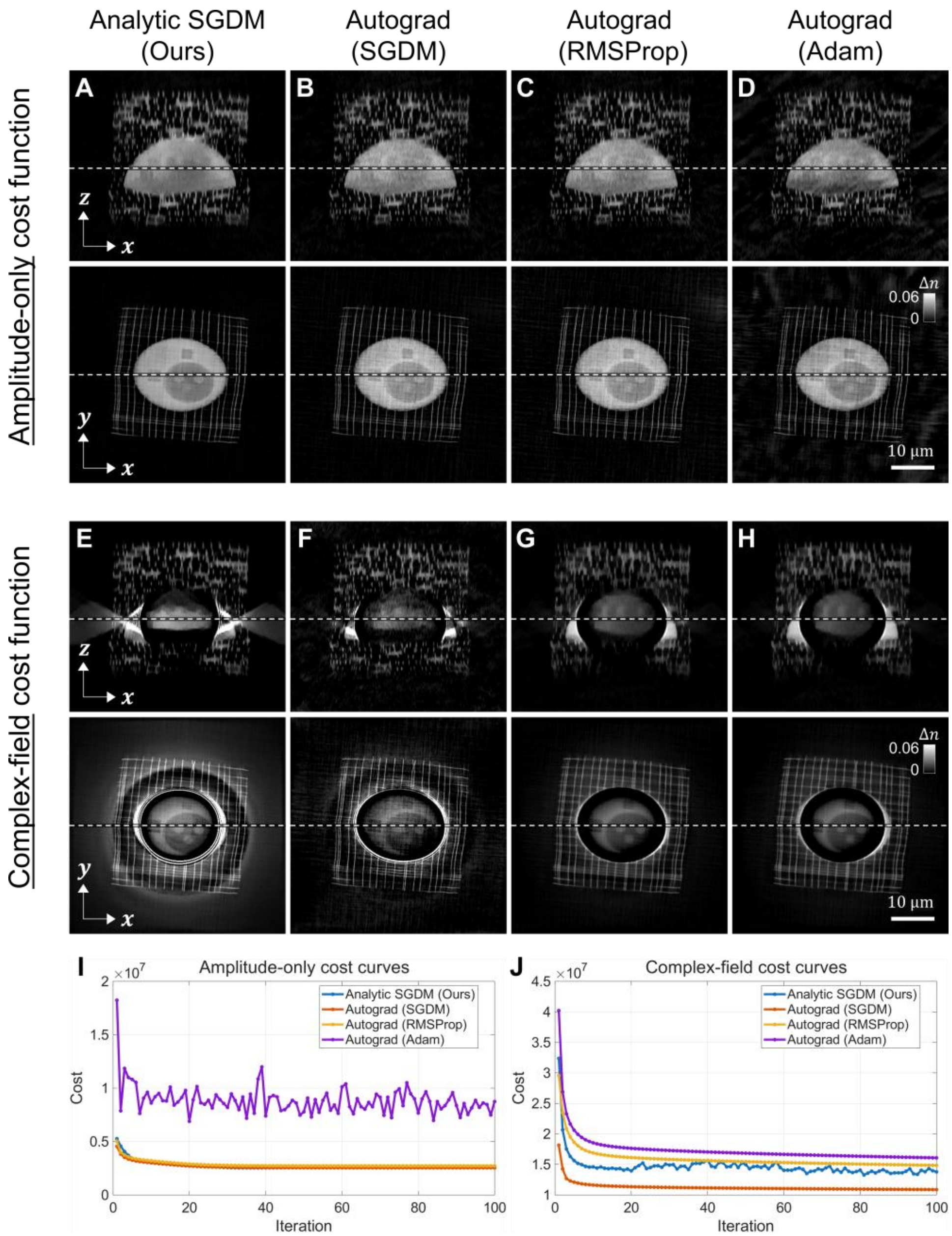

Figure S11. Comparison of optimization algorithms for amplitude-only and complex-field MSBP reconstruction. (A-D) MSBP reconstructions using the amplitude-only cost function with different optimization strategies: (**A**, this work) analytic gradient with SGDM, **(B)** PyTorch autograd with SGDM, **(C)** autograd with RMSProp, and **(D)** autograd with Adam. Dashed lines overlaid on the images indicate the locations of the *xz* and *xy* cross-sections. **(E-H)** MSBP reconstructions using the complex-field cost function with the same set of optimizers and gradient implementations: (**E**, this work) analytic gradient with SGDM, **(F)** autograd with SGDM, **(G)** autograd with RMSProp, and **(H)** autograd with Adam. **(I)** Convergence curves for the amplitude-only cost function corresponding to the optimizers shown in (A-D). **(J)** Convergence curves for the complex-field cost function corresponding to the optimizers shown in (E-H). For the amplitude-only formulation, all optimizers converge to 3D reconstructions closely matched to the sample's known structure. In contrast, for the complex-field formulation, all optimizers produce reconstructions dominated by artifacts, indicating that these failures are intrinsic to the complex-field cost function rather than specific to the choice of optimizer or gradient implementation.

Supplementary Note 12: Summary of datasets and reconstruction conditions

We summarize here information related to the datasets and reconstruction settings used for each experimental result presented in the main manuscript and this Supplementary document. For each sample presented in this work, **Table S1** below lists the data size (number of illumination angles, reconstruction volume, reconstructed data size), numerical aperture of objective lens, maximum illumination angle, reconstruction parameters (step size, regularization parameter, iteration number, and defocus planes), raw data acquisition time and computational time per iteration. Since some samples were larger than a single field of view, multiple fields of view were often used to reconstruct the entire sample. Therefore, the information presented in the table corresponds to a single field of view, with the last column additionally indicating the number of fields of view used

**Parameters**		**Sample type**							
		40μm phantom **(Figure 4)**	60μm phantom **(Figure 4)**	80μm phantom **(Figure 4)**	100μm phantom **(Figure 4)**	Mutant *C. elegans* **(Figure 6)**	Intestinal organoid **(Figure 7)**	Zebrafish embryo **(Figure 8)**	MCF7 Spheroid **(Figure S5)**
Per one field of view	Number of illumination angles	500	500	500	500	250	600	500	300
	Numerical aperture (NA) of objective lens	1.42	1.42	1.42	1.42	1.42	0.8	0.8	0.8
	Maximum illumination angle [°]	59.65	62.66	62.65	62.86	53.32	35.19	35.08	34.65
	Reconstruction volume (W×H×D) [μm × μm × μm]	$55 \times 55 \times 44$	$55 \times 55 \times 60$	$55 \times 55 \times 84$	$55 \times 55 \times 100$	$99 \times 99 \times 72$	$198 \times 198 \times 192$	$198 \times 198 \times 200$	$158 \times 158 \times 120$
	Reconstructed data size [MB]	100	138	186	221	417	1,075	1,087	862
	Step size for gradient descent	1×10^{-4}	1×10^{-4}	1×10^{-4}	1×10^{-4}	2×10^{-4}	1×10^{-4}	1×10^{-4}	1×10^{-4}
	Regularization parameter	1.5×10^{-3}	1.5×10^{-3}	1.5×10^{-3}	1.5×10^{-3}	1.5×10^{-3}	2×10^{-3}	1.5×10^{-3}	1.5×10^{-3}
	Defocus planes [μm]	0	0	0	0	+9,+6,+3	+44,+40,+36	+8,0,-8	+24,+16,+8
	Data acquisition time [sec]	110.5	110.5	110.5	110.5	55.3	132.6	110.5	66.3
	Iteration number	40	40	40	40	60	40	50	30
	Computation time (1 iteration) [min]	2.11*	2.69*	3.56*	4.10*	2.59**	11.54**	10.27**	3.29**
Total fields of view		1	1	1	1	13	4	11	1

Table S1. **Summary of reconstruction parameters, dataset sizes, and computation details for experimental results presented in this study.** For each dataset, we report the number of illumination angles used for reconstruction, numerical aperture of the objective lens, maximum illumination angle, the reconstruction volume dimensions (width × height × depth), the data size of the reconstructed volume, the step size and regularization parameter used, the total number of iterations, the defocus positions in cases where defocus diversity was applied, data acquisition time for acquiring raw measurements for one field of view, the computation time per iteration (* Computation performed on a local computer equipped with an NVIDIA GeForce RTX 3090Ti and MATLAB R2022b, ** Computation performed on the Lonestar7 system at the TACC with an NVIDIA A100 and MATLAB R2023b.), and total number of field of view used for the full sample reconstruction.

for the full sample reconstruction. Data acquisition time for a single field of view accounted for the sensor exposure time, trigger time, and any residual delay times (e.g., from data transfer or from galvo mirrors stabilizing after movement due to inertia). Time required to physically move the sample between fields of view is not included. Computational reconstructed time is reported separately for 40, 60, 80, and 100 μm scattering phantoms, which were reconstructed on a local computer equipped with an NVIDIA GeForce RTX 3090Ti and MATLAB R2022b, and other biological samples, which were reconstructed on the Lonestar7 system at the Texas Advanced Computing Center (TACC) with an NVIDIA A100 and MATLAB R2023b. This information is intended to provide a clear reference for the scale of the problem, parameter choices, and computational resources required for the reported reconstructions, thereby facilitating reproducibility and enabling comparison with other reconstruction frameworks.

Video captions

Supplementary Video 1:	Cross-sectional views are shown through different z-planes of a *daf-2(e1370)* mutant *C. elegans* worm. Image contrast is fully label-free, stemming from the worm's intrinsic refractive index distribution.
Supplementary Video 2:	Zoomed-in views highlight sub-cellular anatomical features with high resolution and contrast in a *daf-2(e1370)* mutant *C. elegans* worm. 3D tomographic render illustrates the volumetric distribution of the worm's refractive index.
Supplementary Video 3:	Cross-sectional views are shown through different z-planes of an intestinal organoid sample. Image contrast is fully label-free, stemming from the organoid's intrinsic refractive index distribution.
Supplementary Video 4:	Zoomed-in views highlight sub-cellular anatomical features with high resolution and contrast in an intestinal organoid sample. 3D tomographic render illustrates the volumetric distribution of the organoid's refractive index.
Supplementary Video 5:	Cross-sectional views are shown through different z-planes of a zebrafish embryo sample. Image contrast is fully label-free, stemming from the embryo's intrinsic refractive index distribution.
Supplementary Video 6:	Zoomed-in views highlight sub-cellular anatomical features with high resolution and contrast in a zebrafish embryo sample. 3D tomographic render illustrates the volumetric distribution of the embryo's refractive index.

REFERENCES

1. F. Helmchen, W. Denk, Deep tissue two-photon microscopy. *Nat. Methods* **2**, 932–940 (2005).

2. L. Streich, J. C. Boffi, L. Wang, K. Alhalaseh, M. Barbieri, R. Rehm, S. Deivasigamani, C. T. Gross, A. Agarwal, R. Prevedel, High-resolution structural and functional deep brain imaging using adaptive optics three-photon microscopy. *Nat. Methods* **18**, 1253–1258 (2021).

3. D. M. Scully, I. V. Larina, Mouse embryo phenotyping with optical coherence tomography. *Front. Cell Dev. Biol.* **10**, 1000237 (2022).

4. S. A. Boppart, M. E. Brezinski, B. E. Bouma, G. J. Tearney, J. G. Fujimoto, Investigation of developing embryonic morphology using optical coherence tomography. *Dev. Biol.* **177**, 54–63 (1996).

5. J. G. Fujimoto, C. Pitris, S. A. Boppart, M. E. Brezinski, Optical coherence tomography: An emerging technology for biomedical imaging and optical biopsy. *Neoplasia* **2**, 9–25 (2000).

6. M. D. Cahalan, I. Parker, S. H. Wei, M. J. Miller, Two-photon tissue imaging: Seeing the immune system in a fresh light. *Nat. Rev. Immunol.* **2**, 872–880 (2002).

7. J. Wiskin, B. Malik, D. Borup, N. Pirshafiey, J. Klock, Full wave 3D inverse scattering transmission ultrasound tomography in the presence of high contrast. *Sci. Rep.* **10**, 20166 (2020).

8. L. M. Neira, B. D. Van Veen, S. C. Hagness, High-resolution microwave breast imaging using a 3-D inverse scattering algorithm with a variable-strength spatial prior constraint. *IEEE Trans. Antennas Propag.* **65**, 6002–6014 (2017).

9. D. Ren, C. Ophus, M. Chen, L. Waller, A multiple scattering algorithm for three dimensional phase contrast atomic electron tomography. *Ultramicroscopy* **208**, 112860 (2020).

10. K. Wang, W. Sun, C. T. Richie, B. K. Harvey, E. Betzig, N. Ji, Direct wavefront sensing for high-resolution in vivo imaging in scattering tissue. *Nat. Commun.* **6**, 7276 (2015).

11. T.-L. Liu, S. Upadhyayula, D. E. Milkie, V. Singh, K. Wang, I. A. Swinburne, K. R. Mosaliganti, Z. M. Collins, T. W. Hiscock, J. Shea, Observing the cell in its native state: Imaging subcellular dynamics in multicellular organisms. *Science* **360**, eaaq1392 (2018).

12. P. Zdankowski, D. McGloin, J. R. Swedlow, Full volume super-resolution imaging of thick mitotic spindle using 3D AO STED microscope. *Biomed. Opt. Express* **10**, 1999–2009 (2019).

13. W. Choi, C. Fang-Yen, K. Badizadegan, S. Oh, N. Lue, R. R. Dasari, M. S. Feld, Tomographic phase microscopy. *Nat. Methods* **4**, 717–719 (2007).

14. K. Kim, V. R. Gade, T. V. Kurzchalia, J. Guck, Quantitative imaging of *Caenorhabditis elegans* dauer larvae during cryptobiotic transition. *Biophys. J.* **121**, 1219–1229 (2022).

15. J. Li, Q. Chen, J. Zhang, Z. Zhang, Y. Zhang, C. Zuo, Optical diffraction tomography microscopy with transport of intensity equation using a light-emitting diode array. *Opt. Lasers Eng.* **95**, 26–34 (2017).

16. Y. Baek, Y. Park, Intensity-based holographic imaging via space-domain Kramers–Kronig relations. *Nat. Photonics* **15**, 354–360 (2021).

17. Y. Sung, W. Choi, C. Fang-Yen, K. Badizadegan, R. R. Dasari, M. S. Feld, Optical diffraction tomography for high resolution live cell imaging. *Opt. Express* **17**, 266–277 (2009).

18. J. Kostencka, T. Kozacki, A. Kuś, B. Kemper, M. Kujawińska, Holographic tomography with scanning of illumination: Space-domain reconstruction for spatially invariant accuracy. *Biomed. Opt. Express* **7**, 4086–4101 (2016).

19. M. Hörmann, F. V. Camargo, N. F. van Hulst, G. Cerullo, M. Liebel, Ultrabroadband optical diffraction tomography. *ACS Photonics* **11**, 3680–3687 (2024).

20. K. Lee, S. Shin, Z. Yaqoob, P. T. So, Y. Park, Low-coherent optical diffraction tomography by angle-scanning illumination. *J. Biophotonics* **12**, e201800289 (2019).

21. L. Tian, L. Waller, 3D intensity and phase imaging from light field measurements in an LED array microscope. *Optica* **2**, 104–111 (2015).

22. R. Horstmeyer, J. Chung, X. Ou, G. Zheng, C. Yang, Diffraction tomography with Fourier ptychography. *Optica* **3**, 827–835 (2016).

23. J. Li, N. Zhou, J. Sun, S. Zhou, Z. Bai, L. Lu, Q. Chen, C. Zuo, Transport of intensity diffraction tomography with non-interferometric synthetic aperture for three-dimensional label-free microscopy. *Light Sci. Appl.* **11**, 154 (2022).

24. J. Li, W. Shao, T. Li, X. Mou, MPIS-IDTM: Multi-plane assisted iterative solution against scattering in intensity diffraction tomographic microscopy. *Opt. Laser Technol.* **184**, 112544 (2025).

25. Y. Sung, W. Choi, N. Lue, R. R. Dasari, Z. Yaqoob, Stain-free quantification of chromosomes in live cells using regularized tomographic phase microscopy. *PLOS ONE* **7**, e49502 (2012).

26. S. Shin, K. Kim, J. Yoon, Y. Park, Active illumination using a digital micromirror device for quantitative phase imaging. *Opt. Lett.* **40**, 5407–5410 (2015).

27. H. Hugonnet, M. Lee, Y. Park, Optimizing illumination in three-dimensional deconvolution microscopy for accurate refractive index tomography. *Opt. Express* **29**, 6293–6301 (2021).

28. M. Chen, L. Tian, L. Waller, 3D differential phase contrast microscopy. *Biomed. Opt. Express* **7**, 3940–3950 (2016).

29. T. H. Nguyen, M. E. Kandel, M. Rubessa, M. B. Wheeler, G. Popescu, Gradient light interference microscopy for 3D imaging of unlabeled specimens. *Nat. Commun.* **8**, 210 (2017).

30. G. Kim, H. Hugonnet, K. Kim, J.-H. Lee, S. S. Lee, J. Ha, C. Lee, H. Park, K.-J. Yoon, Y. Shin, Holotomography. *Nat. Rev. Methods Primers* **4**, 51 (2024).

31. J. A. Fleck, J. Morris, M. Feit, Time-dependent propagation of high energy laser beams through the atmosphere. *Appl. Phys.* **10**, 129–160 (1976).

32. M. Feit, J. Fleck Jr, Light propagation in graded-index optical fibers. *Appl. Optics* **17**, 3990–3998 (1978).

33. K.-H. Brenner, W. Singer, Light propagation through microlenses: A new simulation method. *Appl. Optics* **32**, 4984–4988 (1993).

34. S. Chowdhury, M. Chen, R. Eckert, D. Ren, F. Wu, N. Repina, L. Waller, High-resolution 3D refractive index microscopy of multiple-scattering samples from intensity images. *Optica* **6**, 1211–1219 (2019).

35. J. Zhu, H. Wang, L. Tian, High-fidelity intensity diffraction tomography with a non-paraxial multiple-scattering model. *Opt. Express* **30**, 32808–32821 (2022).

36. W. Pierré, M. Briard, G. Godefroy, S. Desissaire, M. Dhellemmes, E. D. Llano, C. Loeuillet, P. F. Ray, C. Arnoult, C. Allier, CNN-based approach for 3D artifact correction of intensity diffraction tomography images. *Opt. Express* **32**, 34825–34837 (2024).

37. S. Yang, J. Kim, M. E. Swartz, J. K. Eberhart, S. Chowdhury, DMD and microlens array as a switchable module for illumination angle scanning in optical diffraction tomography. *Biomed. Opt. Express* **15**, 5932–5946 (2024).

38. G. Osnabrugge, S. Leedumrongwatthanakun, I. M. Vellekoop, A convergent Born series for solving the inhomogeneous Helmholtz equation in arbitrarily large media. *J. Comput. Phys.* **322**, 113–124 (2016).

39. M. Lee, H. Hugonnet, Y. Park, Inverse problem solver for multiple light scattering using modified Born series. *Optica* **9**, 177–182 (2022).

40. T. Li, J. Zhu, Y. Shen, L. Tian, Reflection-mode diffraction tomography of multiple-scattering samples on a reflective substrate from intensity images. *Optica* **12**, 406–417 (2025).

41. O. Yasuhiko, K. Takeuchi, H. Yamada, Y. Ueda, Multiple-scattering suppressive refractive index tomography for the label-free quantitative assessment of multicellular spheroids. *Biomed. Opt. Express* **13**, 962–979 (2022).

42. O. Yasuhiko, K. Takeuchi, In-silico clearing approach for deep refractive index tomography by partial reconstruction and wave-backpropagation. *Light Sci. Appl.* **12**, 101 (2023).

43. O. Yasuhiko, K. Takeuchi, Bidirectional in-silico clearing approach for deep refractive-index tomography using a sparsely sampled transmission matrix. *Biomed. Opt. Express* **15**, 5296–5313 (2024).

44. H.-Y. Liu, D. Liu, H. Mansour, P. T. Boufounos, L. Waller, U. S. Kamilov, SEAGLE: Sparsity-driven image reconstruction under multiple scattering. *IEEE Trans. Comput. Imaging* **4**, 73–86 (2018).

45. E. Soubies, T.-A. Pham, M. Unser, Efficient inversion of multiple-scattering model for optical diffraction tomography. *Opt. Express* **25**, 21786–21800 (2017).

46. T.-a. Pham, E. Soubies, A. Ayoub, J. Lim, D. Psaltis, M. Unser, Three-dimensional optical diffraction tomography with Lippmann-Schwinger model. *IEEE Trans. Comput. Imaging* **6**, 727–738 (2020).

47. R. Liu, S. Cheng, Y. He, X. Fan, Z. Lin, Z. Luo, On the convergence of learning-based iterative methods for nonconvex inverse problems. *IEEE Trans. Pattern Anal. Mach. Intell.* **42**, 3027–3039 (2019).

48. M. Danilova, P. Dvurechensky, A. Gasnikov, E. Gorbunov, S. Guminov, D. Kamzolov, I. Shibaev, Recent theoretical advances in non-convex optimization. arXiv:2012.06188 [math.OC] (2020).

49. V. Cambareri, L. Jacques, Through the haze: A non-convex approach to blind gain calibration for linear random sensing models. *Inf. Inference* **8**, 205–271 (2019).

50. U. S. Kamilov, I. N. Papadopoulos, M. H. Shoreh, A. Goy, C. Vonesch, M. Unser, D. Psaltis, Optical tomographic image reconstruction based on beam propagation and sparse regularization. *IEEE Trans. Comput. Imaging* **2**, 59–70 (2016).

51. A. M. Maiden, M. J. Humphry, J. M. Rodenburg, Ptychographic transmission microscopy in three dimensions using a multi-slice approach. *J. Opt. Soc. Am. A* **29**, 1606–1614 (2012).

52. S. Song, J. Kim, T. Moon, B. Seong, W. Kim, C.-H. Yoo, J.-K. Choi, C. Joo, Polarization-sensitive intensity diffraction tomography. *Light Sci. Appl.* **12**, 124 (2023).

53. U. S. Kamilov, I. N. Papadopoulos, M. H. Shoreh, A. Goy, C. Vonesch, M. Unser, D. Psaltis, Learning approach to optical tomography. *Optica* **2**, 517–522 (2015).

54. S. Zhou, J. Li, J. Sun, N. Zhou, H. Ullah, Z. Bai, Q. Chen, C. Zuo, Transport-of-intensity Fourier ptychographic diffraction tomography: Defying the matched illumination condition. *Optica* **9**, 1362–1373 (2022).

55. J. W. Goodman, *Introduction to Fourier Optics* (Roberts and Company Publishers, 2005).

56. E. Wolf, Three-dimensional structure determination of semi-transparent objects from holographic data. *Optics Commun.* **1**, 153–156 (1969).

57. M. Born, E. Wolf, *Principles of Optics: Electromagnetic Theory of Propagation, Interference and Diffraction of Light* (Elsevier, 2013).

58. A. Devaney, Inverse-scattering theory within the Rytov approximation. *Opt. Lett.* **6**, 374–376 (1981).

59. M. K. Kim, Principles and techniques of digital holographic microscopy. *SPIE Rev.* **1**, 018005 (2010).

60. J. Lim, A. B. Ayoub, E. E. Antoine, D. Psaltis, High-fidelity optical diffraction tomography of multiple scattering samples. *Light Sci. Appl.* **8**, 82 (2019).

61. W. Krauze, A. Kuś, M. Ziemczonok, M. Haimowitz, S. Chowdhury, M. Kujawińska, 3D scattering microphantom sample to assess quantitative accuracy in tomographic phase microscopy techniques. *Sci. Rep.* **12**, 19586 (2022).

62. M. J. Girard, N. G. Strouthidis, C. R. Ethier, J. M. Mari, Shadow removal and contrast enhancement in optical coherence tomography images of the human optic nerve head. *Invest. Ophthalmol. Vis. Sci.* **52**, 7738–7748 (2011).

63. E. J. O'Rourke, A. A. Soukas, C. E. Carr, G. Ruvkun, C. elegans major fats are stored in vesicles distinct from lysosome-related organelles. *Cell Metab.* **10**, 430–435 (2009).

64. C. Castro, J. Krumsiek, N. J. Lehrbach, S. A. Murfitt, E. A. Miska, J. L. Griffin, A study of *Caenorhabditis elegans* DAF-2 mutants by metabolomics and differential correlation networks. *Mol. Biosyst.* **9**, 1632–1642 (2013).

65. E. W. Hislop, W. J. Tipping, K. Faulds, D. Graham, Label-free imaging of lipid droplets in prostate cells using stimulated Raman scattering microscopy and multivariate analysis. *Anal. Chem.* **94**, 8899–8908 (2022).

66. L. R. Lapierre, S. Gelino, A. Meléndez, M. Hansen, Autophagy and lipid metabolism coordinately modulate life span in germline-less *C. elegans*. *Curr. Biol.* **21**, 1507–1514 (2011).

67. K. Papsdorf, J. W. Miklas, A. Hosseini, M. Cabruja, C. S. Morrow, M. Savini, Y. Yu, C. G. Silva-García, N. R. Haseley, L. M. Murphy, Lipid droplets and peroxisomes are co-regulated to drive lifespan extension in response to mono-unsaturated fatty acids. *Nat. Cell Biol.* **25**, 672–684 (2023).

68. R. Cong, C. Lu, X. Li, Z. Xu, Y. Wang, S. Sun, Tumor organoids in cancer medicine: From model systems to natural compound screening. *Pharm. Biol.* **63**, 89–109 (2025).

69. J.-O. Ryu, Y.-J. Seong, E. Lee, S.-Y. Lee, D. W. Lee, Applications and research trends in organoid based infectious disease models. *Sci. Rep.* **15**, 25185 (2025).

70. A. C. Rios, H. Clevers, Imaging organoids: A bright future ahead. *Nat. Methods* **15**, 24–26 (2018).

71. G. de Medeiros, R. Ortiz, P. Strnad, A. Boni, F. Moos, N. Repina, L. Challet Meylan, F. Maurer, P. Liberali, Multiscale light-sheet organoid imaging framework. *Nat. Commun.* **13**, 4864 (2022).

72. M. Held, I. Santeramo, B. Wilm, P. Murray, R. Lévy, Ex vivo live cell tracking in kidney organoids using light sheet fluorescence microscopy. *PLOS ONE* **13**, e0199918 (2018).

73. T. F. Bullen, S. Forrest, F. Campbell, A. R. Dodson, M. J. Hershman, D. M. Pritchard, J. R. Turner, M. H. Montrose, A. J. Watson, Characterization of epithelial cell shedding from human small intestine. *Lab. Invest.* **86**, 1052–1063 (2006).

74. A. L. Cruz, E. d. A. Barreto, N. P. Fazolini, J. P. Viola, P. T. Bozza, Lipid droplets: Platforms with multiple functions in cancer hallmarks. *Cell Death Dis.* **11**, 105 (2020).

75. F. G. Blankenberg, In vivo detection of apoptosis. *J. Nucl. Med.* **49**, 81S–95S (2008).

76. J. Boren, K. Brindle, Apoptosis-induced mitochondrial dysfunction causes cytoplasmic lipid droplet formation. *Cell Death Differ.* **19**, 1561–1570 (2012).

77. S. Kang, P. Kang, S. Jeong, Y. Kwon, T. D. Yang, J. H. Hong, M. Kim, K. D. Song, J. H. Park, J. H. Lee, High-resolution adaptive optical imaging within thick scattering media using closed-loop accumulation of single scattering. *Nat. Commun.* **8**, 2157 (2017).

78. R. Horstmeyer, H. Ruan, C. Yang, Guidestar-assisted wavefront-shaping methods for focusing light into biological tissue. *Nat. Photonics* **9**, 563–571 (2015).

79. I. N. Papadopoulos, J.-S. Jouhanneau, J. F. Poulet, B. Judkewitz, Scattering compensation by focus scanning holographic aberration probing (F-SHARP). *Nat. Photonics* **11**, 116–123 (2017).

80. H. Yu, J. Park, K. Lee, J. Yoon, K. Kim, S. Lee, Y. Park, Recent advances in wavefront shaping techniques for biomedical applications. *Curr. Appl. Phys.* **15**, 632–641 (2015).

81. Z. Yu, H. Li, T. Zhong, J.-H. Park, S. Cheng, C. M. Woo, Q. Zhao, J. Yao, Y. Zhou, X. Huang, Wavefront shaping: A versatile tool to conquer multiple scattering in multidisciplinary fields. *Int. J. Hydrogen Energ.* **3**, 100292 (2022).

82. Z. Kam, B. Hanser, M. Gustafsson, D. Agard, J. Sedat, Computational adaptive optics for live three-dimensional biological imaging. *Proc. Natl. Acad. Sci. U.S.A.* **98**, 3790–3795 (2001).

83. Q. Zhang, Q. Hu, C. Berlage, P. Kner, B. Judkewitz, M. Booth, N. Ji, Adaptive optics for optical microscopy. *Biomed. Opt. Express* **14**, 1732–1756 (2023).

84. M. Booth, D. Andrade, D. Burke, B. Patton, M. Zurauskas, Aberrations and adaptive optics in super-resolution microscopy. *Microscopy* **64**, 251–261 (2015).

85. R. Lin, E. T. Kipreos, J. Zhu, C. H. Khang, P. Kner, Subcellular three-dimensional imaging deep through multicellular thick samples by structured illumination microscopy and adaptive optics. *Nat. Commun.* **12**, 3148 (2021).

86. C. Ounkomol, S. Seshamani, M. M. Maleckar, F. Collman, G. R. Johnson, Label-free prediction of three-dimensional fluorescence images from transmitted-light microscopy. *Nat. Methods* **15**, 917–920 (2018).

87. B. Bai, X. Yang, Y. Li, Y. Zhang, N. Pillar, A. Ozcan, Deep learning-enabled virtual histological staining of biological samples. *Light Sci. Appl.* **12**, 57 (2023).

88. H. M. L. Faulkner, J. M. Rodenburg, Movable aperture lensless transmission microscopy: A novel phase retrieval algorithm. *Phys. Rev. Lett.* **93**, 023903 (2004).

89. M. Guizar-Sicairos, J. R. Fienup, Phase retrieval with transverse translation diversity: A nonlinear optimization approach. *Opt. Express* **16**, 7264–7278 (2008).

90. C. Guo, S. Jiang, P. Song, T. Wang, X. Shao, Z. Zhang, G. Zheng, Quantitative multi-height phase retrieval via a coded image sensor. *Biomed. Opt. Express* **12**, 7173–7184 (2021).

91. J. W. Goodman, *Statistical Optics* (John Wiley & Sons, 2015).

92. M. Arigovindan, J. W. Sedat, D. A. Agard, Effect of depth dependent spherical aberrations in 3D structured illumination microscopy. *Opt. Express* **20**, 6527–6541 (2012).

93. K. Matsushima, T. Shimobaba, Band-limited angular spectrum method for numerical simulation of free-space propagation in far and near fields. *Opt. Express* **17**, 19662–19673 (2009).

94. L. Tian, X. Li, K. Ramchandran, L. Waller, Multiplexed coded illumination for Fourier ptychography with an LED array microscope. *Biomed. Opt. Express* **5**, 2376–2389 (2014).

95. M. R. Kellman, E. Bostan, N. A. Repina, L. Waller, Physics-based learned design: Optimized coded-illumination for quantitative phase imaging. *IEEE Trans. Comput. Imaging* **5**, 344–353 (2019).

96. R. Shi, L. Kong, Evaluating structured-illumination patterns in optimizing optical-sectioning of HiLo microscopy. *J. Phys. D Appl. Phys.* **54**, 414001 (2021).

97. Y. Fan, J. Sun, Q. Chen, X. Pan, L. Tian, C. Zuo, Optimal illumination scheme for isotropic quantitative differential phase contrast microscopy. *Photonics Res.* **7**, 890–904 (2019).

98. S. Desissaire, M. Ziemczonok, T. Cantat-Moltrecht, A. Kuś, G. Godefroy, L. Hervé, C. Paviolo, W. Krauze, C. Allier, O. Mandula, Bio-inspired 3d-printed phantom: Encoding cellular heterogeneity for characterization of quantitative phase imaging. *Measurement* **247**, 116765 (2025).

99. M. Ziemczonok, S. Desissaire, J. Neri, A. Kuś, L. Herve, C. Fiche, G. Godefroy, M. Fackeure, D. Sery, W. Krauze, Tailored 3D microphantoms: An essential tool for quantitative phase tomography analysis of organoids. *Biocybern. Biomed. Eng.* **45**, 247–257 (2025).

100. K. Monakhova, J. Yurtsever, G. Kuo, N. Antipa, K. Yanny, L. Waller, Learned reconstructions for practical mask-based lensless imaging. *Opt. Express* **27**, 28075–28090 (2019).

101. X. Wu, Z. Wu, S. C. Shanmugavel, H. Z. Yu, Y. Zhu, Physics-informed neural network for phase imaging based on transport of intensity equation. *Opt. Express* **30**, 43398–43416 (2022).

102. W. Pierré, L. Hervé, C. Paviolo, O. Mandula, V. Remondiere, S. Morales, S. Grudinin, P. F. Ray, M. Dhellemmes, C. Arnoult, 3D time-lapse imaging of a mouse embryo using intensity diffraction tomography embedded inside a deep learning framework. *Appl. Optics* **61**, 3337–3348 (2022).

103. A. Matlock, J. Zhu, L. Tian, Multiple-scattering simulator-trained neural network for intensity diffraction tomography. *Opt. Express* **31**, 4094–4107 (2023).

104. X. Ma, W. Xiao, F. Pan, Optical tomographic reconstruction based on multi-slice wave propagation method. *Opt. Express* **25**, 22595–22607 (2017).

105. H. Hugonnet, C. Oh, J. Park, Y. Park, Pupil phase series: A fast, accurate, and energy-conserving model for forward and inverse light scattering in thick biological samples. *Opt. Express* **33**, 34255–34266 (2025).

106. H. Wang, W. Zhang, D. Ladika, H. Yu, D. Gailevičius, H. Wang, C. F. Pan, P. N. S. Nair, Y. Ke, T. Mori, Two-photon polymerization lithography for optics and photonics: Fundamentals, materials, technologies, and applications. *Adv. Funct. Mater.* **33**, 2214211 (2023).

107. A. Žukauskas, I. Matulaitienė, D. Paipulas, G. Niaura, M. Malinauskas, R. Gadonas, Tuning the refractive index in 3D direct laser writing lithography: Towards GRIN microoptics. *Laser Photonics Rev.* **9**, 706–712 (2015).

108. A.-I. Bunea, N. del Castillo Iniesta, A. Droumpali, A. E. Wetzel, E. Engay, R. Taboryski, , Micro 3D printing by two-photon polymerization: Configurations and parameters for the nanoscribe system. *Micron* **1**, 164–180 2021).

109. M. Ziemczonok, A. Kuś, P. Wasylczyk, M. Kujawińska, 3D-printed biological cell phantom for testing 3D quantitative phase imaging systems. *Sci. Rep.* **9**, 18872 (2019).

110. K. Moshksayan, A. Harihara, S. Mondal, E. Hegarty, T. Atherly, D. K. Sahoo, A. E. Jergens, J. P. Mochel, K. Allenspach, J. Zoldan, OrganoidChip facilitates hydrogel-free immobilization for fast and blur-free imaging of organoids. *Sci. Rep.* **13**, 11268 (2023).

111. K. Moshksayan, R. Khanna, B. Camli, Z. N. Yakay, A. Harihara, C. Zdyrski, S. Mondal, E. Hegarty, J. P. Mochel, K. Allenspach, A microfluidic platform for culturing and high-content imaging of adult stem cell-derived organoids. *Sci. Rep.* **15**, 40162 (2025).

112. M. Westerfield, *The Zebrafish: A Guide for the Laboratory Use of Zebrafish (Brachydanio Reriro)* (Institute of Neuroscience, Univ. of Oregon, 1993).

113. C. B. Kimmel, W. W. Ballard, S. R. Kimmel, B. Ullmann, T. F. Schilling, Stages of embryonic development of the zebrafish. *Dev. Dyn.* **203**, 253–310 (1995).

114. N. Perraudin, V. Kalofolias, D. Shuman, P. Vandergheynst, UNLocBoX: A MATLAB convex optimization toolbox for proximal-splitting methods. arXiv:1402.0779 [cs.LG] (2014).

115. S. Osher, M. Burger, D. Goldfarb, J. Xu, W. Yin, An iterative regularization method for total variation-based image restoration. *Multiscale Model. Sim.* **4**, 460–489 (2005).

116. N. Sultanova, S. Kasarova, I. Nikolov, Dispersion properties of optical polymers. *Acta Phys. Pol. A* **116**, 585–587 (2009).

117. D. P. Kingma, J. Ba, Adam: A method for stochastic optimization. arXiv:1412.6980 [cs.LG] (2014).

118. X. Chen, S. Liu, R. Sun, M. Hong, On the convergence of a class of adam-type algorithms for non-convex optimization. arXiv:1808.02941 [cs.LG] (2018).

119. S. De, A. Mukherjee, E. Ullah, Convergence guarantees for RMSProp and ADAM in non-convex optimization and an empirical comparison to Nesterov acceleration. arXiv:1807.06766 [cs.LG] (2018).

120. A. Paszke, S. Gross, F. Massa, A. Lerer, J. Bradbury, G. Chanan, T. Killeen, Z. Lin, N. Gimelshein, L. Antiga, Pytorch: An imperative style, high-performance deep learning library. *Adv. Neural Inf. Proces. Syst.* **32**, 8024–8035 (2019).
